# 6,7-Dimethoxy-2-phenethyl-1,2,3,4-tetrahydroisoquinoline amides and corresponding ester isosteres as multidrug resistance reversers

**DOI:** 10.1080/14756366.2020.1747449

**Published:** 2020-04-07

**Authors:** Laura Braconi, Gianluca Bartolucci, Marialessandra Contino, Niccolò Chiaramonte, Roberta Giampietro, Dina Manetti, Maria Grazia Perrone, Maria Novella Romanelli, Nicola Antonio Colabufo, Chiara Riganti, Silvia Dei, Elisabetta Teodori

**Affiliations:** a NEUROFARBA Department, Section of Pharmaceutical and Nutraceutical Sciences, University of Florence, Florence, Italy; b Department of Pharmacy-Drug Sciences, University of Bari “A. Moro”, Bari, Italy; c Department of Oncology, University of Turin, Turin, Italy

**Keywords:** Tetrahydroisoquinoline, MDR reversers, P-gp modulators, co-administration assay, cytotoxicity

## Abstract

Aiming to deepen the structure–activity relationships of the two P-glycoprotein (P-gp) modulators elacridar and tariquidar, a new series of amide and ester derivatives carrying a 6,7-dimethoxy-2-phenethyl-1,2,3,4-tetrahydroisoquinoline scaffold linked to different methoxy-substituted aryl moieties were synthesised. The obtained compounds were evaluated for their P-gp interaction profile and selectivity towards the two other ABC transporters, multidrug-resistance-associated protein-1 and breast cancer resistance protein, showing to be very active and selective versus P-gp. Two amide derivatives, displaying the best P-gp activity, were tested in co-administration with the antineoplastic drug doxorubicin in different cancer cell lines, showing a significant sensitising activity towards doxorubicin. The investigation on the chemical stability of the derivatives towards spontaneous or enzymatic hydrolysis, showed that amides are stable in both models while some ester compounds were hydrolysed in human plasma. This study allowed us to identify two chemosensitizers that behave as non-transported substrates and are characterised by different selectivity profiles.

## Graphical Abstract

**Figure:**
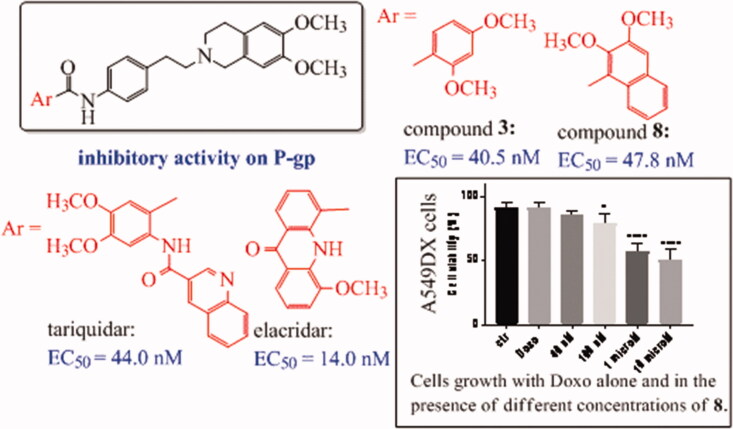

## Introduction

The chemotherapy against cancer is often undermined by resistance that tumour cells develop to cytotoxic compounds after an exposure period. Multidrug resistance (MDR) was described as acquired resistance developed towards a multiplicity of structurally unrelated chemotherapeutic drugs^1^, to which cells could also have never been exposed^2^. The MDR phenotype is often related to an enhanced energy-dependent drug efflux, elicited by the overexpression of transmembrane proteins that extrude substrates in a unidirectional way and reduce the concentration of antitumor drugs below the active dose. In human MDR cells, these proteins are part of the ATP-binding cassette (ABC) transporter family^3^^,^^4^, and use the energy deriving from ATP hydrolysis for this active transport.

In addition to be overexpressed in cancer cells, these proteins are also present in many healthy tissues where they arouse different physiological and pharmacological actions^5^, by regulating the permeability of biological membranes. In fact, they are often involved in the protection of these tissues from xenobiotics and can influence ADME properties and bioavailability of many drugs. The human genome encodes 49 ABC transmembrane proteins, divided into seven subfamilies (ABC-A to ABC-G), based on the similarity of their amino acid sequences^6^. Three transport proteins: P-glycoprotein (P-gp, ABCB1)^7^^,^^8^, multidrug-resistance-associated protein-1 (MRP1, ABCC1)^9^^,^^10^, and breast cancer resistance protein (BCRP, ABCG2)^11^^,^^12^ have mainly been associated with MDR^13–15^.

Studies on P-gp showed the first evidences of a relationship between its overexpression and tumour responses to cytotoxic drugs; then also MRP1 and BCRP overexpression^16^^,^^17^ was highlighted in different kind of tumours. Evidences also suggest that these three pumps can be co-expressed^18^.

Since the discovery of P-gp, the identification of compounds that act as inhibitors of P-gp and then of the sister proteins has been considered a possible way to fight against MDR. These compounds can be defined as chemo-sensitizers or MDR reversers; they should restore the efficacy of cytotoxic agents in resistant cancer cells if co-administered with the drugs which are substrates of the transporters^19^.

A great number of compounds showing P-gp modulating activity has been synthesised and studied, and were classified as first, second- or third-generation^20^^,^^21^ but none of these compounds has overcome the clinical trials, because no substantial benefits have been established. In fact, they often showed a low potency; moreover, they interacted with CYP4503A4 and altered the pharmacokinetic profiles of the co-administered antitumor agents, leading therefore also to increased toxicity^22^^,^^23^, although some of the latest MDR-reversing compounds show a safer profile. Therefore, aiming to overcome the obstacles described above, the search for safer ABC-transporter dependent MDR modulators is still pursued.

Two of the most interesting third-generation chemo-sensitizers are the tetrahydroisoquinoline derivatives elacridar (GF120918 or GW120918)^24^ and tariquidar (XR9576)^25^. These compounds displayed a high affinity towards the ABC proteins and a reduced effect on cytochromes, and few pharmacokinetic interactions with the cytotoxic drugs^26^. Disappointingly, clinical trials^27^ failed to show an improvement of the efficacy of antitumor agents after co-administration of these compounds^28^^,^^29^, and they have not been approved for therapy.

**Chart 1.**
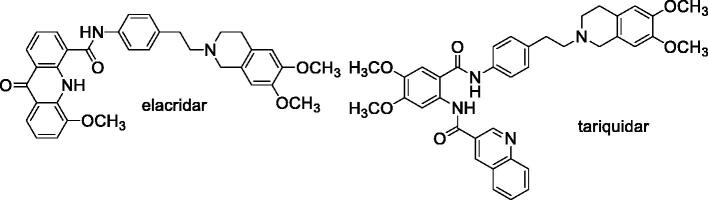
Tariquidar and elacridar.

Elacridar and tariquidar share a common structural characteristic, the presence of a 6,7-dimethoxy-2-phenethyl-1,2,3,4-tetrahydroisoquinoline moiety connected to an aryl substituted amide function (Chart 1). Early studies indicated that both derivatives are not specific for P-gp because they are also able to bind the BCRP transporter^30^. Subsequent structure-activity relationship studies suggested that the 6,7-dimethoxy-2-phenethyl-1,2,3,4-tetrahydroisoquinoline nucleus is essential for inhibiting the two transporter proteins P-gp and BCRP while changes at the aryl-substituted amide moiety elicit variations in the selectivity^31^. In the case of tariquidar, recent evidences indicated that this molecule is able to bind also the MRP1 transporter^32^; the same compound was shown to potentiate the sensitivity to paclitaxel in resistant cells transfected by another member of the ABCC family, the MRP7 protein^33^.

Therefore, despite the failure in clinical trials, tariquidar and elacridar have been considered lead compounds in the search for new MDR modulators, and a large number of analogues of these two derivatives have been synthesised.

**Figure 1.**
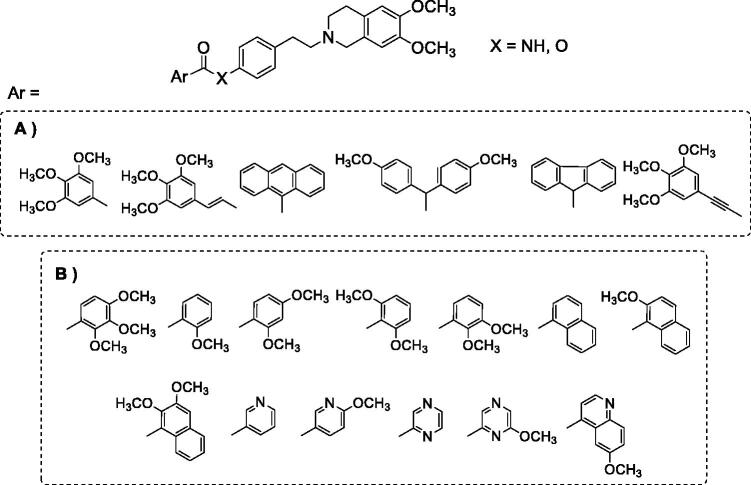
(A) Compounds described in the previous study^34^; (B) compounds synthesised in the present work.

Our research is part of this field of interest and aims to achieve information about the structural characteristics that confer potency and/or selectivity towards this family of transporters. In a previous study^34^, we reported a new series of derivatives bearing the 6,7-dimethoxy-2-phenethyl-1,2,3,4-tetrahydroisoquinoline moiety linked, as elacridar and tariquidar, to an aryl substituted amide, on which different aryl nucleus have been inserted (Figure 1); we also synthesised the corresponding isosteric ester derivatives. The aryl residues were selected based on their presence in other compounds bearing different skeletons, acting as very potent MDR reversers^35–40^. The obtained compounds were studied to evaluate their P-gp interaction profile and selectivity towards MPR1 and BCRP, using Madin-Darby Canine Kidney (MDCK) transfected cells.

Both amide and ester derivatives were active, although less potent with respect to lead derivatives and, in general, more selective towards P-gp. Interestingly, biological data indicated that in these series of tetrahydroisoquinoline derivatives the presence of an amide function was not essential for modulating the transporter proteins; and the presence of an ester function in place of an amide seemed to influence P-gp selectivity. As a continuation of this study, aiming to better evaluate the features for selectivity towards the three transporters and to deepen the structure–activity relationships of the series, we designed and synthesised a new series of amide and ester derivatives characterised by the presence of a 6,7-dimethoxy-2-phenethyl-1,2,3,4-tetrahydroisoquinoline scaffold linked to additional different aryl moieties (Figure 1). The new aryl moieties are methoxy-substituted phenyl or naphthyl nuclei, or nitrogen-containing hetero-aromatic residues.

The synthesised compounds were evaluated for their P-gp interaction profile and selectivity towards the two other ABC transporters, MPR1 and BCRP. The P-gp interacting-mechanism was investigated combining three assays: (i) apparent permeability (*P*_app_) determination (BA/AB) in Caco-2 cell monolayer; (ii) ATP cell depletion in cells overexpressing the transporter (MDCK-MDR1); (iii) the inhibition of Calcein-AM transport in MDCK-MDR1 cells. The activity on MRP1 and BCRP was evaluated on cancer cell lines overexpressing each transporter (MDCK-MRP1 and MDCK-BCRP cells), by measuring the inhibition of the efflux of the pro-fluorescent probe Calcein-AM in MDCK-MRP1 cells or the fluorescent probe Hoechst 33342 in MDCK-BCRP cells.

Furthermore two selected compounds were further tested alone and in co-administration with the antineoplastic drug doxorubicin in different cancer cell lines with various levels of P-gp.

Finally, the stability of amide and ester derivatives was investigated in phosphate buffer solution (PBS) and human plasma, and the degradation profiles of the molecules were evaluated.

## Material and methods

### Chemistry

All melting points were taken on a Büchi apparatus and are uncorrected. NMR spectra were recorded on a Bruker Avance 400 spectrometer (400 MHz for ^1^H-NMR, 100 MHz for ^13 ^C-NMR). ^1^H and ^13 ^C NMR spectra were measured at room temperature (25 °C) in an appropriate solvent. ^1^H and ^13 ^C chemical shifts are expressed in ppm (δ) referenced to TMS. Spectral data are reported using the following abbreviations: s = singlet, d = doublet, dd = doublet of doublets, t = triplet, td = triplet of doublets, m = multiplet, and coupling constants are reported in Hz, followed by integration.

Chromatographic separations were performed on a silica gel column by gravity chromatography (Kieselgel 40, 0.063–0.200 mm; Merck) or flash chromatography (Kieselgel 40, 0.040–0.063 mm; Merck). Yields are given after purification, unless otherwise stated.

The high resolution mass spectrometry (HRMS) analysis was performed with a Thermo Finnigan LTQ Orbitrap mass spectrometer equipped with an electrospray ionisation source (ESI). The accurate mass measure was carried out by introducing, via syringe pump at 10 μL min^−1^, the sample solution (1.0 μg mL^−1^ in mQ water: acetonitrile 50:50), and the signal of the positive ions was acquired. The proposed experimental conditions allowed to monitoring the protonated molecules of studied compounds ([M + H]^+^ species), that they were measured with a proper dwell time to achieve 60,000 units of resolution at Full Width at Half Maximum (FWHM). The elemental composition of compounds was calculated on the basis of their measured accurate masses, accepting only results with an attribution error less than 2.5 ppm and a not integer RDB (double bond/ring equivalents) value, in order to consider only the protonated species^41^.

Compounds were named following IUPAC rules as applied by ChemBioDraw Ultra 14.0 software. When reactions were performed in anhydrous conditions, the mixtures were maintained under nitrogen. Free bases **1–26** were transformed into the hydrochloride by treatment with a solution of acetyl chloride (1.1 eq) in anhydrous CH_3_OH. The salts were crystallised from abs. ethanol/petroleum ether.

### General procedures for the synthesis of the amide derivatives 1–13

Compounds were synthesised using two different general procedures.

*General procedure* ***A***. To a solution of the aniline **27**^34^ (0.25 mmol) in 5 ml of an. CH_2_Cl_2_ or an. CH_3_CN at 0 °C, the proper carboxylic acid (0.30 mmol), DMAP (0.20 mmol) and EDC hydrochloride (0.45 mmol) were added. The reaction mixture was stirred at 0 °C for 1 h, then at room temperature for 48 h. The mixture was treated with CH_2_Cl_2_ and the organic layer was washed with water and with a saturated solution of NaHCO_3_. After drying with Na_2_SO_4_, the solvent was removed under reduced pressure. The residue was then purified by flash chromatography using the appropriate eluting system, obtaining the desired compound as an oil.

*General procedure* ***B***. The proper carboxylic acid (0.30 mmol) was transformed into the acyl chloride by reaction with SOCl_2_ (4 eq) in 5 ml of CHCl_3_ (free of ethanol) or an. CH_3_CN at 60 °C for 6–8 h. The reaction mixture was cooled to rt, and the solvent was removed under reduced pressure. The mixture was then treated twice with cyclohexane and the solvent removed under reduced pressure. The acyl chloride obtained was dissolved in CHCl_3_ (free of ethanol), and the aniline **27**^34^ (0.25 mmol) was added. The mixture was kept at rt for 24 h, then the organic layer was washed twice with a saturated solution of NaHCO_3_. After drying with Na_2_SO_4_, the solvent was removed under reduced pressure. The residue obtained was purified by flash chromatography using the appropriate eluting system, yielding the desired compound as an oil.

In both procedures **A** and **B**, all the compounds were transformed into the corresponding hydrochloride as solid. The salts were crystallised from abs. ethanol/petroleum ether.

#### N-(4–(2-(6,7-dimethoxy-3,4-dihydroisoquinolin-2(1H)-yl)ethyl)phenyl)-2,3,4-trimethoxybenzamide (1)

Procedure **B** in CHCl_3_ (free of ethanol), starting from aniline **27** and 2,3,4-trimethoxybenzoic acid.

**Free base:** chromatographic eluent: CH_2_Cl_2_/MeOH/NH_4_OH 97:3:0.3. Yield: 15.1%.

^1^H NMR (400 MHz, CDCl_3_) δ: 9.92 (s, 1H, NH); 7.98 (d, *J* = 8.8 Hz, 1H, CH arom.); 7.60 (d, *J* = 8.4 Hz, 2H, CH arom.); 7.23 (d, *J* = 8.4 Hz, 2H, CH arom.); 6.82 (d, *J* = 8.8 Hz, 1H, CH arom.); 6.60 (s, 1H, CH arom.); 6.53 (s, 1H, CH arom.); 4.05 (s, 3H, OCH_3_); 3.92 (s, 3H, OCH_3_); 3.90 (s, 3H, OCH_3_); 3.84 (s, 3H, OCH_3_); 3.83 (s, 3H, OCH_3_); 3.69 (s, 2H, NCH_2_Ar); 2.96–2.89 (m, 2H, CH_2_); 2.88–2.75 (m, 6H, CH_2_) ppm. ^13 ^C NMR (100 MHz, CDCl_3_) δ: 162.71 (C=O); 156.78 (C); 152.14 (C); 147.66 (C); 147.32 (C); 136.69 (C); 135.84 (C); 129.26 (CH arom.); 126.96 (CH arom.); 125.97 (C); 125.91 (C); 120.33 (CH arom.); 119.00 (C); 111.38 (CH arom.); 109.49 (CH arom.); 107.89 (CH arom.); 61.97 (OCH_3_); 61.05 (OCH_3_); 59.93 (CH_2_); 56.10 (OCH_3_); 55.95 (OCH_3_); 55.91 (OCH_3_); 55.46 (CH_2_); 50.91 (CH_2_); 33.24 (CH_2_); 28.35 (CH_2_) ppm. ESI-HRMS (*m/z*) calculated for [M + H]^+^ ion species C_29_H_35_N_2_O_6_= 507.2490, found 507.2492.

**Hydrochloride:** mp 208–211 °C.

#### N-(4–(2-(6,7-dimethoxy-3,4-dihydroisoquinolin-2(1H)-yl)ethyl)phenyl)-2-methoxybenzamide (2)

Procedure **A** in an. CH_2_Cl_2_, starting from aniline **27** and 2-methoxybenzoic acid.

**Free base:** chromatographic eluent: CH_2_Cl_2_/MeOH/NH_4_OH 97:3:0.3. Yield: 88.9%.

^1^H NMR (400 MHz, CDCl_3_) δ: 9.71 (s, 1H, NH); 8.22 (d, *J* = 8.0 Hz, 1H, CH arom.); 7.56 (d, *J* = 8.4 Hz, 2H, CH arom.); 7.41 (t, *J* = 8.0 Hz, 1H, CH arom.); 7.18 (d, *J* = 8.4 Hz, 2H, CH arom.); 7.06 (t, *J* = 8.0 Hz, 1H, CH arom.); 6.95 (d, *J* = 8.0 Hz, 1H, CH arom.); 6.55 (s, 1H, CH arom.); 6.49 (s, 1H, CH arom.); 3.96 (s, 3H, OCH_3_); 3.79 (s, 3H, OCH_3_); 3.78 (s, 3H, OCH_3_); 3.60 (s, 2H, NCH_2_Ar); 2.90–2.67 (m, 8H, CH_2_) ppm. ^13^C NMR (100 MHz, CDCl_3_) δ: 163.13 (C=O); 157.19 (C); 147.59 (C); 147.27 (C); 136.50 (C); 136.09 (C); 133.16 (CH arom.); 132.39 (CH arom.); 129.17 (CH arom.); 126.29 (C); 126.06 (C); 121.83 (C); 121.59 (CH arom.); 120.58 (CH arom.); 111.57 (CH arom.); 111.44 (CH arom.); 109.56 (CH arom.); 60.03 (CH_2_); 56.21 (OCH_3_); 55.95 (OCH_3_); 55.91 (OCH_3_); 55.56 (CH_2_); 50.96 (CH_2_); 33.33 (CH_2_); 28.53 (CH_2_) ppm. ESI-HRMS (*m/z*) calculated for [M + H]^+^ ion species C_27_H_31_N_2_O_4_= 447.2278, found 447.2279.

**Hydrochloride:** mp 226–228 °C.

#### N-(4–(2-(6,7-dimethoxy-3,4-dihydroisoquinolin-2(1H)-yl)ethyl)phenyl)-2,4-dimethoxybenzamide (3)

Procedure **B** in CHCl_3_ (free of ethanol), starting from aniline **27** and 2,4-dimethoxybenzoic acid.

**Free base:** chromatographic eluent: CH_2_Cl_2_/MeOH/NH_4_OH 97:3:0.3. Yield: 54.7%.

^1^H NMR (400 MHz, CDCl_3_) δ: 9.69 (s, 1H, NH); 8.26 (d, *J* = 8.8 Hz, 1H, CH arom.); 7.60 (d, *J* = 8.4 Hz, 2H, CH arom.); 7.23 (d, *J* = 8.4 Hz, 2H, CH arom.); 6.66 (dd, *J* = 8.8 Hz, 2.2 Hz, 1H, CH arom.); 6.62 (s, 1H, CH arom.); 6.55 (s, 1H, CH arom.); 6.54 (d, *J* = 2.2 Hz, 1H, CH arom.); 4.03 (s, 3H, OCH_3_); 3.88 (s, 3H, OCH_3_); 3.86 (s, 3H, OCH_3_); 3.85 (s, 3H, OCH_3_); 3.73 (s, 2H, NCH_2_Ar); 3.00–2.94 (m, 2H, CH_2_); 2.93–2.77 (m, 6H, CH_2_) ppm. ^13^C NMR (100 MHz, CDCl_3_) δ: 163.70 (C=O); 163.04 (C); 158.51 (C); 147.66 (C); 147.31 (C); 136.78 (C); 135.43 (C); 134.16 (CH arom.); 129.18 (CH arom.); 125.71 (C); 120.57 (CH arom.); 114.67 (C); 111.27 (CH arom.); 109.38 (CH arom.); 105.65 (CH arom.); 98.74 (CH arom.); 59.80 (CH_2_); 56.20 (OCH_3_); 55.93 (OCH_3_); 55.89 (OCH_3_); 55.59 (OCH_3_); 55.30 (CH_2_); 50.85 (CH_2_); 33.11 (CH_2_); 28.16 (CH_2_) ppm. ESI-HRMS (*m/z*) calculated for [M + H]^+^ ion species C_28_H_33_N_2_O_5_= 477.2384, found 477.2379.

**Hydrochloride:** mp 233–235 °C.

#### N-(4–(2-(6,7-dimethoxy-3,4-dihydroisoquinolin-2(1H)-yl)ethyl)phenyl)-2,6-dimethoxybenzamide (4)

Procedure **B** in CHCl_3_ (free of ethanol), starting from aniline **27** and 2,6-dimethoxybenzoic acid.

**Free base:** chromatographic eluent: CH_2_Cl_2_/MeOH 95:5. Yield: 75.0%.

^1^H NMR (400 MHz, CDCl_3_) δ: 7.53 (d, *J* = 8.4 Hz, 2H, CH arom.); 7.50 (s, 1H, NH); 7.24 (t, *J* = 8.4 Hz, 1H, CH arom.); 7.15 (d, *J* = 8.4 Hz, 2H, CH arom.); 6.55 (s, 1H, CH arom.); 6.53 (d, *J* = 8.4 Hz, 2H, CH arom.); 6.48 (s, 1H, CH arom.); 3.79 (s, 3H, OCH_3_); 3.78 (s, 3H, OCH_3_); 3.76 (s, 6H, OCH_3_); 3.63 (s, 2H, NCH_2_Ar); 2.90–2.70 (m, 8H, CH_2_) ppm. ^13^C NMR (100 MHz, CDCl_3_) δ: 163.65 (C=O); 157.55 (C); 147.64 (C); 147.31 (C); 136.58 (C); 135.85 (C); 131.03 (CH arom.); 129.13 (CH arom.); 125.91 (C); 119.82 (CH arom.); 116.00 (C); 111.39 (CH arom.); 109.52 (CH arom.); 104.11 (CH arom.); 59.92 (CH_2_); 56.02 (OCH_3_); 55.94 (OCH_3_); 55.90 (OCH_3_); 55.42 (CH_2_); 50.90 (CH_2_); 33.17 (CH_2_); 28.34 (CH_2_) ppm. ESI-HRMS (*m/z*) calculated for [M + H]^+^ ion species C_28_H_33_N_2_O_5_= 477.2384, found 477.2384.

**Hydrochloride:** mp 212–214 °C.

#### N-(4–(2-(6,7-dimethoxy-3,4-dihydroisoquinolin-2(1H)-yl)ethyl)phenyl)-2,3-dimethoxybenzamide (5)

Procedure **B** in CHCl_3_ (free of ethanol), starting from aniline **27** and 2,3-dimethoxybenzoic acid.

**Free base:** chromatographic eluent: CH_2_Cl_2_/MeOH/NH_4_OH 97:3:0.3. Yield: 65.6%.

^1^H NMR (400 MHz, CDCl_3_) δ: 9.93 (s, 1H, NH); 7.73 (dd, *J* = 8.0, 1.6 Hz, 1H, CH arom.); 7.58 (d, *J* = 8.4 Hz, 2H, CH arom.); 7.20 (d, *J* = 8.4 Hz, 2H, CH arom.); 7.15 (t, *J* = 8.0 Hz, 1H, CH arom.); 7.03 (dd, *J* = 8.0, 1.6 Hz, 1H, CH arom.); 6.56 (s, 1H, CH arom.); 6.50 (s, 1H, CH arom.); 3.93 (s, 3H, OCH_3_); 3.87 (s, 3H, OCH_3_); 3.80 (s, 3H, OCH_3_); 3.79 (s, 3H, OCH_3_); 3.62 (s, 2H, NCH_2_Ar); 2.90–2.84 (m, 2H, CH_2_); 2.83–2.69 (m, 6H, CH_2_) ppm. ^13^C NMR (100 MHz, CDCl_3_) δ: 162.90 (C=O); 152.61 (C); 147.57 (C); 147.25 (C); 147.20 (C); 136.51 (C); 136.28 (C); 129.29 (CH arom.); 126.93 (C); 126.40 (C); 126.10 (C); 124.74 (CH arom.); 122.93 (CH arom.); 120.28 (CH arom.); 115.70 (CH arom.); 111.40 (CH arom.); 109.52 (CH arom.); 61.65 (OCH_3_); 60.13 (CH_2_); 56.14 (OCH_3_); 55.94 (OCH_3_); 55.91 (OCH_3_); 55.64 (CH_2_); 51.00 (CH_2_); 33.40 (CH_2_); 28.62 (CH_2_) ppm. ESI-HRMS (*m/z*) calculated for [M + H]^+^ ion species C_28_H_33_N_2_O_5_= 477.2384, found 477.2375.

**Hydrochloride:** mp 230–233 °C.

#### N-(4–(2-(6,7-dimethoxy-3,4-dihydroisoquinolin-2(1H)-yl)ethyl)phenyl)-1-naphthamide (6)

Procedure **B** in CHCl_3_ (free of ethanol), starting from aniline **27** and 1-naphthoic acid.

**Free base:** chromatographic eluent: CH_2_Cl_2_/MeOH/NH_4_OH 97:3:0.3. Yield: 53.6%.

^1^H NMR (400 MHz, CDCl_3_) δ: 8.29–8.27 (m, 1H, CH arom.); 7.95 (s, 1H, NH); 7.87 (d, *J* = 7.6 Hz, 1H, CH arom.); 7.84–7.81 (m, 1H, CH arom.); 7.62 (d, *J* = 7.6 Hz, 1H, CH arom.); 7.56 (d, *J* = 8.4 Hz, 2H, CH arom.); 7.51–7.46 (m, 2H, CH arom.); 7.38 (t, *J* = 7.6 Hz, 1H, CH arom.); 7.19 (d, *J* = 8.4 Hz, 2H, CH arom.); 6.56 (s, 1H, CH arom.); 6.49 (s, 1H, CH arom.); 3.79 (s, 3H, OCH_3_); 3.78 (s, 3H, OCH_3_); 3.63 (s, 2H, NCH_2_Ar); 2.92–2.85 (m, 2H, CH_2_); 2.84–2.68 (m, 6H, CH_2_) ppm. ^13^C NMR (100 MHz, CDCl_3_) δ: 167.58 (C=O); 147.60 (C); 147.28 (C); 136.67 (C); 136.24 (C); 134.49 (C); 133.72 (C); 130.91 (CH arom.); 130.10 (C); 129.31 (CH arom.); 128.40 (CH arom.); 127.27 (CH arom.); 126.53 (CH arom.); 126.28 (C); 126.07 (C); 125.30 (CH arom.); 125.10 (CH arom.); 124.71 (CH arom.); 120.29 (CH arom.); 111.42 (CH arom.); 109.54 (CH arom.); 60.05 (CH_2_); 55.94 (OCH_3_); 55.90 (OCH_3_); 55.61 (CH_2_); 51.00 (CH_2_); 33.36 (CH_2_); 28.56 (CH_2_) ppm. ESI-HRMS (*m/z*) calculated for [M + H]^+^ ion species C_30_H_31_N_2_O_3_= 467.2329, found 467.2320.

**Hydrochloride:** mp 218–220 °C.

#### N-(4–(2-(6,7-dimethoxy-3,4-dihydroisoquinolin-2(1H)-yl)ethyl)phenyl)-2-methoxy-1-naphthamide (7)

Procedure **A** in an. CH_2_Cl_2_, starting from aniline **27** and 2-methoxy-1-naphthoic acid.

**Free base:** chromatographic eluent: CH_2_Cl_2_/MeOH 95:5. Yield: 30.5%.

^1^H NMR (400 MHz, CDCl_3_) δ: 7.99 (d, *J* = 8.0 Hz, 1H, CH arom); 7.84 (d, *J* = 8.8 Hz, 1H, CH arom.); 7.81 (s, 1H, NH); 7.75 (d, *J* = 8.0 Hz, 1H, CH arom.); 7.60 (d, *J* = 8.4 Hz, 2H, CH arom.); 7.44 (t, *J* = 8.0 Hz, 1H, CH arom.); 7.33 (t, *J* = 8.0 Hz, 1H, CH arom.); 7.22 (d, *J* = 8.8 Hz, 1H, CH arom.); 7.21 (d, *J* = 8.4 Hz, 2H, CH arom.); 6.56 (s, 1H, CH arom.); 6.50 (s, 1H, CH arom.); 3.91 (s, 3H, OCH_3_); 3.80 (s, 3H, OCH_3_); 3.79 (s, 3H, OCH_3_); 3.63 (s, 2H, NCH_2_Ar); 2.95–2.86 (m, 2H, CH_2_); 2.85–2.69 (m, 6H, CH_2_) ppm. ^13^C NMR (100 MHz, CDCl_3_) δ: 165.45 (C=O); 153.71 (C); 147.60 (C); 147.28 (C); 136.45 (C); 131.57 (CH arom.); 129.28 (CH arom.); 128.85 (C); 128.03 (CH arom.); 127.66 (CH arom.); 126.38 (C); 126.11 (C); 124.34 (CH arom.); 124.25 (CH arom.); 120.51 (C); 120.07 (CH arom.); 113.08 (CH arom.); 111.44 (CH arom.); 109.57 (CH arom.); 60.12 (CH_2_); 56.77 (OCH_3_); 55.95 (OCH_3_); 55.91 (OCH_3_); 55.63 (CH_2_); 51.01 (CH_2_); 33.37 (CH_2_); 28.60 (CH_2_) ppm. ESI-HRMS (*m/z*) calculated for [M + H]^+^ ion species C_31_H_33_N_2_O_4_= 497.2435, found 497.2425.

**Hydrochloride:** mp 239–241 °C.

#### N-(4–(2-(6,7-dimethoxy-3,4-dihydroisoquinolin-2(1H)-yl)ethyl)phenyl)-2,3-dimethoxy-1-naphthamide (8)

Procedure **A** in an. CH_2_Cl_2_, starting from aniline **27** and 2,3-dimethoxy-1-naphthoic acid.

**Free base:** chromatographic eluent: CH_2_Cl_2_/MeOH 95:5. Yield: 24.1%.

^1^H NMR (400 MHz, CDCl_3_) δ: 7.98 (d, *J* = 8.0 Hz, 1H, CH arom.); 7.90 (s, 1H, NH), 7.67 (d, *J* = 8.0 Hz, 1H, CH arom.); 7.61 (d, *J* = 8.4 Hz, 2H, CH arom.); 7.42–7.33 (m, 2H, CH arom.); 7.24 (d, *J* = 8.4 Hz, 2H, CH arom.); 7.10 (s, 1H, CH arom.); 6.57 (s, 1H, CH arom.); 6.51 (s, 1H, CH arom.); 3.93 (s, 3H, OCH_3_); 3.88 (s, 3H, OCH_3_); 3.82 (s, 3H, OCH_3_); 3.81 (s, 3H, OCH_3_); 3.66 (s, 2H, NCH_2_Ar); 2.95–2.88 (m, 2H, CH_2_); 2.87–2.74 (m, 6H, CH_2_) ppm. ^13^C NMR (100 MHz, CDCl_3_) δ: 164.75 (C=O); 151.32 (C); 147.63 (C); 147.30 (C); 146.22 (C); 136.57 (C); 136.27 (C); 131.39 (C); 129.38 (CH arom.); 127.22 (C); 126.73 (CH arom.); 126.03 (C); 125.96 (CH arom.); 125.12 (CH arom.); 124.71 (CH arom.); 120.17 (CH arom.); 111.37 (CH arom.); 109.49 (CH arom.); 108.85 (CH arom.); 62.40 (OCH_3_); 60.01 (CH_2_); 55.95 (OCH_3_); 55.91 (OCH_3_); 55.76 (OCH_3_); 55.55 (CH_2_); 50.97 (CH_2_); 33.32 (CH_2_); 28.47 (CH_2_) ppm. ESI-HRMS (*m/z*) calculated for [M + H]^+^ ion species C_32_H_35_N_2_O_5_= 527.2541, found 527.2534.

**Hydrochloride:** mp 245–248 °C.

#### N-(4–(2-(6,7-dimethoxy-3,4-dihydroisoquinolin-2(1H)-yl)ethyl)phenyl)nicotinamide (9)

Procedure **B** in an. CH_3_CN, starting from aniline **27** and nicotinic acid.

**Free base:** chromatographic eluent: CH_2_Cl_2_/MeOH/NH_4_OH 93:7:0.3. Yield: 40.5%.

^1^H NMR (400 MHz, CDCl_3_) δ: 9.02 (s, 1H, NH); 8.70 (s, 1H, CH arom.); 8.62 (d, *J* = 4.4 Hz, 1H, CH arom.); 8.13 (d, *J* = 8.0 Hz, 1H, CH arom.); 7.52 (d, *J* = 8.4 Hz, 2H, CH arom.); 7.30 (dd, *J* = 8.0, 4.4 Hz, 1H, CH arom.); 7.16 (d, *J* = 8.4 Hz, 2H, CH arom.); 6.54 (s, 1H, CH arom.); 6.48 (s, 1H, CH arom.); 3.77 (s, 3H, OCH_3_); 3.76 (s, 3H, OCH_3_); 3.60 (s, 2H, NCH_2_Ar); 2.87–2.67 (m, 8H, CH_2_) ppm. ^13^C NMR (100 MHz, CDCl_3_) δ: 164.18 (C=O); 152.07 (CH arom.); 148.30 (CH arom.); 147.52 (C); 147.19 (C); 136.87 (C); 135.95 (C); 135.48 (CH arom.); 130.80 (C); 129.21 (CH arom.); 126.26 (C); 126.04 (C); 123.49 (CH arom.); 120.97 (CH arom.); 111.36 (CH arom.); 109.48 (CH arom.); 59.96 (CH_2_); 55.88 (OCH_3_); 55.84 (OCH_3_); 55.57 (CH_2_); 50.96 (CH_2_); 33.30 (CH_2_); 28.53 (CH_2_) ppm. ESI-HRMS (*m/z*) calculated for [M + H]^+^ ion species C_25_H_28_N_3_O_3_= 418.2125, found 418.2128.

**Hydrochloride:** mp 151–154 °C.

#### N-(4–(2-(6,7-dimethoxy-3,4-dihydroisoquinolin-2(1H)-yl)ethyl)phenyl)-6-methoxynicotinamide (10)

Procedure **B** in an. CH_3_CN, starting from aniline **27** and 6-methoxynicotinic acid.

**Free base:** chromatographic eluent: CH_2_Cl_2_/MeOH/NH_4_OH 97:3:0.3. Yield: 29.3%.

^1^H NMR (400 MHz, CDCl_3_) δ: 8.65 (s, 1H, CH arom.); 8.03 (d, *J* = 8.4 Hz, 1H, CH arom.); 7.89 (s, 1H, NH); 7.50 (d, *J* = 6.8 Hz, 2H, CH arom.); 7.18 (d, *J* = 6.8 Hz, 2H, CH arom.); 6.75 (d, *J* = 8.4 Hz, 1H, CH arom.); 6.56 (s, 1H, CH arom.); 6.50 (s, 1H, CH arom.); 3.95 (s, 3H, OCH_3_); 3.80 (s, 3H, OCH_3_); 3.79 (s, 3H, OCH_3_); 3.63 (s, 2H, NCH_2_Ar); 2.91–2.70 (m, 8H, CH_2_) ppm. ^13^C NMR (100 MHz, CDCl_3_) δ: 166.07 (C=O); 164.08 (C); 147.67 (C); 147.33 (C); 146.84 (CH arom.); 137.93 (CH arom.); 136.31 (C); 136.15 (C); 129.20 (CH arom.); 125.83 (C); 124.04 (C); 120.79 (CH arom.); 111.38 (CH arom.); 110.78 (CH arom.); 109.50 (CH arom.); 59.69 (CH_2_); 55.92 (OCH_3_); 55.89 (OCH_3_); 55.34 (CH_2_); 53.94 (OCH_3_); 50.82 (CH_2_); 33.08 (CH_2_); 28.25 (CH_2_) ppm. ESI-HRMS (*m/z*) calculated for [M + H]^+^ ion species C_26_H_30_N_3_O_4_= 448.2231, found 448.2236.

**Hydrochloride:** mp 123–126 °C.

#### N-(4–(2-(6,7-dimethoxy-3,4-dihydroisoquinolin-2(1H)-yl)ethyl)phenyl)pyrazine-2-carboxamide (11)

Procedure **A** in an. CH_2_Cl_2_, starting from aniline **27** and pyrazine-2-carboxylic acid.

**Free base:** chromatographic eluent: CH_2_Cl_2_/MeOH/NH_4_OH 97:3:0.3. Yield: 63.8%.

^1^H NMR (400 MHz, CDCl_3_) δ: 9.59 (s, 1H, NH); 9.45 (d, *J* = 1.2 Hz, 1H, CH arom.); 8.73 (d, *J* = 2.4 Hz, 1H, CH arom.); 8.51 (dd, *J* = 2.4, 1.2 Hz, 1H, CH arom.); 7.64 (d, *J* = 8.4 Hz, 2H, CH arom.); 7.22 (d, *J* = 8.4 Hz, 2H, CH arom.); 6.55 (s, 1H, CH arom.); 6.48 (s, 1H, CH arom.); 3.79 (s, 3H, OCH_3_); 3.78 (s, 3H, OCH_3_); 3.61 (s, 2H, NCH_2_Ar); 2.90–2.83 (m, 2H, CH_2_); 2.82–2.69 (m, 6H, CH_2_) ppm. ^13^C NMR (100 MHz, CDCl_3_) δ: 160.55 (C=O); 147.60 (C); 147.47 (CH arom.); 147.28 (C); 144.62 (CH arom.); 144.44 (C); 142.35 (CH arom.); 136.95 (C); 135.35 (C); 129.40 (CH arom.); 126.32 (C); 126.07 (C); 119.96 (CH arom.); 111.42 (CH arom.); 109.53 (CH arom.); 59.98 (CH_2_); 55.94 (OCH_3_); 55.90 (OCH_3_); 55.62 (CH_2_); 50.99 (CH_2_); 33.41 (CH_2_); 28.57 (CH_2_) ppm. ESI-HRMS (*m/z*) calculated for [M + H]^+^ ion species C_24_H_27_N_4_O_3_= 419.2078, found 419.2079.

**Hydrochloride:** mp 246–249 °C.

#### N-(4–(2-(6,7-dimethoxy-3,4-dihydroisoquinolin-2(1H)-yl)ethyl)phenyl)-6-methoxypyrazine-2-carboxamide (12)

Procedure **B** in an. CH_3_CN, starting from aniline **27** and 6-methoxypyrazine-2-carboxylic acid.

**Free base:** chromatographic eluent: CH_2_Cl_2_/MeOH/NH_4_OH 93:7:0.3. Yield: 89.5%.

^1^H NMR (400 MHz, CDCl_3_) δ: 9.32 (s, 1H, NH); 9.02 (s, 1H, CH arom.); 8.41 (s, 1H, CH arom.); 7.62 (d, *J* = 8.4 Hz, 2H, CH arom.); 7.24 (d, *J* = 8.4 Hz, 2H, CH arom.); 6.57 (s, 1H, CH arom.); 6.50 (s, 1H, CH arom.); 4.06 (s, 3H, OCH_3_); 3.81 (s, 3H, OCH_3_); 3.80 (s, 3H, OCH_3_); 3.65 (s, 2H, NCH_2_Ar); 2.95–2.87 (m, 2H, CH_2_); 2.86–2.73 (m, 6H, CH_2_) ppm. ^13^C NMR (100 MHz, CDCl_3_) δ: 160.76 (C=O); 140.43 (C); 139.35 (CH arom.); 138.65 (C); 136.53 (C); 136.14 (CH arom.); 135.37 (C); 132.67 (C); 132.13 (C); 129.45 (CH arom.); 125.67 (C); 125.45 (C); 120.13 (CH arom.); 111.31 (CH arom.); 109.42 (CH arom.); 59.58 (CH_2_); 55.95 (OCH_3_); 55.91 (OCH_3_); 55.29 (CH_2_); 53.89 (OCH_3_); 50.82 (CH_2_); 33.10 (CH_2_); 28.04 (CH_2_) ppm. ESI-HRMS (*m/z*) calculated for [M + H]^+^ ion species C_25_H_29_N_4_O_4_= 449.2183, found 449.2187.

**Hydrochloride:** mp 224–226 °C.

#### N-(4–(2-(6,7-dimethoxy-3,4-dihydroisoquinolin-2(1H)-yl)ethyl)phenyl)-6-methoxyquinoline-4-carboxamide (13)

Procedure **B** in CHCl_3_ (free of ethanol), starting from aniline **27** and 6-methoxyquinoline-4-carboxylic acid.

**Free base:** yield: 100.0%.

^1^H NMR (400 MHz, CDCl_3_) δ: 9.24 (s, 1H, NH); 8.35 (d, *J* = 4.0 Hz, 1H, CH arom.); 7.78 (d, *J* = 9.2 Hz, 1H, CH arom.); 7.68 (d, *J* = 8.0 Hz, 2H, CH arom.); 7.30 (d, *J* = 2.0 Hz, 1H, CH arom.); 7.24–7.20 (m, 3H, CH arom.); 7.13 (d, *J* = 4.0 Hz, 1H, CH arom.); 6.55 (s, 1H, CH arom.); 6.49 (s, 1H, CH arom.); 3.78 (s, 6H, OCH_3_); 3.76 (s, 3H, OCH_3_); 3.63 (s, 2H, NCH_2_Ar); 2.93–2.87 (m, 2H, CH_2_); 2.85–2.73 (m, 6H, CH_2_) ppm. ^13^C NMR (100 MHz, CDCl_3_) δ: 165.86 (C=O); 158.41 (C); 147.50 (C); 147.16 (C); 146.62 (CH arom.); 144.34 (C); 140.14 (C); 136.87 (C); 136.27 (C); 130.51 (CH arom.); 129.32 (CH arom.); 126.09 (C); 125.95 (C); 125.45 (C); 122.94 (CH arom.); 120.57 (CH arom.); 118.92 (CH arom.); 111.31 (CH arom.); 109.43 (CH arom.); 102.57 (CH arom.); 59.92 (CH_2_); 55.87 (OCH_3_); 55.83 (OCH_3_); 55.52 (OCH_3_); 55.48 (CH_2_); 50.88 (CH_2_); 33.21 (CH_2_); 28.42 (CH_2_) ppm. ESI-HRMS (*m/z*) calculated for [M + H]^+^ ion species C_30_H_32_N_3_O_4_= 498.2387, found 498.2395.

**Hydrochloride:** mp 228–230 °C.

### General procedures for the synthesis of the ester compounds 14–26

Compounds were synthesised using two different general procedures.

*General procedure* ***A***. To a solution of the phenol **28**^34^ (0.25 mmol) in 5 ml of an. CH_2_Cl_2_ or an. CH_3_CN at 0 °C, the proper carboxylic acid (0.30 mmol), DMAP (0.20 mmol) and EDC hydrochloride (0.45 mmol) were added. The reaction mixture was stirred at 0 °C for 1 h then at room temperature for 48 h. The mixture was treated with CH_2_Cl_2_ and the organic layer was washed with water and with a saturated solution of NaHCO_3_. After drying with Na_2_SO_4_, the solvent was removed under reduced pressure. The residue was then purified by flash chromatography using the appropriate eluting system, obtaining the desired compound as an oil.

*General procedure* ***B***. The proper carboxylic acid (0.30 mmol) was transformed into the acyl chloride by reaction with SOCl_2_ (4 eq) in 5 ml of CHCl_3_ (free of ethanol) or an. CH_3_CN at 60 °C for 6–8 h. The reaction mixture was cooled to rt, and the solvent was removed under reduced pressure. The mixture was then treated twice with cyclohexane and the solvent removed under reduce pressure. The acyl chloride obtained was dissolved in CHCl_3_ (free of ethanol), and the phenol **28**^34^ (0.25 mmol) was added. The mixture was kept at rt for 24 h, then the organic layer was washed twice with a saturated solution of NaHCO_3_. After drying with Na_2_SO_4_, the solvent was removed under reduced pressure. The residue obtained was purified by flash chromatography using the appropriate eluting system, yielding the desired compound as an oil.

In both procedures **A** and **B**, all the compounds were transformed into the corresponding hydrochloride as solid. The salts were crystallised from abs. ethanol/petroleum ether.

#### 4–(2-(6,7-Dimethoxy-3,4-dihydroisoquinolin-2(1H)-yl)ethyl)phenyl 2,3,4-trimethoxybenzoate (14)

Procedure **B** in CHCl_3_ (free of ethanol), starting from phenol **28** and 2,3,4-trimethoxybenzoic acid.

**Free base:** chromatographic eluent: CH_2_Cl_2_/MeOH/NH_4_OH 97:3:0.3. Yield: 17.2%.

^1^H NMR (400 MHz, CDCl_3_) δ: 7.79 (d, *J* = 8.8 Hz, 1H, CH arom.); 7.28 (d, *J* = 8.4 Hz, 2H, CH arom.); 7.13 (d, *J* = 8.4 Hz, 2H, CH arom.); 6.75 (d, *J* = 8.8 Hz, 1H, CH arom.); 6.60 (s, 1H, CH arom.); 6.53 (s, 1H, CH arom.); 3.97 (s, 3H, OCH_3_); 3.93 (s, 3H, OCH_3_); 3.89 (s, 3H, OCH_3_); 3.84 (s, 3H, OCH_3_); 3.83 (s, 3H, OCH_3_); 3.68 (s, 2H, NCH_2_Ar); 2.97–2.91 (m, 2H, CH_2_); 2.90–2.76 (m, 6H, CH_2_) ppm. ^13^C NMR (100 MHz, CDCl_3_) δ: 163.97 (C=O); 157.79 (C); 155.34 (C); 149.32 (C); 147.66 (C); 147.33 (C); 143.19 (C); 137.61 (C); 129.67 (CH arom.); 127.50 (CH arom.); 126.03 (C); 125.94 (C); 121.76 (CH arom.); 117.10 (C); 111.39 (CH arom.); 109.51 (CH arom.); 107.03 (CH arom.); 61.90 (OCH_3_); 61.06 (OCH_3_); 59.88 (CH_2_); 56.15 (OCH_3_); 55.95 (OCH_3_); 55.91 (OCH_3_); 55.50 (CH_2_); 50.96 (CH_2_); 33.25 (CH_2_); 28.40 (CH_2_) ppm. ESI-HRMS (*m/z*) calculated for [M + H]^+^ ion species C_29_H_34_NO_7_= 508.2330, found 508.2326.

**Hydrochloride:** mp 207–210 °C.

#### 4–(2-(6,7-Dimethoxy-3,4-dihydroisoquinolin-2(1H)-yl)ethyl)phenyl 2-methoxybenzoate (15)

Procedure **B** in CHCl_3_ (free of ethanol), starting from phenol **28** and 2-methoxybenzoic acid.

**Free base:** chromatographic eluent: CH_2_Cl_2_/MeOH/NH_4_OH 97:3:0.3. Yield: 44.6%.

^1^H NMR (400 MHz, CDCl_3_) δ: 7.95 (dd, *J* = 8.0, 1.8 Hz, 1H, CH arom.); 7.48 (td, *J* = 8.0, 1.8 Hz, 1H, CH arom.); 7.23 (d, *J* = 8.4 Hz, 2H, CH arom.); 7.11 (d, *J* = 8.4 Hz, 2H, CH arom.); 7.02–6.96 (m, 2H, CH arom.); 6.56 (s, 1H, CH arom.); 6.50 (s, 1H, CH arom.); 3.87 (s, 3H, OCH_3_); 3.79 (s, 3H, OCH_3_); 3.78 (s, 3H, OCH_3_); 3.61 (s, 2H, NCH_2_Ar); 2.93–2.85 (m, 2H, CH_2_); 2.84–2.70 (m, 6H, CH_2_) ppm. ^13^C NMR (100 MHz, CDCl_3_) δ: 164.53 (C=O); 159.79 (C); 149.31 (C); 147.62 (C); 147.30 (C); 137.70 (C); 134.22 (CH arom.); 132.09 (CH arom.); 129.59 (CH arom.); 126.31 (C); 126.07 (C); 121.72 (CH arom.); 120.21 (CH arom.); 119.28 (C); 112.27 (CH arom.); 111.46 (CH arom.); 109.58 (CH arom.); 59.97 (CH_2_); 56.05 (OCH_3_); 55.95 (OCH_3_); 55.92 (OCH_3_); 55.59 (CH_2_); 51.00 (CH_2_); 33.34 (CH_2_); 28.55 (CH_2_) ppm. ESI-HRMS (*m/z*) calculated for [M + H]^+^ ion species C_27_H_30_NO_5_= 448.2119, found 448.2123.

**Hydrochloride:** mp 220–222 °C.

#### 4–(2-(6,7-Dimethoxy-3,4-dihydroisoquinolin-2(1H)-yl)ethyl)phenyl 2,4-dimethoxybenzoate (16)

Procedure **B** in CHCl_3_ (free of ethanol), starting from phenol **28** and 2,4-dimethoxybenzoic acid.

**Free base:** chromatographic eluent: CH_2_Cl_2_/MeOH/NH_4_OH 97:3:0.3. Yield: 18.7%.

^1^H NMR (400 MHz, CDCl_3_) δ: 8.02 (d, *J* = 8.8 Hz, 1H, CH arom.); 7.23 (d, *J* = 8.4 Hz, 2H, CH arom.); 7.09 (d, *J* = 8.4 Hz, 2H, CH arom.); 6.57 (s, 1H, CH arom.); 6.52 (dd, *J* = 8.8, 2.2 Hz, 1H, CH arom.); 6.51 (s, 1H, CH arom.); 6.49 (d, *J* = 2.2 Hz, 1H, CH arom.); 3.87 (s, 3H, OCH_3_); 3.85 (s, 3H, OCH_3_); 3.81 (s, 3H, OCH_3_); 3.80 (s, 3H, OCH_3_); 3.66 (s, 2H, NCH_2_Ar); 2.96–2.89 (m, 2H, CH_2_); 2.88–2.74 (m, 6H, CH_2_) ppm. ^13^C NMR (100 MHz, CDCl_3_) δ: 164.90 (C=O); 163.83 (C); 162.15 (C); 149.52 (C); 147.80 (C); 147.44 (C); 136.84 (C); 134.43 (CH arom.); 129.55 (CH arom.); 125.51 (C); 121.97 (CH arom.); 111.33 (CH arom.); 109.44 (CH arom.); 104.79 (CH arom.); 99.04 (CH arom.); 59.45 (CH_2_); 56.03 (OCH_3_); 55.96 (OCH_3_); 55.92 (OCH_3_); 55.57 (OCH_3_); 55.11 (CH_2_); 50.77 (CH_2_); 32.91 (CH_2_); 27.87 (CH_2_) ppm. ESI-HRMS (*m/z*) calculated for [M + H]^+^ ion species C_28_H_32_NO_6_= 478.2224, found 478.2230.

**Hydrochloride:** mp 221–223 °C.

#### 4–(2-(6,7-Dimethoxy-3,4-dihydroisoquinolin-2(1H)-yl)ethyl)phenyl 2,6-dimethoxybenzoate (17)

Procedure **B** in CHCl_3_ (free of ethanol), starting from phenol **28** and 2,6-dimethoxybenzoic acid.

**Free base:** chromatographic eluent: CH_2_Cl_2_/MeOH/NH_4_OH 97:3:0.3. Yield: 37.4%.

^1^H NMR (400 MHz, CDCl_3_) δ: 7.29 (t, *J* = 8.4 Hz, 1H, CH arom.); 7.24 (d, *J* = 8.4 Hz, 2H, CH arom.); 7.14 (d, *J* = 8.4 Hz, 2H, CH arom.); 6.56 (d, *J* = 8.4 Hz, 2H, CH arom.); 6.56 (s, 1H, CH arom.); 6.50 (s, 1H, CH arom.); 3.83 (s, 6H, OCH_3_); 3.80 (s, 3H, OCH_3_); 3.79 (s, 3H, OCH_3_); 3.65 (s, 2H, NCH_2_Ar); 2.94–2.87 (m, 2H, CH_2_); 2.86–2.70 (m, 6H, CH_2_) ppm. ^13^C NMR (100 MHz, CDCl_3_) δ: 165.12 (C=O); 157.63 (C); 149.47 (C); 147.71 (C); 147.37 (C); 137.59 (C); 131.58 (CH arom.); 129.62 (CH arom.); 125.87 (C); 121.75 (CH arom.); 112.52 (C); 111.42 (CH arom.); 109.55 (CH arom.); 104.04 (CH arom.); 59.73 (CH_2_); 56.13 (OCH_3_); 55.96 (OCH_3_); 55.92 (OCH_3_); 55.39 (CH_2_); 50.90 (CH_2_); 33.19 (CH_2_); 28.28 (CH_2_) ppm. ESI-HRMS (*m/z*) calculated for [M + H]^+^ ion species C_28_H_32_NO_6_= 478.2224, found 478.2224.

**Hydrochloride:** mp 213–215 °C.

#### 4–(2-(6,7-Dimethoxy-3,4-dihydroisoquinolin-2(1H)-yl)ethyl)phenyl 2,3-dimethoxybenzoate (18)

Procedure **A** in an. CH_2_Cl_2_, starting from phenol **28** and 2,3-dimethoxybenzoic acid.

**Free base:** chromatographic eluent: CH_2_Cl_2_/MeOH/NH_4_OH 97:3:0.3. Yield: 65.5%.

^1^H NMR (400 MHz, CDCl_3_) δ: 7.47 (dd, *J* = 7.2, 2.2 Hz, 1H, CH arom.); 7.26 (d, *J* = 8.4 Hz, 2H, CH arom.); 7.15–7.07 (m, 4H, CH arom.); 6.57 (s, 1H, CH arom.); 6.51 (s, 1H, CH arom.); 3.92 (s, 3H, OCH_3_); 3.87 (s, 3H, OCH_3_); 3.81 (s, 3H, OCH_3_); 3.80 (s, 3H, OCH_3_); 3.66 (s, 2H, NCH_2_Ar); 2.96–2.89 (m, 2H, CH_2_); 2.88–2.68 (m, 6H, CH_2_) ppm. ^13^C NMR (100 MHz, CDCl_3_) δ: 164.74 (C=O); 153.76 (C); 149.71 (C); 149.27 (C); 147.66 (C); 147.33 (C); 137.78 (C); 129.72 (CH arom.); 125.93 (C); 125.43 (C); 123.95 (CH arom.); 122.54 (CH arom.); 121.68 (CH arom.); 116.44 (CH arom.); 111.38 (CH arom.); 109.50 (CH arom.); 61.60 (OCH_3_); 59.88 (CH_2_); 56.14 (OCH_3_); 55.95 (OCH_3_); 55.92 (OCH_3_); 55.51 (CH_2_); 50.97 (CH_2_); 33.27 (CH_2_); 28.42 (CH_2_) ppm. ESI-HRMS (*m/z*) calculated for [M + H]^+^ ion species C_28_H_32_NO_6_= 478.2224, found 478.2221.

**Hydrochloride:** mp 237–239 °C.

#### 4–(2-(6,7-Dimethoxy-3,4-dihydroisoquinolin-2(1H)-yl)ethyl)phenyl 1-naphthoate (19)

Procedure **A** in an. CH_2_Cl_2_, starting from phenol **28** and 1-naphthoic acid.

**Free base:** chromatographic eluent: CH_2_Cl_2_/MeOH/NH_4_OH 97:3:0.3. Yield: 77.5%.

^1^H NMR (400 MHz, CDCl_3_) δ: 9.00 (d, *J* = 8.0 Hz, 1H, CH arom.); 8.44 (d, *J* = 7.6 Hz, 1H, CH arom.); 8.07 (d, *J* = 8.0 Hz, 1H, CH arom.); 7.89 (d, *J* = 7.6 Hz, 1H, CH arom.); 7.61 (t, *J* = 7.6 Hz, 1H, CH arom.); 7.54 (t, *J* = 8.0 Hz, 2H, CH arom.); 7.31 (d, *J* = 8.4 Hz, 2H, CH arom.); 7.19 (d, *J* = 8.4 Hz, 2H, CH arom.); 6.59 (s, 1H, CH arom.); 6.53 (s, 1H, CH arom.); 3.82 (s, 6H, OCH_3_); 3.67 (s, 2H, NCH_2_Ar); 2.99–2.91 (m, 2H, CH_2_); 2.90–2.76 (m, 6H, CH_2_) ppm. ^13^C NMR (100 MHz, CDCl_3_) δ: 165.97 (C=O); 149.32 (C); 147.67 (C); 147.35 (C); 137.93 (C); 134.30 (CH arom.); 133.95 (C); 131.70 (C); 131.19 (CH arom.); 129.82 (CH arom.); 128.71 (CH arom.); 128.17 (CH arom.); 126.42 (CH arom.); 126.14 (C); 126.00 (C); 125.76 (CH arom.); 124.54 (CH arom.); 121.81 (CH arom.); 111.42 (CH arom.); 109.54 (CH arom.); 59.94 (CH_2_); 55.97 (OCH_3_); 55.94 (OCH_3_); 55.59 (CH_2_); 51.01 (CH_2_); 33.35 (CH_2_); 28.50 (CH_2_) ppm. ESI-HRMS (*m/z*) calculated for [M + H]^+^ ion species C_30_H_30_NO_4_= 468.2169, found 468.2174.

**Hydrochloride:** mp 227–230 °C.

#### 4–(2-(6,7-Dimethoxy-3,4-dihydroisoquinolin-2(1H)-yl)ethyl)phenyl 2-methoxy-1-naphthoate (20)

Procedure **A** in an CH_2_Cl_2_, starting from phenol **28** and 2-methoxy-1-naphthoic acid.

**Free base:** chromatographic eluent: CH_2_Cl_2_/MeOH/NH_4_OH 95:5:0.5. Yield: 73.3%.

^1^H NMR (400 MHz, CDCl_3_) δ: 7.92 (d, *J* = 8.8 Hz, 2H, CH arom); 7.80 (d, *J* = 7.6 Hz, 1H, CH arom.); 7.52 (t, *J* = 7.6 Hz, 1H, CH arom.); 7.38 (t, *J* = 7.6 Hz, 1H, CH arom.); 7.34–7.27 (m, 3H, CH arom.); 7.25 (d, *J* = 8.4 Hz, 2H, CH arom.); 6.58 (s, 1H, CH arom.); 6.52 (s, 1H, CH arom.); 4.00 (s, 3H, OCH_3_); 3.83 (s, 3H, OCH_3_); 3.82 (s, 3H, OCH_3_); 3.65 (s, 2H, NCH_2_Ar); 3.00–2.90 (m, 2H, CH_2_); 2.89–2.75 (m, 6H, CH_2_) ppm. ^13^C NMR (100 MHz, CDCl_3_) δ: 166.67 (C=O); 155.02 (C); 149.38 (C); 147.67 (C); 147.34 (C); 137.85 (C); 132.26 (CH arom.); 131.02 (C); 129.79 (CH arom.); 128.55 (C); 128.24 (CH arom.); 127.92 (CH arom.); 125.86 (C); 124.29 (CH arom.); 123.58 (CH arom.); 121.71 (CH arom.); 116.70 (C); 113.17 (CH arom.); 111.36 (CH arom.); 109.49 (CH arom.); 59.78 (CH_2_); 56.92 (OCH_3_); 55.95 (OCH_3_); 55.92 (OCH_3_); 55.43 (CH_2_); 50.92 (CH_2_); 33.24 (CH_2_); 28.32 (CH_2_) ppm. ESI-HRMS (*m/z*) calculated for [M + H]^+^ ion species C_31_H_32_NO_5_= 498.2275, found 498.2272.

**Hydrochloride:** mp 208–210 °C.

#### 4–(2-(6,7-Dimethoxy-3,4-dihydroisoquinolin-2(1H)-yl)ethyl)phenyl 2,3-dimethoxy-1-naphthoate (21)

Procedure **A** in an. CH_2_Cl_2_, starting from phenol **28** and 2,3-dimethoxy-1-naphthoic acid.

**Free base:** chromatographic eluent: CH_2_Cl_2_/MeOH/NH_4_OH 95:5:0.5. Yield: 26.5%.

^1^H NMR (400 MHz, CDCl_3_) δ: 7.89–7.83 (m 1H, CH arom.); 7.75–7.69 (m, 1H, CH arom.); 7.44–7.38 (m, 2H, CH arom.); 7.32 (d, *J* = 8.0 Hz, 2H, CH arom.); 7.26 (s, 1H, CH arom.); 7.25 (d, *J* = 8.0 Hz, 2H, CH arom.); 6.58 (s, 1H, CH arom.); 6.52 (s, 1H, CH arom.); 4.02 (s, 3H, OCH_3_); 3.98 (s, 3H, OCH_3_); 3.82 (s, 3H, OCH_3_); 3.81 (s, 3H, OCH_3_); 3.65 (s, 2H, NCH_2_Ar); 2.98–2.90 (m, 2H, CH_2_); 2.89–2.74 (m, 6H, CH_2_) ppm. ^13^C NMR (100 MHz, CDCl_3_) δ: 166.08 (C=O); 151.66 (C); 149.22 (C); 147.66 (C); 147.48 (C); 147.33 (C); 138.08 (C); 131.14 (C); 129.90 (CH arom.); 126.90 (CH arom.); 125.90 (CH arom.); 125.47 (C); 125.31 (CH arom.); 123.95 (CH arom.); 123.59 (C); 121.66 (CH arom.); 111.36 (CH arom.); 109.61 (CH arom.); 109.48 (CH arom.); 61.94 (OCH_3_); 59.85 (CH_2_); 55.95 (OCH_3_); 55.91 (OCH_3_); 55.87 (OCH_3_); 55.49 (CH_2_); 50.95 (CH_2_); 33.27 (CH_2_); 28.39 (CH_2_) ppm. ESI-HRMS (*m/z*) calculated for [M + H]^+^ ion species C_32_H_34_NO_6_= 528.2381, found 528.2384.

**Hydrochloride:** mp 183–185 °C.

#### 4–(2-(6,7-Dimethoxy-3,4-dihydroisoquinolin-2(1H)-yl)ethyl)phenyl nicotinate (22)

Procedure **A** in an. CH_3_CN, starting from phenol **28** and nicotinic acid.

**Free base:** chromatographic eluent: CH_2_Cl_2_/MeOH/NH_4_OH 93:7:0.3. Yield: 16.7%.

^1^H NMR (400 MHz, CDCl_3_) δ: 9.36 (s, 1H, CH arom.); 8.82 (d, *J* = 4.8, 1H, CH arom.); 8.41 (d, *J* = 8.0, 1H, CH arom.); 7.43 (dd, *J* = 8.0, 4.8 Hz, 1H, CH arom.); 7.28 (d, *J* = 8.4 Hz, 2H, CH arom.); 7.12 (d, *J* = 8.4 Hz, 2H, CH arom.); 6.57 (s, 1H, CH arom.); 6.51 (s, 1H, CH arom.); 3.81 (s, 3H, OCH_3_); 3.80 (s, 3H, OCH_3_); 3.65 (s, 2H, NCH_2_Ar); 2.97–2.90 (m, 2H, CH_2_); 2.88–2.74 (m, 6H, CH_2_) ppm. ^13^C NMR (100 MHz, CDCl_3_) δ: 164.00 (C=O); 153.97 (CH arom.); 151.37 (CH arom.); 148.87 (C); 147.73 (C); 147.39 (C); 138.14 (C); 137.57 (CH arom.); 129.85 (CH arom.); 125.84 (C); 125.65 (C); 123.45 (CH arom.); 121.46 (CH arom.); 111.40 (CH arom.); 109.52 (CH arom.); 59.64 (CH_2_); 55.96 (OCH_3_); 55.92 (OCH_3_); 55.40 (CH_2_); 50.88 (CH_2_); 33.16 (CH_2_); 28.26 (CH_2_) ppm. ESI-HRMS (*m/z*) calculated for [M + H]^+^ ion species C_25_H_27_N_2_O_4_= 419.1965, found 419.1958.

**Hydrochloride:** mp 98–100 °C.

#### 4–(2-(6,7-Dimethoxy-3,4-dihydroisoquinolin-2(1H)-yl)ethyl)phenyl 6-methoxynicotinate (23)

Procedure **B** in an. CH_3_CN, starting from phenol **28** and 6-methoxynicotinic acid.

**Free base:** chromatographic eluent: CH_2_Cl_2_/MeOH/NH_4_OH 95:5:0.5. Yield: 21.0%.

^1^H NMR (400 MHz, CDCl_3_) δ: 8.97 (d, *J* = 2.0 Hz, 1H, CH arom.); 8.25 (dd, *J* = 8.8, 2.0 Hz, 1H, CH arom.); 7.27 (d, *J* = 8.4 Hz, 2H, CH arom.); 7.10 (d, *J* = 8.4 Hz, 2H, CH arom.); 6.80 (d, *J* = 8.8 Hz, 1H, CH arom.); 6.58 (s, 1H, CH arom.); 6.51 (s, 1H, CH arom.); 4.00 (s, 3H, OCH_3_); 3.82 (s, 3H, OCH_3_); 3.81 (s, 3H, OCH_3_); 3.69 (s, 2H, NCH_2_Ar); 3.00–2.92 (m, 2H, CH_2_); 2.91–2.77 (m, 6H, CH_2_) ppm. ESI-HRMS (*m/z*) calculated for [M + H]^+^ ion species C_26_H_29_N_2_O_5_= 449.2071, found 449.2063.

**Hydrochloride:** mp 254–256 °C.

#### 4–(2-(6,7-Dimethoxy-3,4-dihydroisoquinolin-2(1H)-yl)ethyl)phenyl pyrazine-2-carboxylate (24)

Procedure **B** in CHCl_3_ (free of ethanol), starting from phenol **28** and pyrazine-2-carboxylic acid.

**Free base:** chromatographic eluent: CH_2_Cl_2_/MeOH/NH_4_OH 97:3:0.3. Yield: 21.8%.

^1^H NMR (400 MHz, CDCl_3_) δ: 9.40 (d, *J* = 1.2 Hz, 1H, CH arom.); 8.78 (d, *J* = 2.4 Hz, 1H, CH arom.); 8.75 (dd, *J* = 2.4, 1.2 Hz, 1H, CH arom.); 7.26 (d, *J* = 8.8 Hz, 2H, CH arom.); 7.14 (d, *J* = 8.8 Hz, 2H, CH arom.); 6.55 (s, 1H, CH arom.); 6.49 (s, 1H, CH arom.); 3.79 (s, 3H, OCH_3_); 3.78 (s, 3H, OCH_3_); 3.61 (s, 2H, NCH_2_Ar); 2.92–2.85 (m, 2H, CH_2_); 2.83–2.72 (m, 6H, CH_2_) ppm. ^13^C NMR (100 MHz, CDCl_3_) δ: 162.66 (C=O); 148.80 (C); 148.16 (CH arom.); 147.71 (C); 147.36 (C); 146.77 (CH arom.); 144.67 (CH arom.); 142.96 (C); 138.18 (C); 129.93 (CH arom.); 125.62 (C); 125.36 (C); 121.40 (CH arom.); 111.29 (CH arom.); 109.40 (CH arom.); 59.38 (CH_2_); 55.93 (OCH_3_); 55.89 (OCH_3_); 55.17 (CH_2_); 50.73 (CH_2_); 32.97 (CH_2_); 27.97 (CH_2_) ppm. ESI-HRMS (*m/z*) calculated for [M + H]^+^ ion species C_24_H_26_N_3_O_4_= 420.1918, found 420.1911.

**Hydrochloride:** mp 96–99 °C.

#### 4–(2-(6,7-Dimethoxy-3,4-dihydroisoquinolin-2(1H)-yl)ethyl)phenyl 6-methoxypyrazine-2-carboxylate (25)

Procedure **B** in an. CH_3_CN, starting from phenol **28** and 6-methoxypyrazine-2-carboxylic acid.

**Free base:** chromatographic eluent: CH_2_Cl_2_/MeOH/NH_4_OH 93:7:0.3. Yield: 75.6%.

^1^H NMR (400 MHz, CDCl_3_) δ: 8.95 (s, 1H, CH arom.); 8.41 (s, 1H, CH arom.); 7.28 (d, *J* = 8.4 Hz, 2H, CH arom.); 7.14 (d, *J* = 8.4 Hz, 2H, CH arom.); 6.57 (s, 1H, CH arom.); 6.50 (s, 1H, CH arom.); 4.05 (s, 3H, OCH_3_); 3.81 (s, 3H, OCH_3_); 3.80 (s, 3H, OCH_3_); 3.63 (s, 2H, NCH_2_Ar); 2.95–2.90 (m, 2H, CH_2_); 2.89–2.73 (m, 6H, CH_2_) ppm. ^13^C NMR (100 MHz, CDCl_3_) δ: 162.79 (C=O); 159.99 (C); 148.90 (C); 147.62 (C); 147.29 (C); 140.08 (CH arom.); 139.08 (C); 138.38 (CH arom.); 138.33 (C); 129.82 (CH arom.); 126.09 (C); 125.96 (C); 121.34 (CH arom.); 111.37 (CH arom.); 109.49 (CH arom.); 59.80 (CH_2_); 55.93 (OCH_3_); 55.90 (OCH_3_); 55.52 (CH_2_); 54.13 (OCH_3_); 50.94 (CH_2_); 33.27 (CH_2_); 28.44 (CH_2_) ppm. ESI-HRMS (*m/z*) calculated for [M + H]^+^ ion species C_25_H_28_N_3_O_5_= 450.2024, found 450.2029.

**Hydrochloride:** mp 214–216 °C.

#### 4–(2-(6,7-Dimethoxy-3,4-dihydroisoquinolin-2(1H)-yl)ethyl)phenyl 6-methoxyquinoline-4-carboxylate (26)

Procedure **B** in CHCl_3_ (free of ethanol), starting from phenol **28** and 6-methoxyquinoline-4-carboxylic acid.

**Free base:** chromatographic eluent: CH_2_Cl_2_/MeOH/NH_4_OH 97:3:0.3. Yield: 32.5%.

^1^H NMR (400 MHz, CDCl_3_) δ: 8.93 (d, *J* = 4.8 Hz, 1H, CH arom.); 8.33 (d, *J* = 2.4 Hz, 1H, CH arom.); 8.19 (d, *J* = 4.8 Hz, 1H, CH arom.); 8.09 (d, *J* = 9.2 Hz, 1H, CH arom.); 7.44 (dd, *J* = 9.2, 2.4 Hz, 1H, CH arom.); 7.36 (d, *J* = 8.4 Hz, 2H, CH arom.); 7.20 (d, *J* = 8.4 Hz, 2H, CH arom.); 6.61 (s, 1H, CH arom.); 6.55 (s, 1H, CH arom.); 3.93 (s, 3H, OCH_3_); 3.85 (s, 3H, OCH_3_); 3.84 (s, 3H, OCH_3_); 3.71 (s, 2H, NCH_2_Ar); 3.02–2.97 (m, 2H, CH_2_); 2.92–2.80 (m, 6H, CH_2_) ppm. ^13^C NMR (100 MHz, CDCl_3_) δ: 165.03 (C=O); 159.57 (C); 148.91 (C); 147.79 (C); 147.42 (C); 146.93 (CH arom.); 145.80 (C); 137.98 (C); 131.57 (CH arom.); 131.20 (C); 130.04 (CH arom.); 127.04 (C); 125.50 (C); 123.37 (CH arom.); 123.15 (CH arom.); 121.68 (CH arom.); 111.26 (CH arom.); 109.37 (CH arom.); 102.97 (CH arom.); 59.36 (CH_2_); 55.95 (OCH_3_); 55.91 (OCH_3_); 55.65 (OCH_3_); 55.12 (CH_2_); 50.74 (CH_2_); 32.91 (CH_2_); 27.90 (CH_2_) ppm. ESI-HRMS (*m/z*) calculated for [M + H]^+^ ion species C_30_H_31_N_2_O_5_= 499.2228, found 499.2226.

**Hydrochloride:** mp 203–206 °C.

#### 2-Methoxy-1-naphthoic acid (29)^42^

To a stirred solution of CuBr_2_ (0.04 mmol) and 2-methoxy-1-naphthaldehyde (0.80 mmol) in 3.0 ml of an CH_3_CN was added 70% *t*-BuOOH in water (1.60 mmol). When all the aldehyde had been consumed, the solvent was removed under reduced pressure. The reaction mixture was treated with a saturated solution of NaHCO_3_ and extracted with ethyl acetate. The aqueous layer was acidified using 2 M HCl and then extracted with ethyl acetate. The organic phase was dried with Na_2_SO_4_, then the solvent was removed under reduced pressure. 2-methoxy-1-naphthoic acid was obtained pure as a pale yellow solid.

Yield: 67.6%. ^1^H NMR (400 MHz, CDCl_3_) δ: 8.38 (d, *J* = 8.4 Hz, 1H, CH arom.); 7.95 (d, *J* = 9.2 Hz, 1H, CH arom.); 7.78 (d, *J* = 8.4 Hz, 1H, CH arom.); 7.55 (t, *J* = 8.4 Hz, 1H, CH arom.); 7.39 (t, *J* = 8.4 Hz, 1H, CH arom.); 7.30 (d, *J* = 9.2 Hz, 1H, CH arom.); 4.05 (s, 3H, OCH_3_).

#### 2,3-Dimethoxy-1-naphthoic acid (30)^43^

To a solution of 2,3-dimethoxy-1-naphthaldehyde (0.23 mmol) in 3.0 ml of acetone was added Na_2_CO_3_ (0.23 mmol) in 1.0 ml of water. Then KMnO_4_ (0.23 mmol) was added portion wise. The reaction mixture was stirred at rt for 18 h then filtered. The filtrate was concentrated and extracted with ethyl acetate. The aqueous layer was acidified to pH 1 with 1 M HCl then extracted with ethyl acetate. After drying with Na_2_SO_4_, the solvent was removed under reduced pressure and 2,3-dimethoxy-1-naphthoic acid was obtained as a pale yellow solid.

Yield: 93.3%. ^1^H NMR (400 MHz, CDCl_3_) δ: 10.40 (s, 1H, OH); 7.89 (d, *J* = 7.6 Hz, 1H, CH arom.); 7.63 (d, *J* = 7.6 Hz, 1H, CH arom.); 7.35 (t, *J* = 7.6 Hz, 1H, CH arom.); 7.30 (t, *J* = 7.6 Hz, 1H, CH arom.); 7.14 (s, 1H, CH arom.); 3.92 (s, 3H, OCH_3_); 3.89 (s, 3H, OCH_3_).

#### 6-Methoxyquinoline-4-carboxylic acid (31)^44^

To a solution of quinine sulphate (0.90 mmol) in 12 ml of 10% sulphuric acid, MnO_2_ (1.70 mmol) was added. The mixture was raised to the boiling point, then CrO_3_ (14.30 mmol) dissolved in 3 ml of water was added dropwise during an hour. The refluxing was continued for 3 h, then 126 ml of water and 28 ml of 15 N ammonia were added. The reaction mixture was stirred at 100 °C for 18 h, then filtered with Celite. The residue was washed several times with hot 15 N ammonia solution. The combined filtrates were concentrated under reduced pressure, then acidified with acetic acid and filtered, to obtain 6-methoxyquinoline-4-carboxylic acid as pure yellow solid.

Yield: 89.4%. ^1^H NMR (400 MHz, DMSO-d_6_) δ: 8.83 (d, *J* = 2.0 Hz, 1H, CH arom.); 8.16 (s, 1H, CH arom.); 7.99 (d, *J* = 9.2 Hz, 1H, CH arom.); 7.88 (d, *J* = 2.0 Hz, 1H, CH arom.); 7.46 (d, *J* = 9.2 Hz, 1H, CH arom.); 3.88 (s, 3H, OCH_3_).

### Biological assays

#### Cell lines and cultures

MDCK-MDR1, MDCK-MRP1 and MDCK-BCRP cells are a gift of Prof. P. Borst, NKI-AVL Institute, Amsterdam, The Netherlands. Caco-2 cells were a gift of Dr. Aldo Cavallini and Dr. Caterina Messa from the Laboratory of Biochemistry, National Institute for Digestive Diseases, “S. de Bellis”, Bari (Italy). HT29 and A549 were purchased from ATCC (Manassas, VA). The doxorubicin-resistant sublines, HT29/DX and A549/DX cells were generated by stepwise selection in medium with increasing concentrations of doxorubicin^45^ and maintained in culture medium with 100 and 50 nM doxorubicin, respectively. MDCK and Caco-2 cells were grown in DMEM high glucose, HT29 and HT29/DX in RPMI-1640, A549 and A549/DX in HAM-F12 medium, all supplemented with 10% foetal bovine serum, 2 mM glutamine, 100 U/mL penicillin, 100 µg/mL streptomycin, in a humidified incubator at 37 °C with a 5% CO2 atmosphere.

#### Drugs and chemicals

Cell culture reagents were purchased from Celbio s.r.l. (Milano, Italy). CulturePlate 96/wells plates were purchased from PerkinElmer Life Science; Calcein-AM, bisBenzimide H 33342 trihydrochloride were obtained from Sigma-Aldrich (Milan, Italy).

#### Immunoblotting analysis of P-gp, MRP1 and BCRP expression in MDCK, MDCK-MDR1, MDCK-MRP1 and MDCK-BCRP cells

All MDCK cells were rinsed with lysis buffer (50 mM Tris-HCl, 1 mM EDTA, 1 mM EGTA, 150 mM NaCl, 1% v/v Triton-X100; pH 7.4), supplemented with the protease inhibitor cocktail III (Cabiochem, La Jolla, CA), sonicated and clarified at 13000 × g, for 10 min at 4 °C. Protein extracts (20 µg) were subjected to SDS-PAGE and probed with the following antibodies: anti-P-glycoprotein (P-gp; 1:250, rabbit polyclonal, #sc-8313, Santa Cruz Biotechnology Inc., Santa Cruz, CA), anti-multidrug resistant protein 1 (MRP1; 1:500, mouse clone MRPm5, Abcam, Cambridge, UK), anti-breast cancer resistance protein (BCRP; 1:500, mouse clone BXP-21, Santa Cruz Biotechnology Inc.), anti-β-tubulin (1:1000, mouse clone D10, Santa Cruz Biotechnology Inc.), followed by the horseradish peroxidase-conjugated secondary antibodies (Bio-Rad). The membranes were washed with Tris-buffered saline (TBS)/Tween 0.01% v/v. Proteins were detected by enhanced chemiluminescence (Bio-Rad Laboratories).

#### Calcein-AM experiments

These experiments were carried out as previously described by Teodori et al. with minor modifications^34^. Each cell line (30 000 cells per well) was seeded into black CulturePlate 96/wells plate with 100 µL medium and allowed to become confluent overnight. 100 µL of test compounds were solubilised in culture medium and added to monolayers, with final concentrations ranging from 1 nM to 100 µM. 96/Wells plate was incubated for 30 min in a humidified atmosphere 5% CO_2_ at 37 °C. Calcein-AM was added in 100 µL of Phosphate Buffered Saline (PBS) to yield a final concentration of 2.5 µM and plate was incubated for 30 min in a humidified atmosphere 5% CO_2_ at 37 °C. Each well was washed 3 times with ice cold PBS. Saline buffer was added to each well and the plate was read with Victor3 (PerkinElmer) at excitation and emission wavelengths of 485 and 535 nm, respectively. In these experimental conditions, Calcein cell accumulation in the absence and in the presence of tested compounds was evaluated and fluorescence basal level was estimated with untreated cells. In treated wells the increase of fluorescence with respect to basal level was measured. EC_50_ values were determined by fitting the fluorescence increase percentage versus log[dose].

#### Hoechst 33342 experiment

These experiments were carried out as described by Teodori et al.^34^. Each cell line (30 000 cells per well) was seeded into black CulturePlate 96/wells plate with 100 µL medium and allowed to become confluent overnight. 100 µL of test compounds were solubilised in culture medium and added to monolayers, with final concentrations ranging from 1 to 100 µM. 96/Wells plate was incubated for 30 min in a humidified atmosphere 5% CO_2_ at 37 °C. Hoechst 33342 was added in 100 µl of phosphate-buffered saline (PBS) to yield a final concentration of 8 µM and plate was incubated for 30 min in a humidified atmosphere 5% CO_2_ at 37 °C. The supernatants were drained, and the cells were fixed for 20 min under light protection using 100 µL per well of a 4% PFA solution. Each well was washed three times with ice cold PBS. Saline buffer was added to each well and the plate was read with Victor3 (PerkinElmer) at excitation and emission wavelengths of 340/35 nm and 485/20 nm, respectively. In these experimental conditions, Hoechst 33342 accumulation in the absence and in the presence of tested compounds was evaluated and fluorescence basal level was estimated with untreated cells. In treated wells, the increase of fluorescence with respect to basal level was measured. EC_50_ values were determined by fitting the fluorescence increase percentage versus log[dose].

#### ATPlite assay

The MDCK-MDR1 cells were seeded into 96-well microplate in 100 µL of complete medium at a density 20,000 cells per well. The plate was incubated overnight (O/N) in a humidified atmosphere 5% CO_2_ at 37 °C. The medium was removed and 100 µL of complete medium either alone or containing different concentrations (ranging from 1 to 100 µM) of test compounds was added. The plate was incubated for 2 h in a humidified 5% CO_2_ atmosphere at 37 °C. 50 µL of mammalian cell lysis solution was added to all wells and the plate shacked for five minutes in an orbital shaker. 50 µL of substrate solution was added to all wells and the plate shacked for five minutes in an orbital shaker. The plate was dark adapted for ten minutes and the luminescence was measured.

### Permeability experiments

#### Preparation of caco-2 monolayer

Caco-2 cells were seeded onto a Millicell® assay system (Millipore), where a cell monolayer is set in between a filter cell and a receiver plate, at a density of 10 000 cells/well^46^. The culture medium was replaced every 48 h and the cells kept for 21 days in culture. The Trans Epithelial Electrical Resistance (TEER) of the monolayers was measured daily, before and after the experiment, using an epithelial volt-ohmmeter (Millicell®-ERS). Generally, TEER values greater than 1000 Ω for a 21 days culture, are considered optimal.

#### Drug transport experiment

After 21 days of Caco-2 cell growth, the medium was removed from filter wells and from the receiver plate, which were filled with fresh HBSS buffer (Invitrogen). This procedure was repeated twice, and the plates were incubated at 37 °C for 30 min. After incubation time, the HBSS buffer was removed and drug solutions and reference compounds were added to the filter well at the concentration of 100 µM, while fresh HBSS was added to the receiver plate. The plates were incubated at 37 °C for 120 min. Afterwards, samples were removed from the apical (filter well) and basolateral (receiver plate) side of the monolayer to measure the permeability.

The apparent permeability (*P*_app_), in units of nm/second, was calculated using the following equation:

$$P_{\text{app}}=\left(\frac{V_{A}}{\text{Area}\times \text{time}}\right)\times \left(\frac{{\left[\text{drug}\right]}_{\text{acceptor}}}{{\left[\text{drug}\right]}_{\text{initial}}}\right)$$

- VA = the volume (in mL) in the acceptor well;
- Area = the surface area of the membrane (0.11 cm^2^ of the well);
- time = the total transport time in seconds (7200s);
- [drug]acceptor = the concentration of the drug measured by U.V. spectroscopy;
- [drug]initial = the initial drug concentration (1 × 10^−4 ^M) in the apical or basolateral wells.

### Co-administration assay

The co-administration assay with doxorubicin was performed in MDCK-MDR1, HT29, HT29/DX, A549 and A549/DX cells at 48 h. On day 1, 10,000 cells/well were seeded into 96-well plates in a volume of 100 µL of fresh medium. On day 2, the tested drug was added alone to the cells at different concentrations (ranging from 40 nM to 10 µM). On day 3, the medium was removed and the drug at the same concentrations was added alone and in co-administration with 10 µM doxorubicin to the cells. After the established incubation time with the tested drug, MTT (0.5 mg/mL) was added to each well, and after 3 − 4 h incubation at 37 °C, the supernatant was removed. The formazan crystals were solubilised using 100 µL of DMSO/EtOH (1:1), and the absorbance values at 570 and 630 nm were determined on the microplate reader HTX Synergy (Bio-Tek Instruments).

### Statistical analysis

All data in the text and figures are provided as means ± SEM. The results were analysed by a Student’s *t*-test and one-way ANOVA test, using Graph-Pad Prism (Graph-Pad software, San Diego, CA). *p* < 0.05 was considered significant.

### Stability tests

#### Chemicals

Acetonitrile (Chromasolv), formic acid and ammonium formate (MS grade), NaCl, KCl, Na_2_HPO_4_ 2H_2_O, KH_2_PO_4_ (Reagent grade), verapamil hydrochloride (analytical standard, used as internal standard) and ketoprofen (analytical standard) were purchased by Sigma-Aldrich (Milan, Italy). Ketoprofen ethyl ester (KEE) were obtained by Fisher’s reaction from ketoprofen and ethanol.

MilliQ water 18 MΩ cm^−1^ was obtained from Millipore’s Simplicity system (Milan, Italy).

Phosphate-buffered solution was prepared by adding 8.01 g L^−1^ of NaCl, 0.2 g L^−1^ of KCl, 1.78 g L^−1^ of Na_2_HPO_4_ 2H_2_O and 0.27 g L^−1^ of KH_2_PO_4_. Human plasma was collected from healthy male volunteer and kept at −80 °C until use.

#### Preparation of samples

Each sample was prepared adding 10 µL of working solution 1 to 100 µL of tested matrix (PBS or human plasma) in micro centrifuge tubes. The obtained solutions correspond to 1 µM of analyte.

Each set of samples was incubated in triplicate at four different times, 0, 30, 60 and 120 min at 37 °C. Therefore, the degradation profile of each analyte was represented by a batch of 12 samples (4 incubation times × 3 replicates). After the incubation, the samples were added with 300 µL of ISTD solution and centrifuged (room temperature for 5 min at 10,000 rpm). The supernatants were transferred in auto sampler vials and dried under a gentle stream of nitrogen.

The dried samples were dissolved in 1.0 ml of 10 mM of formic acid in mQ water: acetonitrile 80:20 solution. The obtained sample solutions were analysed by LC-MS/MS methods described in Supplementary material.

## Results and discussion

### Chemistry

**Scheme 1.**
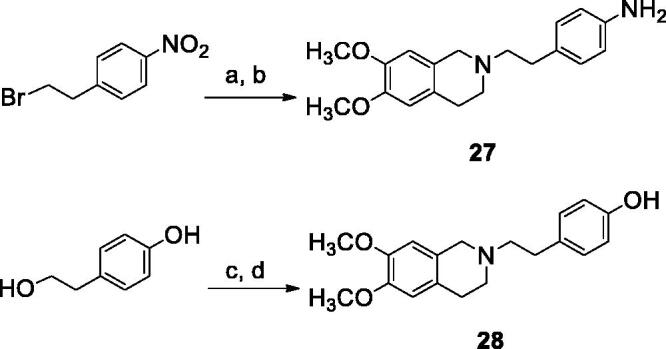
Reagents and conditions: (a) 6,7-dimethoxy-1,2,3,4-tetrahydroisoquinoline HCl, K_2_CO_3_, an. CH_3_CN; (b) H_2_, Pd/C, EtOH; (c) SOCl_2_, ethanol-free CHCl_3_, an. Et_3_N; (d) 6,7-dimethoxy-1,2,3,4-tetrahydroisoquinoline HCl, K_2_CO_3_, an. CH_3_CN.

**Scheme 2.**
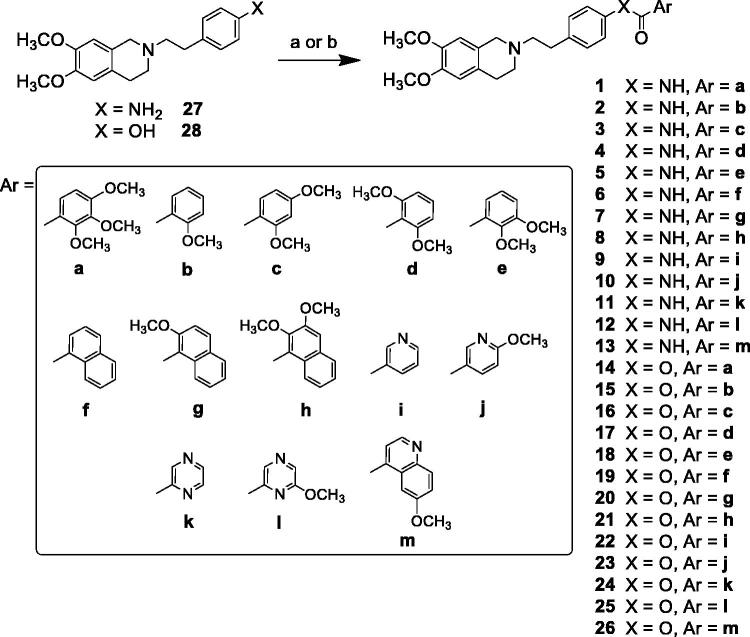
Reagents and conditions: (a) ArCOCl, ethanol-free CHCl_3_ or an. CH_3_CN; (b) ArCOOH, EDCI, DMAP, an. CH_2_Cl_2_ or CH_3_CN.

**Scheme 3.**
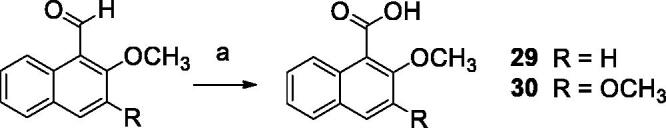
Reagents and conditions: (a) R = H, CuBr_2_, *t*-BuOOH, an. CH_3_CN; R = OCH_3_, Na_2_CO_3_, KMnO_4_, acetone.

**Scheme 4.**
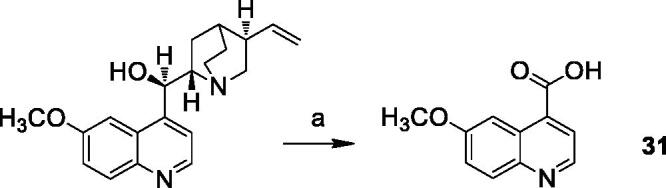
Reagents and conditions: (a) H_2_SO_4_ (water sol. 10%), MnO_2_, CrO_3_, NH_3_ (15 N).

The reaction pathways used to synthesise derivatives **1–26** are reported in Schemes 1–4. The key intermediates needed to achieve amides and esters were the aniline **27** and the phenol **28**, respectively, which were prepared (Scheme 1) as reported previously^34^: aniline **27** was obtained by alkylation of 6,7-dimethoxy-1,2,3,4-tetrahydroisoquinoline hydrochloride with 1–(2-bromoethyl)−4-nitrobenzene followed by catalytic reduction of the nitro group; phenol **28** was synthesised starting from the commercially available 4-(hydroxyethyl)phenol, transformed in the corresponding chloroethyl derivative by treatment with SOCl_2_ in ethanol-free CHCl_3_ and anhydrous triethylamine and then reacted with 6,7-dimethoxy-1,2,3,4-tetrahydroisoquinoline hydrochloride in anhydrous CH_3_CN in the presence of K_2_CO_3_. Amides **1–13** and esters **14–26** were obtained by reaction of **27** and **28**, respectively, with the proper carboxylic acid using EDC hydrochloride and DMAP in anhydrous CH_2_Cl_2_ or CH_3_CN, or with the acyl chloride obtained by treatment of the suitable acid with SOCl_2_ in ethanol-free CHCl_3_ or anhydrous CH_3_CN (Scheme 2, for details see Experimental Section). Most of the carboxylic acids were commercially available. 2-Methoxy-1-naphthoic acid **29** was synthetised starting from the corresponding aldehyde, following the general procedure described by Das^42^ (Scheme 3), while 2,3-dimethoxy-1-naphthoic acid **30** was obtained from the corresponding aldehyde as described by Ohnmacht^43^ (Scheme 3). 6-Methoxyquinoline-4-carboxylic acid **31** was synthesised following the procedure described by Kowanko^44^ (Scheme 4).

### Biological activity: characterisation of P-gp interacting profile and ABC transporters selectivity

The interaction potency between the designed compounds and P-gp was evaluated by measuring the transport inhibition of the pro-fluorescent probe Calcein-AM, that is a P-gp substrate, in a cell line overexpressing P-gp (MDCK-MDR1)^47^. The P-gp interacting profile was further investigated with two other assays: the Apparent Permeability (*P*_app_) determination (BA/AB) in Caco-2 cell monolayer^48^^,^^49^, and the ATP cell depletion in the MDCK-MDR1 cell line^50^. Taking into account the results of the combined tests, an interacting compound can be classified as substrate or inhibitor. *P*_app_ determination measures the ratio between two fluxes: from the basolateral to apical compartments (BA, representative of passive diffusion) and from the apical to basolateral compartments (AB, representative of active transport). A (BA/AB) value <2 suggests that the compound can be considered an inhibitor. In the same manner, if (BA/AB) >2, the compound should probably be classified as a substrate^51^. The other assay detects the consumption of ATP elicited by the transport mediated by the pump; in general, a substrate induces ATP cell depletion being transported by the pump (unambiguous substrate, category I), while a P-gp inhibitor does not induce ATP consumption. There is also a third substrate category (known as category IIB3) displaying a *P*_app_ value >2 but not inducing an ATP cell depletion^47^. The selectivity of ligands **1–26** was evaluated by detecting their activity towards the other ABC transporters MRP1 and BCRP, by measuring the inhibition of the efflux of Calcein-AM (i.e., also a MRP1 substrate) in cells overexpressing MRP1 (MDCK-MRP1 cells), and the inhibition of the efflux of the fluorescent probe Hoechst 33342 (i.e. a BCRP substrate) in cells overexpressing BCRP (MDCK-BCRP cells), respectively.

Expression levels of P-gp, MRP1 and BCRP were periodically analysed by immunoblotting analysis in MDCK-MDR1, MDCK-MRP1 and MDCK-BCRP cells respectively, as described in the Experimental Section. Tubulin was used as control of equal protein loading. A representative western blot analysis is reported in the Supplemental Material.

The results of the three inhibition assays on compounds **1–26** are reported in Table 1 together with those on tariquidar and elacridar used as reference compounds. The already described 3,4,5-trimethoxyphenyl derivatives **I** (amide) and **II** (ester)^34^ have been added for comparison.

**Table 1.** MDR-reversing activity, P-gp interaction profile and calculated human plasma half-life of compounds **1–26.**


			(EC_50_) µM^a^					
Compound	X	Ar	P-gp	MRP1	BCRP	ATP cell depletion	*P*_app_^b^	*t*_1/2_ (min)^c^
**1**	NH	a	0.53 ± 0.10	NA	21.2 ± 4.2	No	7.0	≥240
**2**	NH	b	0.44 ± 0.082	NA	40%	No	5.9	≥240
**3**	NH	c	0.0405 ± 0.008	31%^d^	1.0 ± 0.2	No	7.4	≥240
**4**	NH	d	7.20 ± 1.39	NA	NA	No	5.5	≥240
**5**	NH	e	0.26 ± 0.050	NA	41%^d^	No	5.0	≥240
**6**	NH	f	0.67 ± 0.12	NA	NA	No	3.6	≥240
**7**	NH	g	0.12 ± 0.022	NA	NA	No	11.8	≥240
**8**	NH	h	0.0478 ± 0.0093	47%^d^	NA	No	5.5	≥240
**9**	NH	i	6.40 ± 1.26	NA	NA	No	7.3	≥240
**10**	NH	j	2.30 ± 0.44	NA	NA	No	7.5	≥240
**11**	NH	k	1.90 ± 0.32	NA	NA	No	4.9	≥240
**12**	NH	l	0.87 ± 0.17	NA	NA	No	6.0	≥240
**13**	NH	m	0.66 ± 0.13	NA	NA	No	6.5	≥240
**I**^e^	NH	n	1.04 ± 0.20	NA	NA	No	5.1	≥240
**14**	O	a	0.36 ± 0.069	69.7 ± 13.9	15.1 ± 2.89	No	9.0	≥240
**15**	O	b	0.73 ± 0.14	NA	47%^d^	No	5.4	63 ± 14
**16**	O	c	0.10 ± 0.02	87.0 ± 17.0	11.0 ± 2.0	No	7.1	88 ± 22
**17**	O	d	0.15 ± 0.03	NA	NA	No	6.2	≥240
**18**	O	e	0.34 ± 0.07	NA	44%^d^	No	4.1	54 ± 9
**19**	O	f	0.84 ± 0.16	NA	35%^d^	No	5.1	≥240
**20**	O	g	0.11 ± 0.02	NA	7.3 ± 1.4	No	4.8	≥240
**21**	O	h	0.10 ± 0.02	NA	43%^d^	No	5.4	≥240
**22**	O	i	29.40 ± 4.98	NA	NA	No	5.0	≥240
**23**	O	j	9.24 ± 1.81	NA	NA	No	5.6	≥240
**24**	O	k	1.90 ± 0.32	NA	NA	No	4.9	42 ± 20
**25**	O	l	0.22 ± 0.04	NA	NA	No	4.7	6 ± 1
**26**	O	m	1.40 ± 0.26	NA	NA	No	8.7	124 ± 27
**II**^e^	O	n	0.93 ± 0.18	NA	17.0 ± 3.2	No	5.5	≥240
**tariq**			0.044 ± 0.001	ND	0.010 ± 0.005	Yes^f^	>20	ND
**elacr**			0.014 ± 0.003	NA	10.0 ± 2.0	Yes^g^	>20	ND

^a^ Values are the mean ± SEM of two independent experiments, with samples in triplicate.

^b^ Apparent permeability estimation: values are from two independent experiments, with samples in duplicate.

^c^ The half-life (*t*_1/2_) values were referred to the human plasma matrix.

^d^ Percentage of the effect at a concentration of 100 μM.

^e^ See reference^34^.

^f^ 30% at a concentration of 50 μM.

^g^ 25% at a concentration of 10 μM. NA: not active; ND: not determined.

As shown in Table 1, all the compounds were active on P-gp. Indeed, most of them showed a high P-gp potency, with EC_50_ values below 1 µM, reaching also the nanomolar range, as in the case of compounds **3** and **8** (EC_50_= 40.5 nM and 47.8 nM, respectively). Only eight among the tested derivatives exhibited a lower P-gp activity, with EC_50_ values higher than 1 µM.

Our research started with the synthesis of 2,3,4-trimethoxyphenyl derivatives **1** (amide) and **14** (ester), which are isomers of the reference compounds **I** and **II**^34^, reported in Table 1; remarkably, both the 2,3,4-functionalized isosteres, bearing a substituent in ortho position, were more potent than the reference compounds. This result prompted us to synthesise a series of phenyl analogues carrying an ortho methoxy substituent, also combined with a second methoxy residue in different positions (amides **2–5** and esters **15–18**). Moreover, since in a preceding work we had verified the positive effect of inserting a naphthalene ring^52^, we also obtained compounds **7**, **8**, **20** and **21** and the non-substituted analogues **6** and **19** for comparison purposes.

As general trend, substituted benzene and naphthalene derivatives displayed activities in the submicromolar or nanomolar range (EC_50_ ranging from 40.5 to 0.67 µM for amides and from 0.10 to 0.84 µM for esters), with the exception of amide **4**, carrying the 2,6-dimethoxy phenyl moiety, which showed an EC_50_ value of 7.20 µM. In this set of derivatives, it was not easy to define a relationship between potency values and structural characteristics: some compounds with interesting EC_50_ values could be found both among the amides and the esters, but the nature of the aryl moieties differently influenced the two groups of isosteres, without a definite trend. However, biological data confirmed that the presence of an ortho methoxy substituent is favourable for the activity. The most interesting result was the discovery of two amides endowed with an outstanding potency in the nanomolar range: the 2,4-dimethoxy phenyl derivative **3** (EC_50_ = 0.0405 µM) and the 2,3-dimethoxy naphthalene derivative **8** (EC_50_ = 0.0478 µM).

Compounds **9–13** and **22–26** instead carry a nitrogen-containing heterocycle: amides **9** and **11**, and esters **22** and **24** are non-substituted pyridine or pyrazine derivatives, while **10**, **12**, **23** and **25** are the corresponding methoxylated analogues. The quinoline isosteres **13** and **26** were obtained only as methoxy-substituted molecules. These hetero aromatic derivatives displayed lower potency: only amides **12** and **13**, and ester **25** showed a moderate/good activity (EC_50_ = 0.87, 0.66 and 0.22 µM, respectively). In the case of heterocycles, it was possible to verify that the presence of the OCH_3_ substituent improved the inhibiting activity on P-gp (compare amides **9** with **10**, or **11** with **12**, and the corresponding esters **22** with **23**, or **24** with **25**), and that the presence of an unsubstituted pyridine ring was detrimental for the activity. As regards the methoxy-quinoline derivatives, amide **13** was more potent than the corresponding ester **26**.

With respect to the reference compounds tariquidar (EC_50_ = 0.044 µM) and elacridar (EC_50_ = 0.014 µM), compounds **3** and **8** showed a comparable activity on P-gp, endowed with an EC_50_ value in the nanomolar range (as above reported) and a P-gp maximum inhibition (90–95%) at 10 µM; the other compounds were less potent at a different extent, but more selective since almost all of them were inactive towards both MRP1 and BCRP. As regard BCRP, although previous SAR studies suggested that the 6,7-dimethoxy-2-phenethyl-1,2,3,4-tetrahydroisoquinoline nucleus is a requirement for inhibiting both P-gp and BCRP, only six of these derivatives showed a moderate activity versus BCRP (EC_50_ ranging from 1.0 to 21.2 µM), belonging to the amide and the ester series. Interestingly, the potent P-gp inhibitor **3** (2,4-dimethoxyphenyl amide) was also the most active in BCRP inhibition (EC_50_ = 1.0 µM). Regarding MRP1 inhibition, all the compounds could be considered inactive: only in the case of two ester derivatives, **14** and **16**, an EC_50_ (69.7 and 87.0 µM) could be defined. It is noteworthy that these two compounds, the 2,3,4-trimethoxyphenyl ester **14** and the 2,4-dimethoxyphenyl ester **16**, were active also on BCRP and highly potent *vs* P-gp, showing a profile endowed with a low selectivity.

Finally, as regard the P-gp interacting mechanism, all the compounds behaved as not transported substrates (category IIB3), since they had a BA/AB ratio >2 and were not able to induce ATP cell depletion^47^.

On the bases of the obtained results, some very potent compounds were present both among the amide and the ester derivatives; however, the most interesting derivatives (**3** and **8**) belonged to amide series. These two compounds showed different selectivity profiles, since compound **3** was active also on BCRP, while compound **8** was not.

### Co-administration assay

Compounds **3** and **8**, endowing with the best P-gp activity profile in MDCK-MDR1, were tested alone and in co-administration with the antineoplastic drug, doxorubicin, that, as P-gp substrate, is usually inactive in tumours overexpressing the pump. A preliminary study was performed testing the two ligands **3** and **8** alone and in co-administration with 100 nM and 1 µM doxorubicin, without observing a significant reverting effect (data not shown). Therefore, we performed the same experiment using doxorubicin concentration of 10 µM.

**Figure 2.**
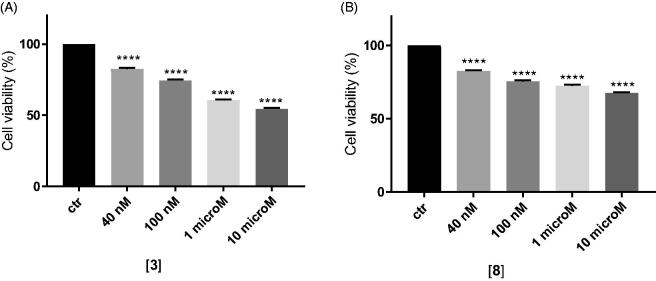
*In vitro* cell growth experiments performed on MDCK-MDR1 cells in the presence of different concentrations of the two compounds **3** (A) and **8** (B). Each bar represents the mean ± SEM of two experiments performed in triplicate. One-way ANOVA analysis: ****p < 0.0001.

As depicted in Figure 2, at their EC_50_ values (around 40 nM), the two compounds showed a very negligible cytotoxic effect at 48 h of incubation (around 20%), while at higher dose (10 µM) they reach 30% of cytotoxic effect, compared to untreated cells.

**Figure 3.**
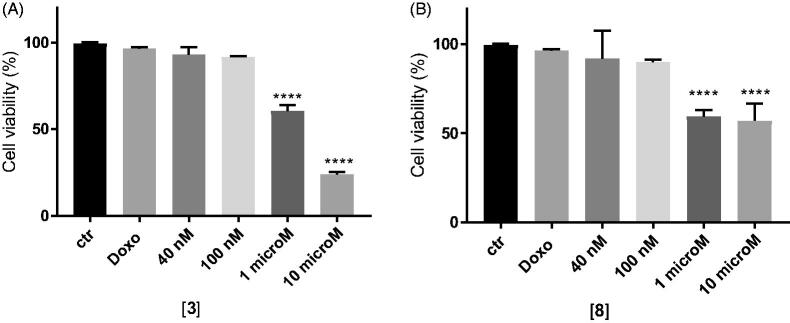
*In vitro* cell growth experiments performed on MDCK-MDR1 cells in the presence of 10 μM doxorubicin (Doxo) alone and in the presence of different concentrations of the tested compounds **3** (A) and **8** (B). Each bar represents the mean ± SEM of two experiments performed in triplicate. One-way ANOVA analysis: ****p < 0.0001.

In MDCK cells overexpressing P-gp, doxorubicin alone at 10 µM did not show cytotoxicity as expected, while the co-administration with the two compounds at 1 and 10 µM was able to rehabilitate the effect of the antineoplastic agent leading to high cytotoxicity. In particular, the co-administration of doxorubicin with compounds **3** and **8** at their higher dose (10 µM) showed cytotoxicity values of 77% for **3** and around 50% for **8** (Figure 3).

The efficacy of the compounds was next tested in cancer cell lines with various levels of P-gp, as the human adenocarcinoma colon cells (HT29) and non-small cell lung cancer cells (A549) displaying low amount of P-gp, and their resistant counterparts HT29/DX and A549/DX, with a high amount of P-gp^53^. In preliminary assays, we verified that **3** and **8** were not cytotoxic at all the tested concentration (data not shown).

**Figure 4.**
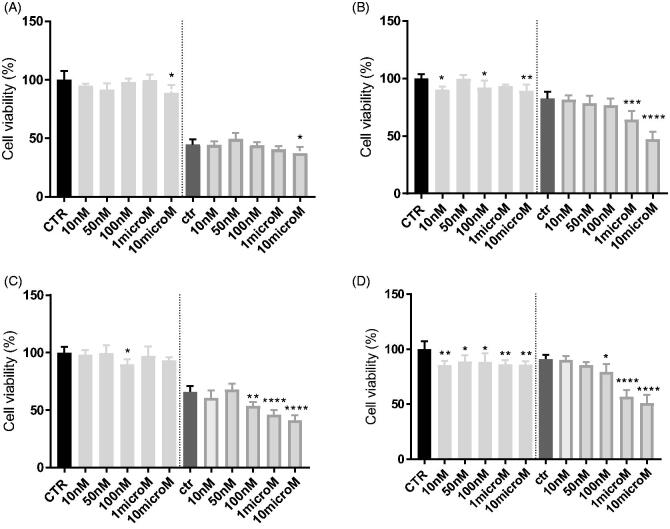
*In vitro* cell growth experiments performed on HT29 (A), HT29/DX cells (B), A549 (C) and A549/DX cells (D) in the presence of compound **3** alone at different concentrations and in co-administration of 10 μM doxorubicin (Doxo) (after the dotted line). Each bar represents the mean ± SEM of two experiments performed in triplicate. CTR represents the untreated cells, while ctr is the same cell lines treated with 10 μM Doxo alone. One-way ANOVA analysis: *p < 0.05; **p ≤ 0.005; ****p ≤ 0.0001.

**Figure 5.**
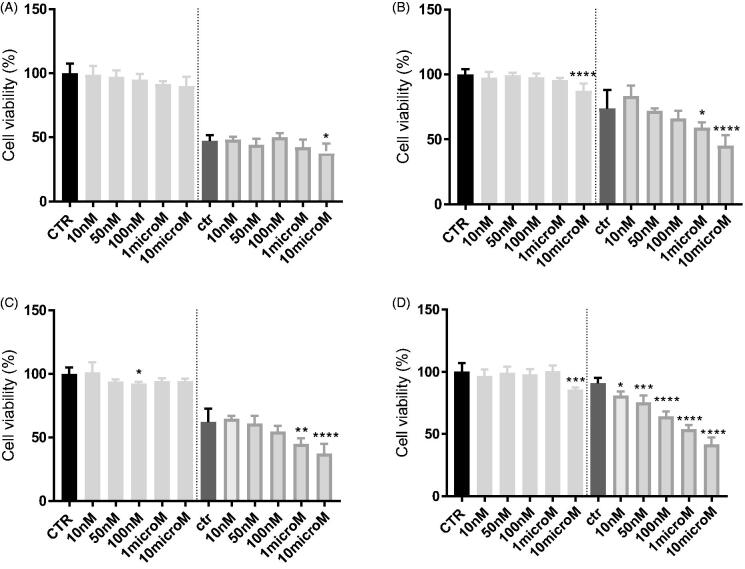
*In vitro* cell growth experiments performed on HT29 (A), HT29/DX cells (B), A549 (C) and A549/DX cells (D) in the presence of compound **8** alone at different concentrations and in co-administration of 10 μM doxorubicin (Doxo) (after the dotted line). Each bar represents the mean ± SEM of two experiments performed in triplicate. CTR represents the untreated cells, while ctr is the same cell lines treated with 10 μM Doxo alone. One-way ANOVA analysis: *p < 0.05; **p ≤ 0.005; ***p ≤ 0.001; ****p < 0.0001.

In HT29 cells, which express low levels of P-gp^53^, neither **3** (Figure 4) nor **8** (Figure 5) increases the cytotoxicity, measured as reduced viability, of doxorubicin. By contrast, in the resistant counterpart HT29/DX, which have higher levels of the transporter, **3** significantly increased the reduction of cell viability induced by doxorubicin when used at 1 or 10 µM (Figure 4). The same results were obtained in A549 and in A549/DX cells, characterised by high levels of P-gp^53^. A similar trend was observed also for **8** (Figure 5). More in details, at 10 µM, **3** reduced the cell viability of 5% and of 35% in doxorubicin-treated HT29 cells (Figure 4(A)) and HT29/DX cells (Figure 4(B)), respectively. In A549 and A549/DX cells it decreased the viability of 25% (Figure 4(C)) and 40% (Figure 4(D)), respectively. The co-administration with doxorubicin of compound **8**, at 10 µM, reduced cell viability of 10% in HT29 cells (Figure 5(A)), 29% in HT29/DX cells (Figure 5(B)), 25% in A549 cells (Figure 5(C)) and 49% in A549/DX cells (Figure 5(D)).

Notably, HT29 cells had very low/undetectable levels of P-gp^54^, while A549 cells had slightly higher levels of P-gp; by contrast, the corresponding resistant cells display high (HT29/DX) and huge (A549/DX) amount of P-gp^54^. The higher toxicity of the combination of compounds **3** or **8** plus doxorubicin in resistant cells, in particular in A549/DX subline, confirms that they potentiate cytotoxicity of doxorubicin by inhibiting P-gp activity and arousing a higher intracellular accumulation of the antineoplastic agent in P-gp expressing cells.

### Chemical stability test

Due to the presence of ester or amide groups in the structure of compounds **1**–**26**, a study aimed to evaluate their susceptibility to spontaneous or enzymatic hydrolysis, was planned. For this purpose, a series of experiments were carried out, adding a known amount of analyte in both phosphate buffer solution (PBS) and in human plasma. The samples were analysed by LC-MS/MS method operating in Multiple Reaction Monitoring (MRM) mode^55^.

The solution stability of each studied compound was verified by monitoring the variation of the analyte concentration at different incubation times in PBS and human plasma samples. The processed raw data and the used LC-MS/MS methods are reported in Supplemental material.

The analyte concentration (1 µM) used during the stability tests is generally smaller than the corresponding Michaelis–Menten constant (*K*_M_); therefore, the enzymatic degradation rate is described by a first-order kinetic. Then, by plotting the natural logarithm of the quantitative data versus the incubation time, a linear function can be used and its slope represents the degradation rate (k). Accordingly with the linear function, the half-life (*t*_1/2_) of each tested compound can be calculated as follows:

t1/2= ln (0.50µM)k

The plots of the natural logarithm of the quantitative data versus the incubation time of all the studied compounds were analysed. The obtained results demonstrated that all the compounds were stable in PBS and most of them also in human plasma. In particular, all the amide derivatives were not susceptible to the enzymatic hydrolysis, while the degradation plots of the ester compounds **15**, **16**, **18**, **24**, **25**, and **26** in human plasma showed a significant decay rate (*t*_1/2_ values between 6 and 124 min). The calculated half-life values of all tested compounds were reported in Table 1. The stable compounds showed the *k* values close to 0; consequently, for these derivatives, extremely high half-life can be calculated. Since under the proposed experimental conditions, a half-life over 240 min is not correctly evaluated, it is reasonable to consider that their half-life values could be equal or greater than 240 min. Furthermore, the half-life value of ketoprofene ethylester (KEE), used as reference compound, demonstrated that the employed human batch was enzymatically active (*t*_1/2_ < 2 h)^55^.

**Figure 6.**
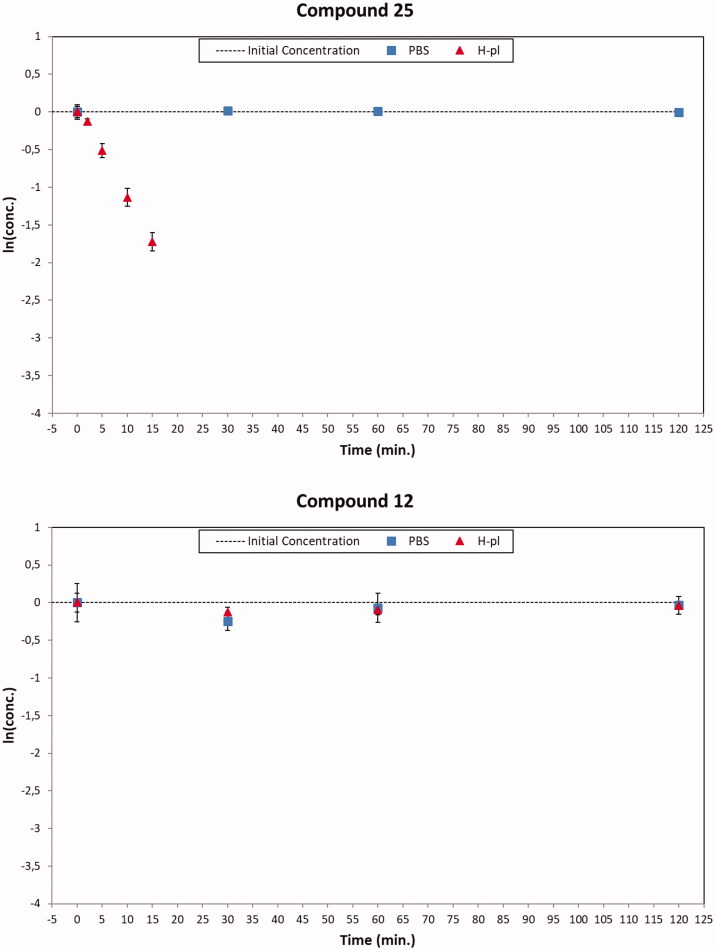
Degradation plots of the ester compound **25** (top) and the corresponding amide derivative **12** (bottom) in PBS (square blue) and human plasma (red triangle) samples. The ester compound **25** suffered a quickly enzymatic hydrolysis (*t*_1/2_ about 6 min), while the amide derivative **12** was stable over 120 min in human plasma samples. Both the concentration profiles resulted unmodified in PBS solutions.

The human plasma degradation profiles of the ester compound **25** and its amide homologous **12** are reported as an example in Figure 6. All degradation plots of the studied compounds are reported in Supplementary material.

The obtained results indicate that the ester group is more susceptible to the enzymatic hydrolysis than the corresponding amide one, since some of the ester derivatives were not stable in human plasma samples.

## Conclusions

In this work, a new series of amide and ester derivatives carrying a 6,7-dimethoxy-2-phenethyl-1,2,3,4-tetrahydroisoquinoline scaffold linked to different methoxy-substituted aryl moieties was synthesised. The obtained compounds were evaluated for their P-gp interaction profile and selectivity towards the two other ABC transporters, multidrug-resistance-associated protein-1 (MRP-1) and breast cancer resistance protein (BCRP). Most of the compounds displayed high activity versus P-gp: the presence of an amide or an ester function did not influence P-gp selectivity and interacting mechanism. In fact, among both amide and ester derivatives some very potent compounds could be found, which resulted in general to be more selective than the reference compounds tariquidar and elacridar. Moreover, in the current case the derivatives of both series appeared to be substrates belonging to the category IIB3, showing to interact in the same way with P-gp, differently from previous findings^34^.

Noteworthy, the design of the reported series allowed the identification of two potent P-gp substrates, amides **3** and **8**, that are characterised by different selectivity profiles, since compound **3** was active also on BCRP, while compound **8** was not. These two aromatic derivatives carry an ortho methoxy substituent, combined with a second methoxy residue in different positions.

The presence of amide or ester groups in the compounds described in previous work^34^ did not compromise their chemical stability to spontaneous or enzymatic hydrolysis, since they resulted stable in PBS and also in human plasma; interestingly, in the compounds described in the present work only the amides resulted stable both in PBS and in human plasma, while the degradation plots of some ester compounds in human plasma showed a significant decay rate.

Since amide derivatives **3** and **8** showed to be stable and endowed with the best P-gp activity profile, they were further tested alone and in co-administration with the antineoplastic drug doxorubicin in different cancer cell lines with various levels of P-gp. The compounds showed a significant sensitising activity towards doxorubicin in cell models that are similar to clinically observed doxorubicin-resistant cancers, indicating that the chemosensitizing effects were due to the inhibition of P-gp.

In summary, the design of the reported series allowed the identification of two potent P-gp substrates, stable both in PBS and in human plasma and able to rehabilitate the effect of the antineoplastic agent doxorubicin in different cancer cell lines.

## Supplementary Material

Supplemental Material
