# Supplementary material for: 6,7-Dimethoxy-2-phenethyl-1,2,3,4-tetrahydroisoquinoline amides and corresponding ester isosteres as multidrug resistance reversers
Source: J Enzyme Inhib Med Chem. 2020 Apr 7;35(1):974–92. doi: 10.1080/14756366.2020.1747449 (PMC7178819; doi:10.1080/14756366.2020.1747449)
Supplement: Supplemental Material [file IENZ_A_1747449_SM8215.pdf]

Supplemental Material

6,7-Dimethoxy-2-phenethyl-1,2,3,4-tetrahydroisoquinoline amides and corresponding ester isosteres as Multidrug Resistance (MDR) reversers

Laura Braconi [a], Gianluca Bartolucci [a], Marialessandra Contino [b], Niccolò Chiaramonte [a], Roberta Giampietro [b], Dina Manetti [a], Maria Grazia Perrone [b], Maria Novella Romanelli [a], Nicola Antonio Colabufo [b], Chiara Riganti [c], Silvia Dei [a*], Elisabetta Teodori [a]

[a] *NEUROFARBA Department, Section of Pharmaceutical and Nutraceutical Sciences, University of Florence, Via Ugo Schiff 6, 50019 Sesto Fiorentino (Florence), Italy*

[b] *Department of Pharmacy-Drug Sciences, University of Bari "A. Moro", Via Orabona 4, 70125 Bari, Italy*

[c] *Department of Oncology, University of Turin, Via Santena 5/bis, 10126 Turin, Italy.*

Table of Contents:

^{1}H-NMR (400 MHz), ^{13}C-APT- NMR (100 MHz) spectra of compounds **1-26**: S2-S27

Chemical stability data of compounds **1-26** S28-S43

P-gp, MRP1 and BCRP expression in MDCK, MDCK-MDR1, MDCK-MRP1 and MDCK-BCRP cells S44

^{1}H-NMR and ^{13}C-APT-NMR spectra of compound **1**

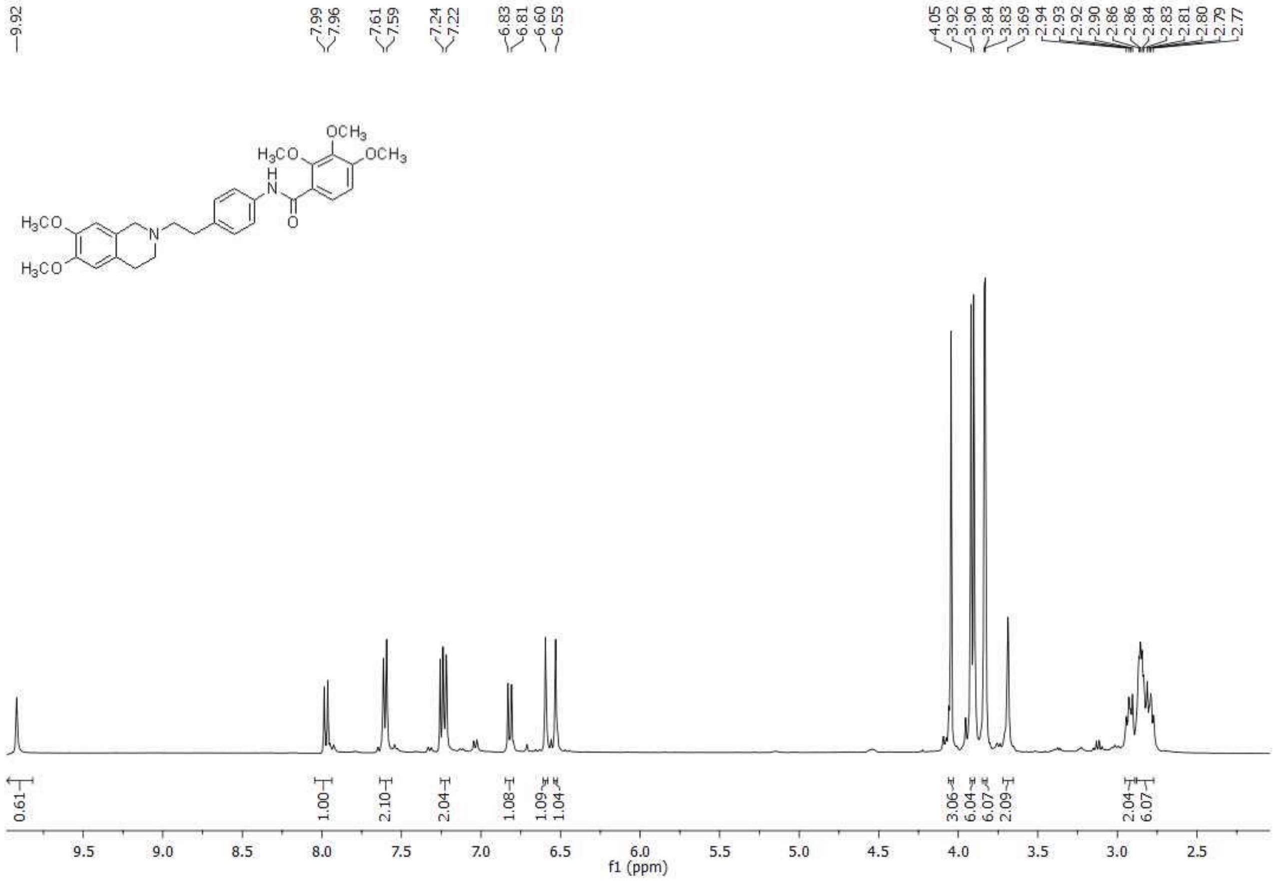

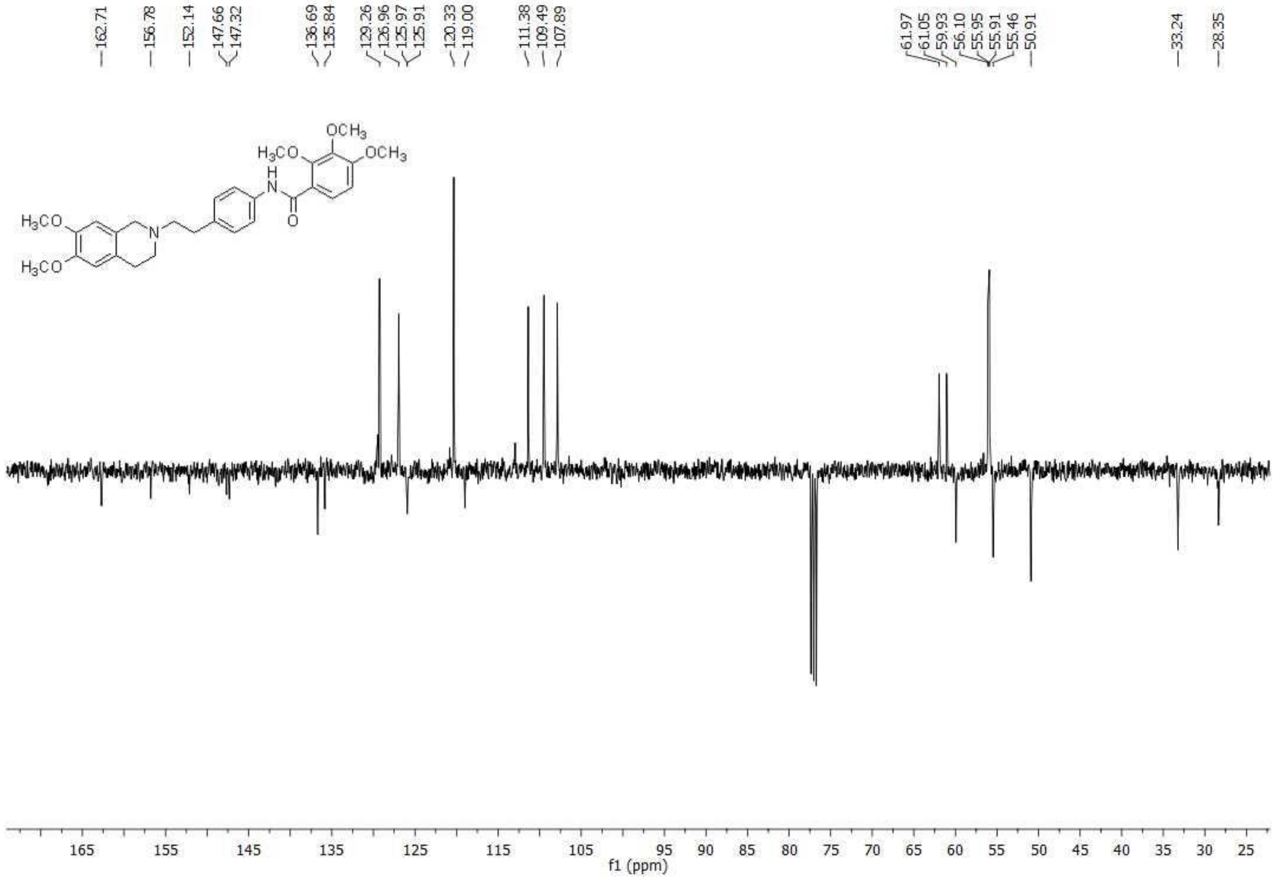

^{1}H-NMR and ^{13}C-APT-NMR spectra of compound **2**

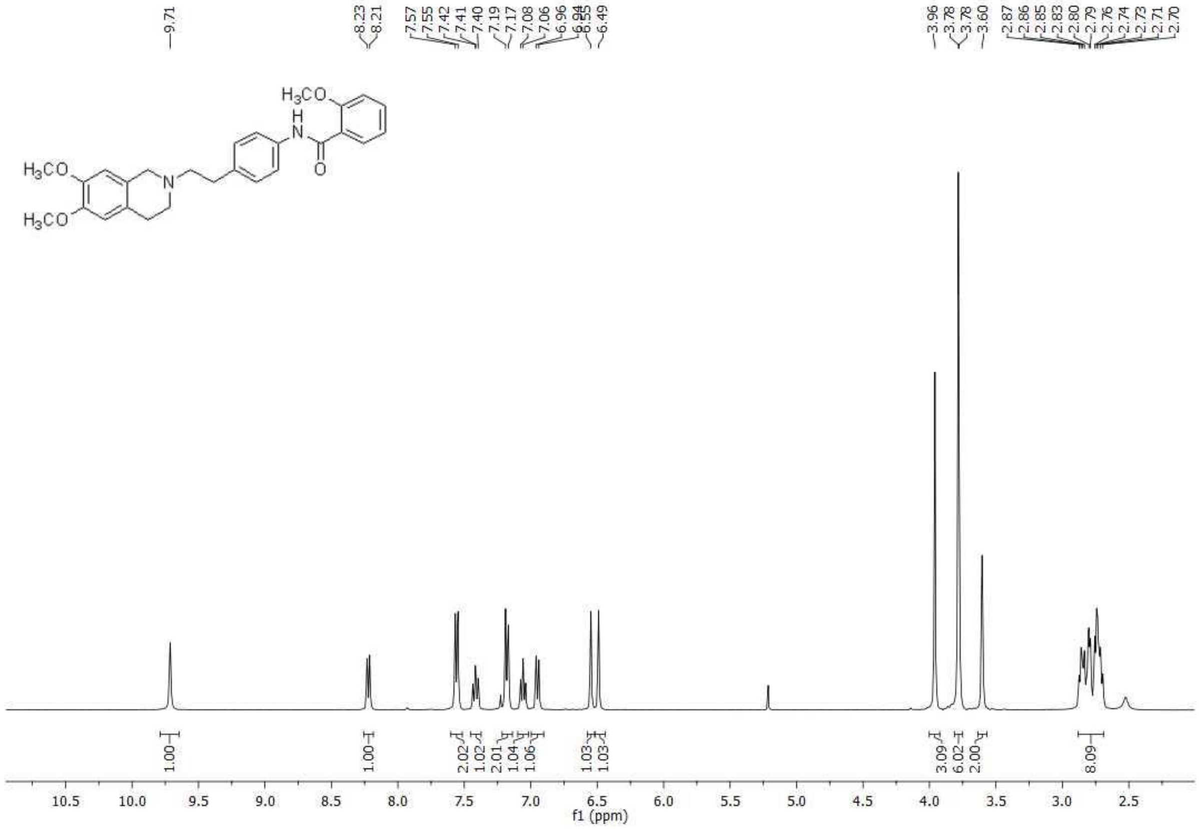

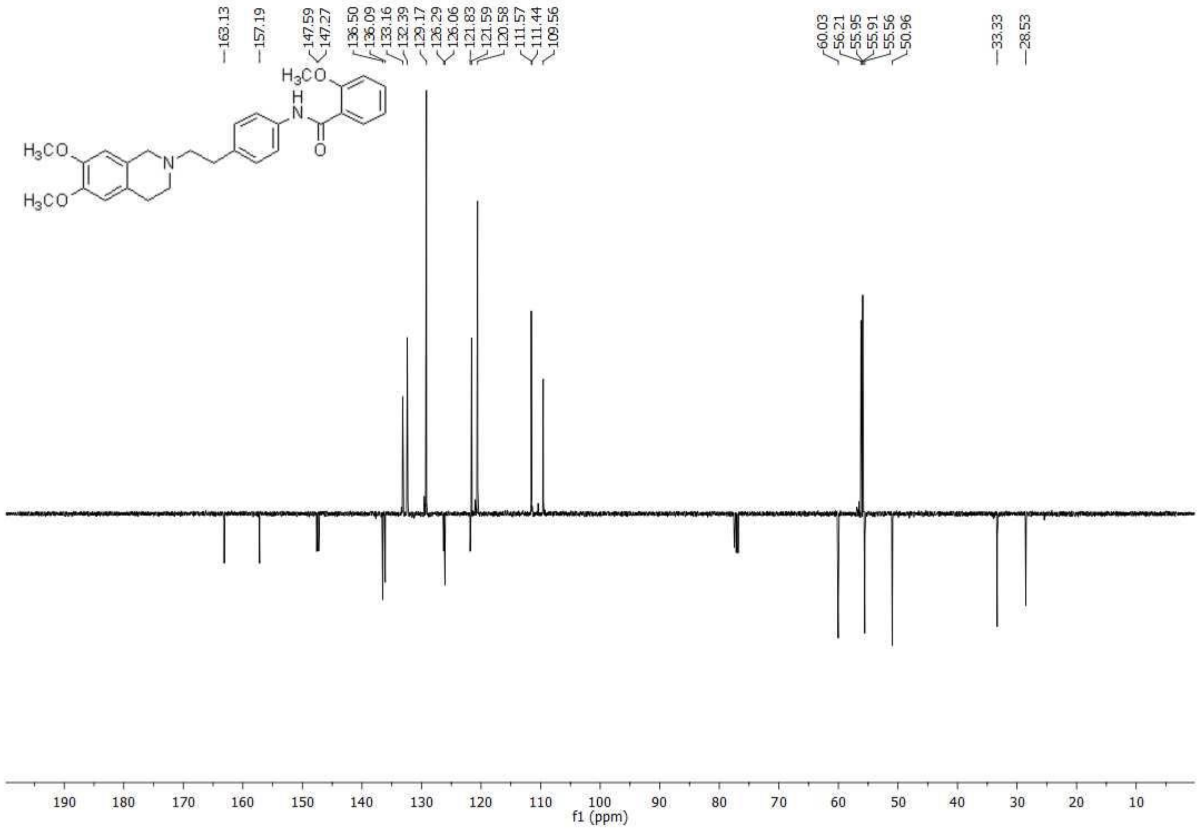

^{1}H-NMR and ^{13}C-APT-NMR spectra of compound **3**

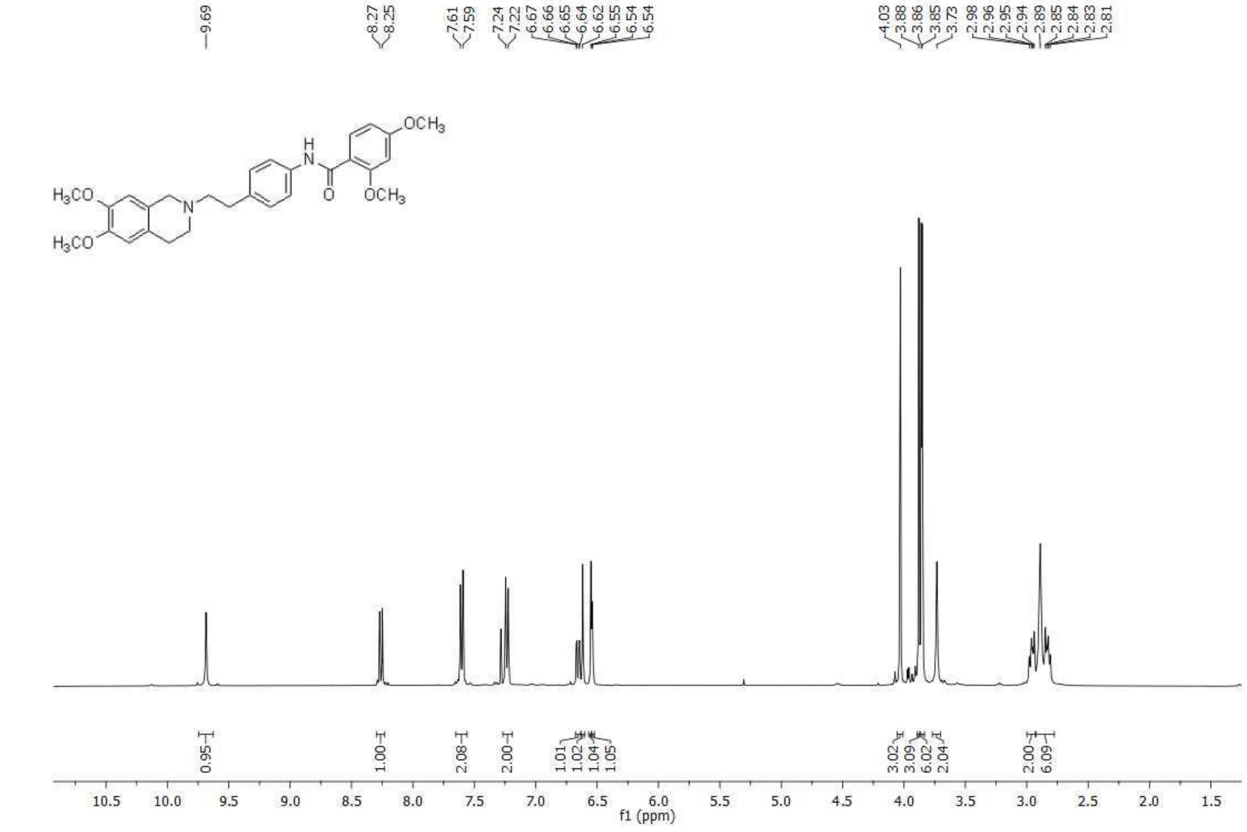

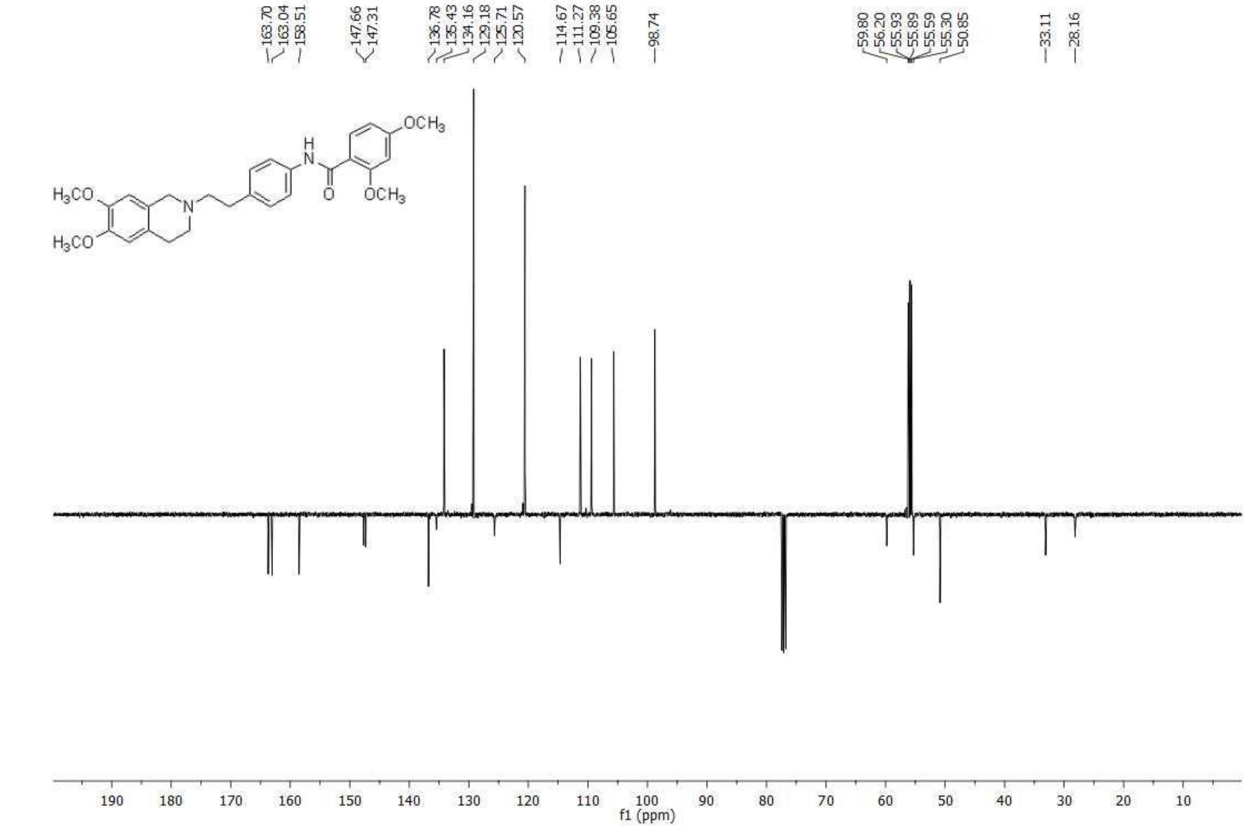

^{1}H-NMR and ^{13}C-APT-NMR spectra of compound **4**

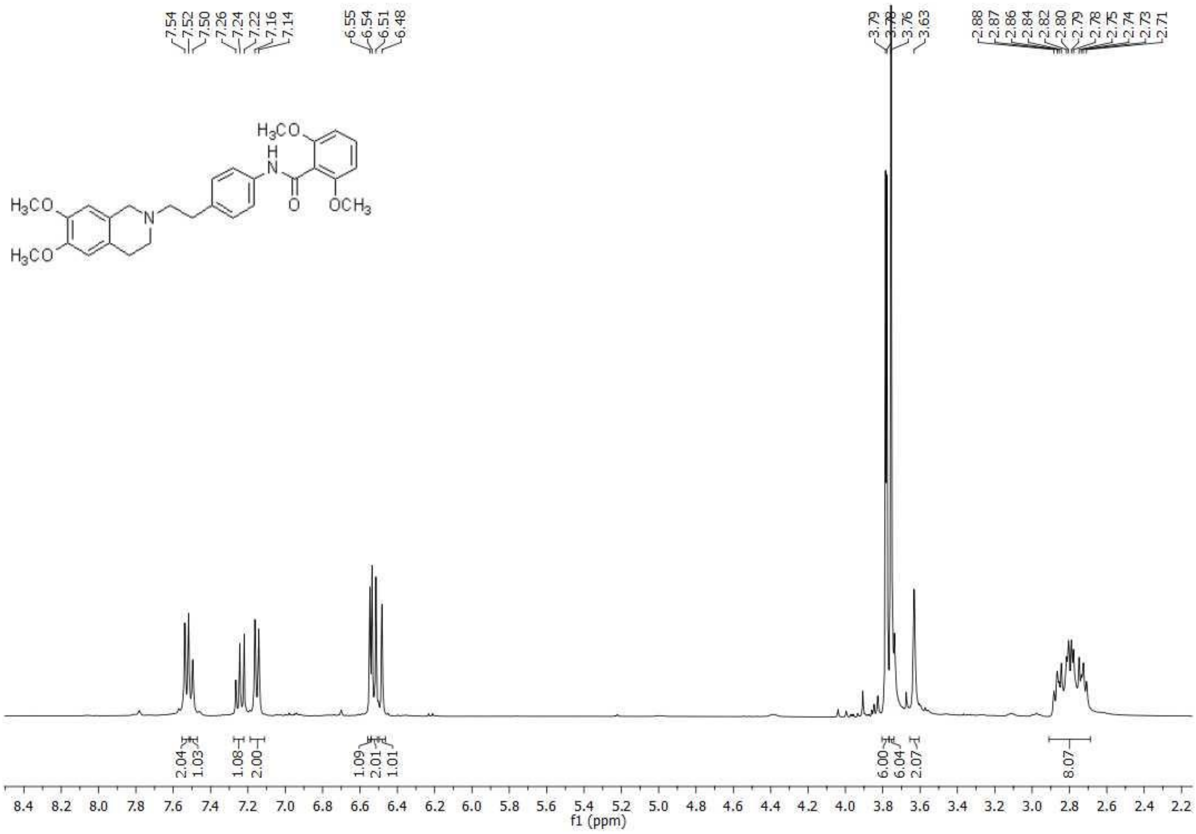

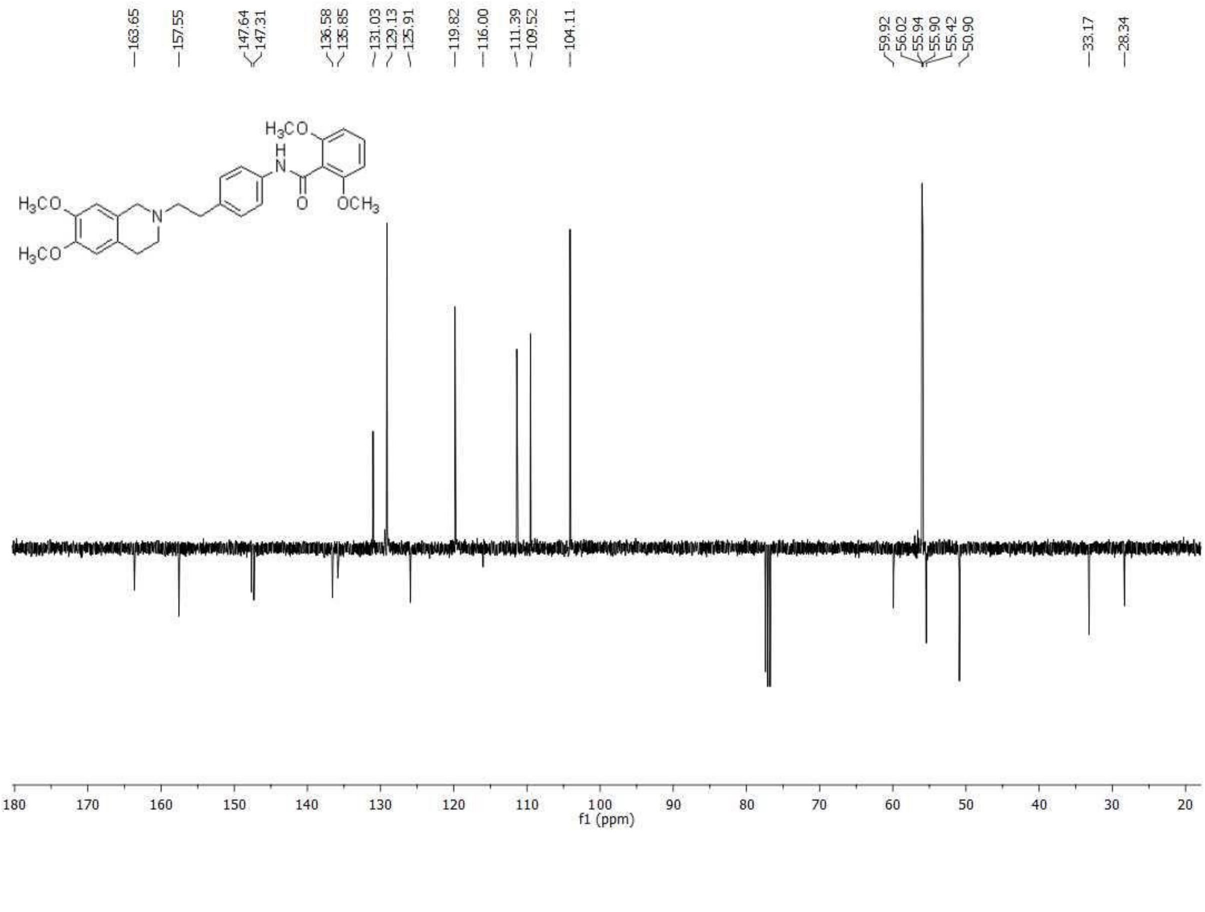

^{1}H-NMR and ^{13}C-APT-NMR spectra of compound **5**

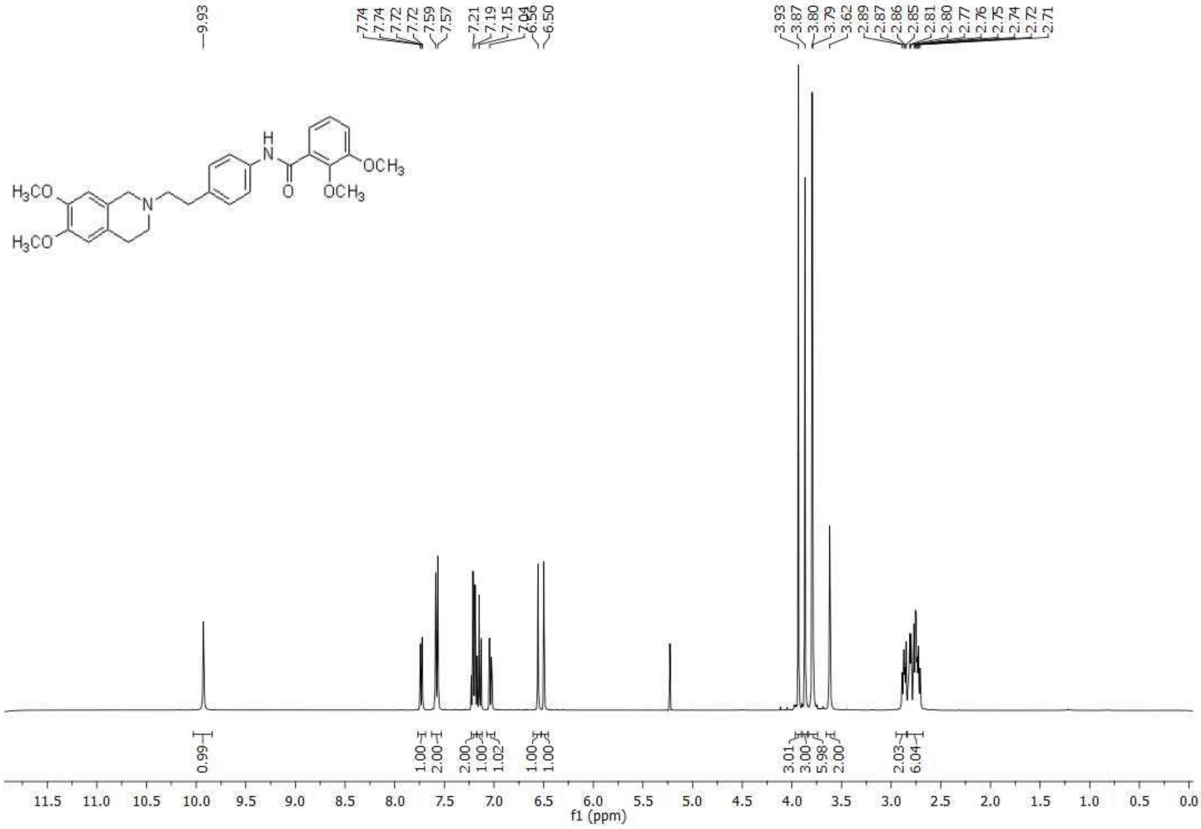

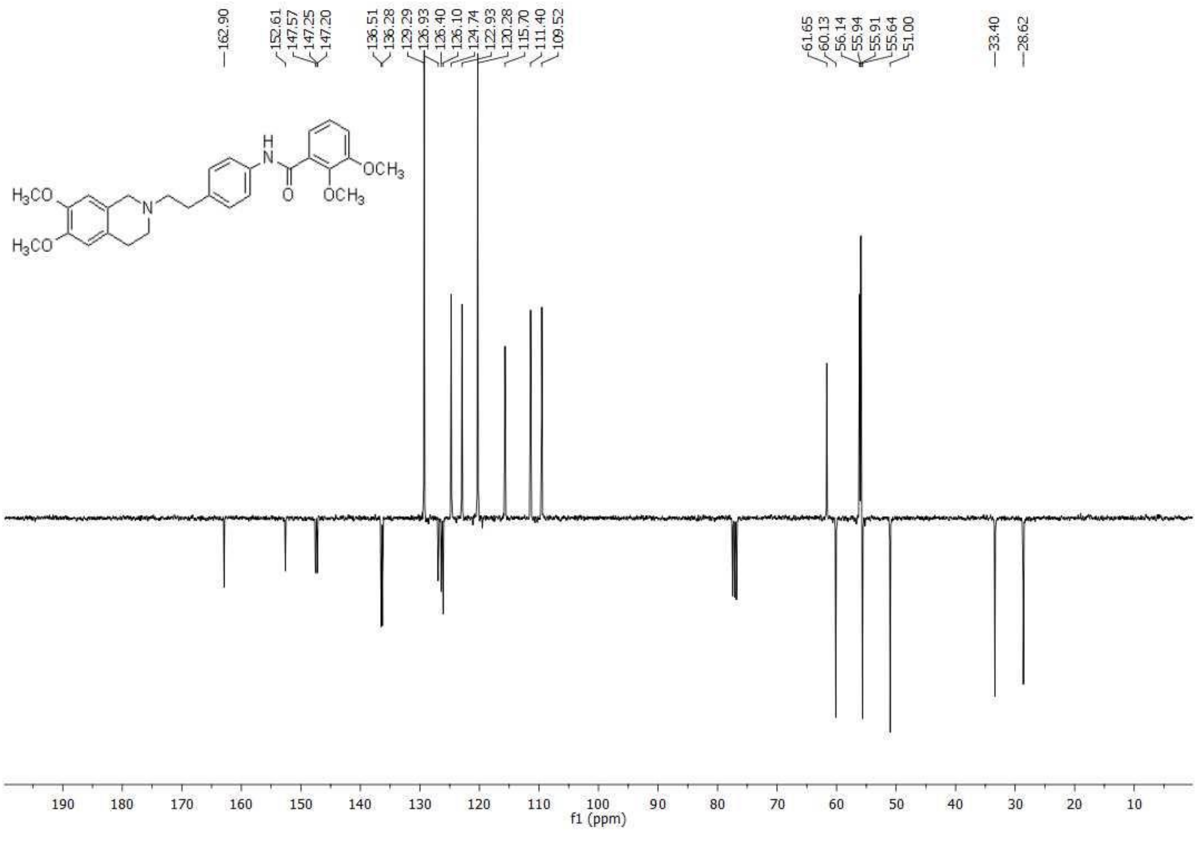

^{1}H-NMR and ^{13}C-APT-NMR spectra of compound **6**

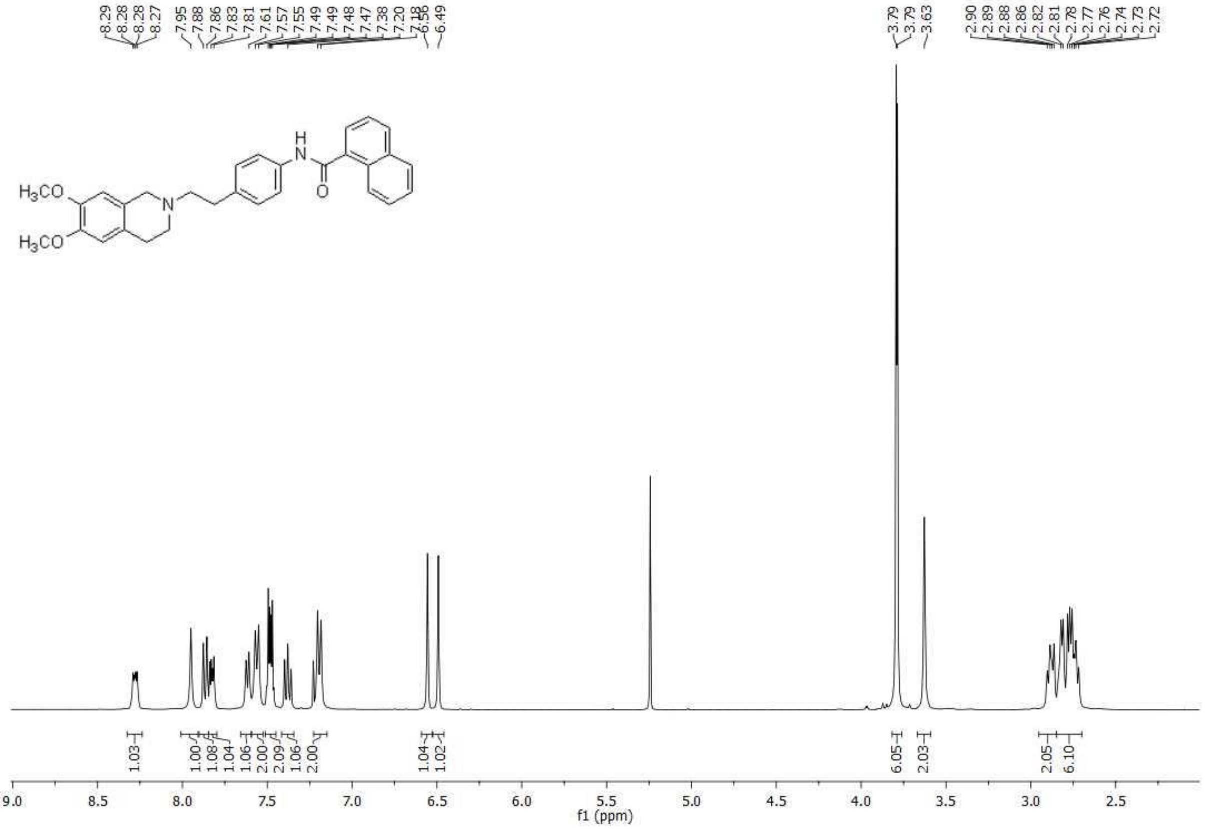

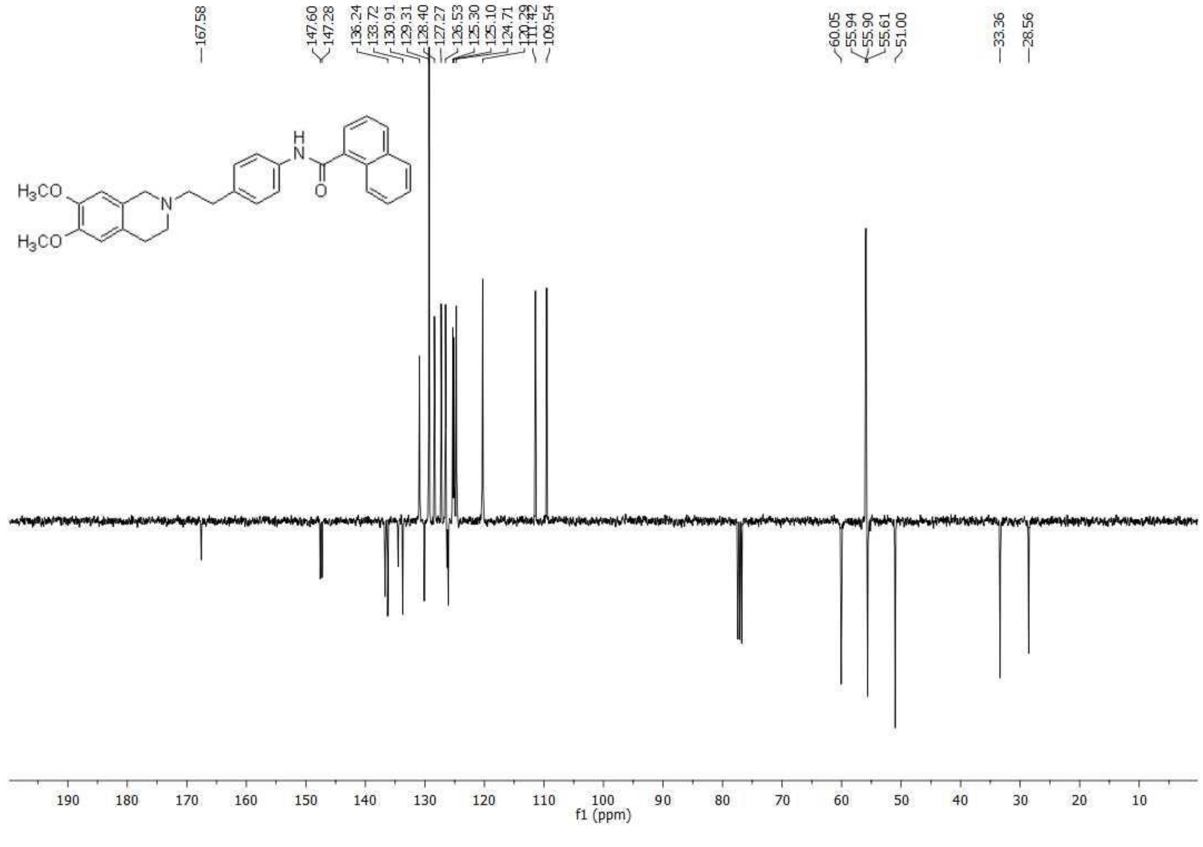

^{1}H-NMR and ^{13}C-APT-NMR spectra of compound **7**

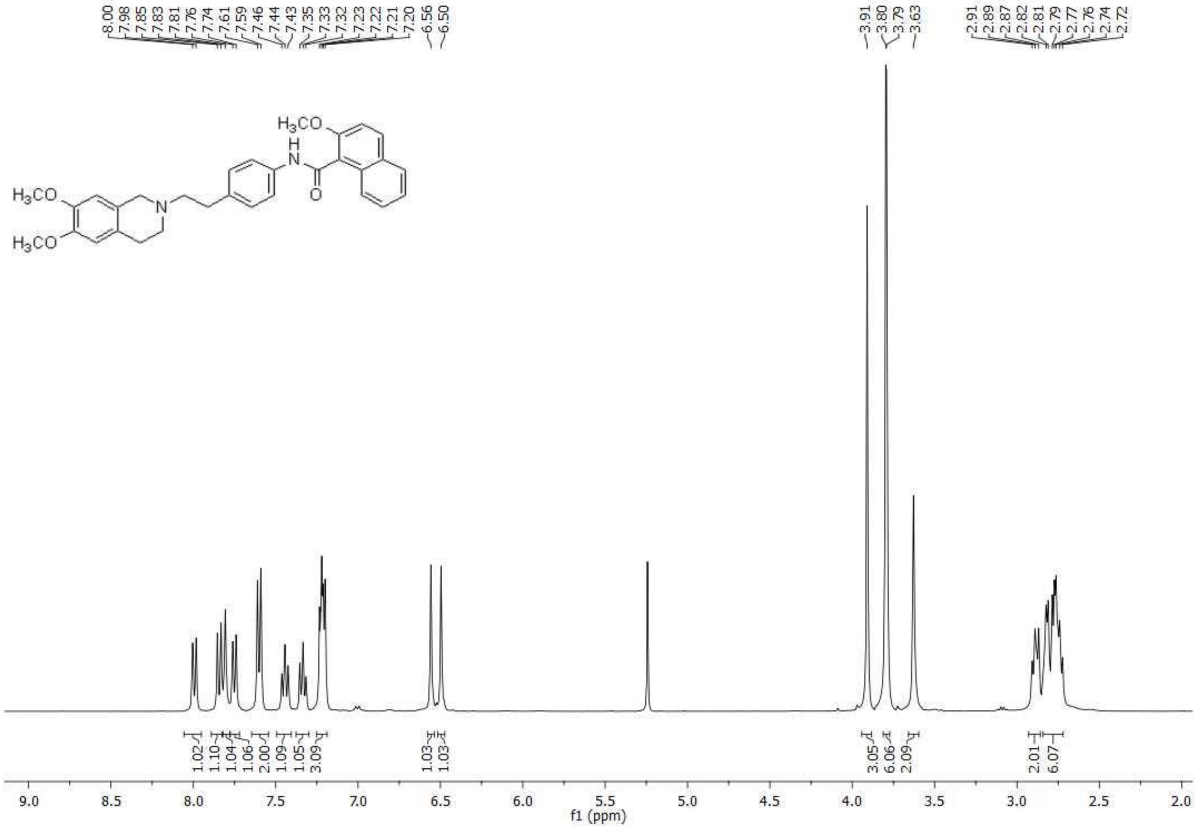

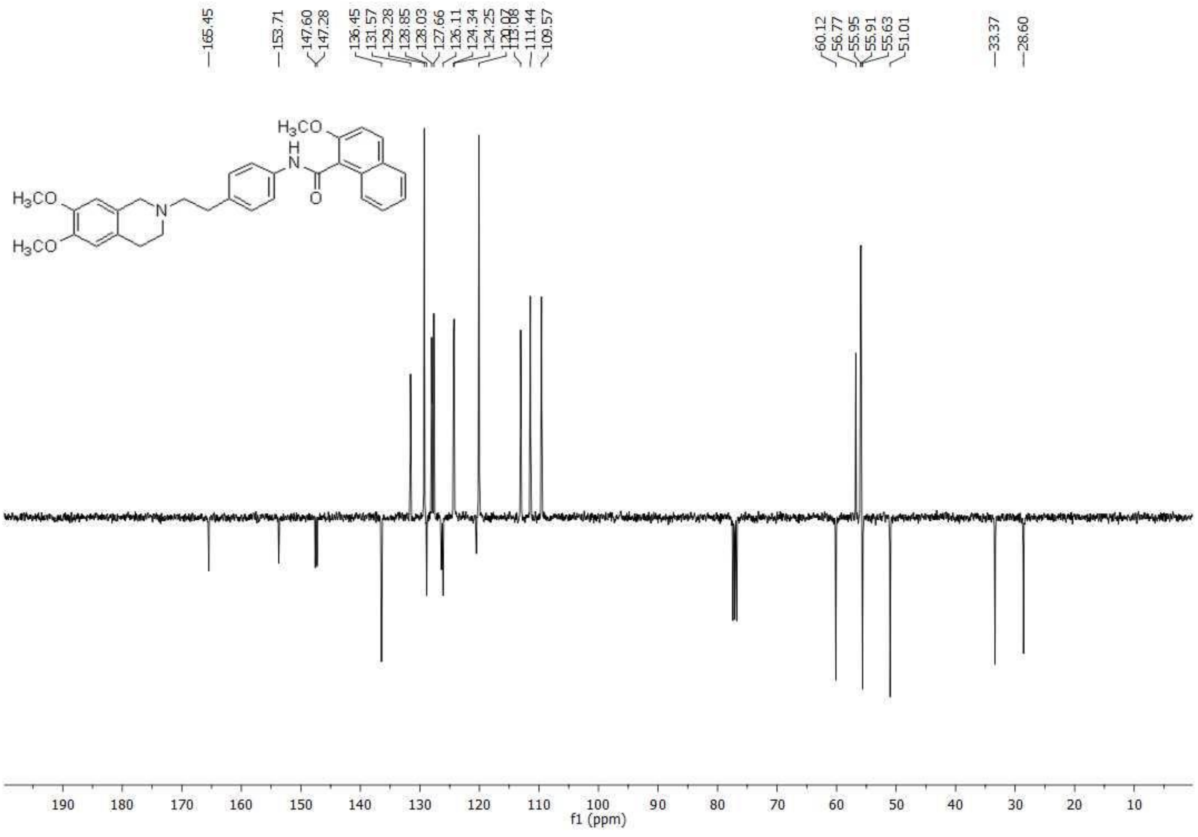

^{1}H-NMR and ^{13}C-APT-NMR spectra of compound **8**

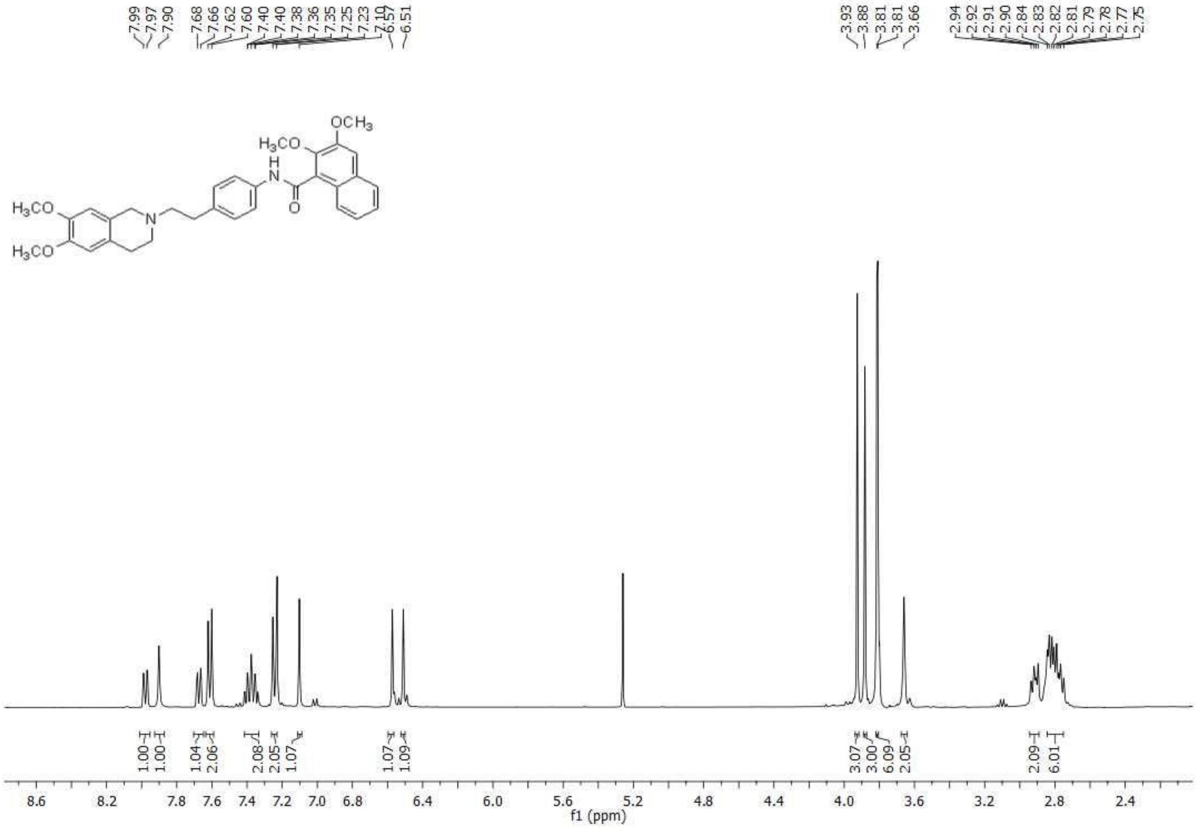

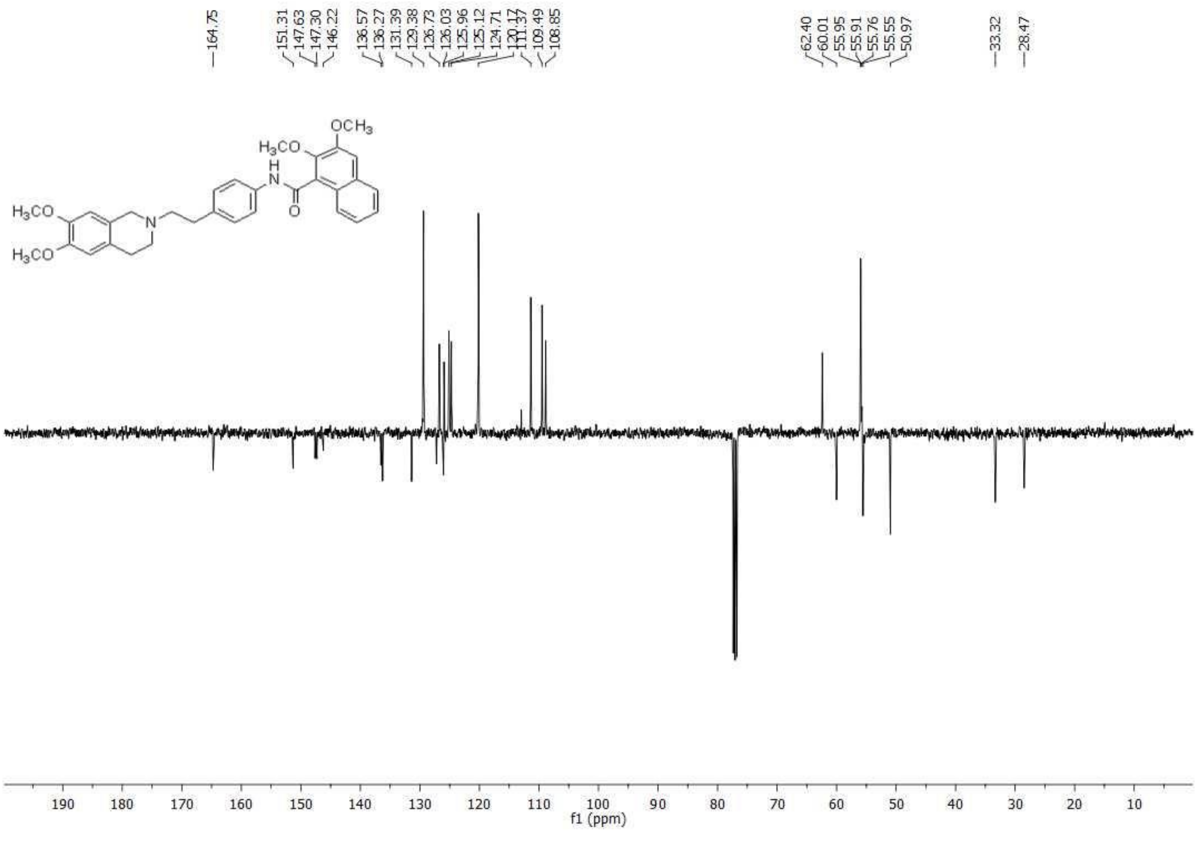

^{1}H-NMR and ^{13}C-APT-NMR spectra of compound **9**

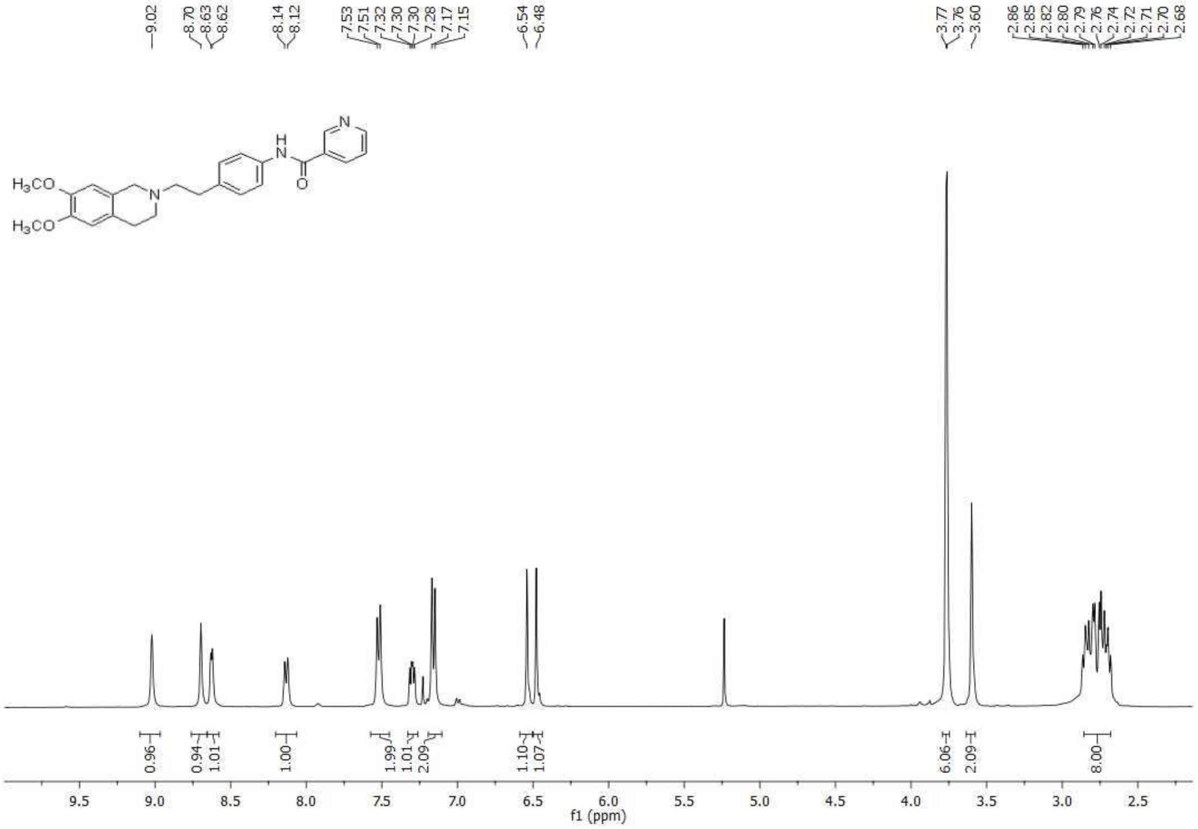

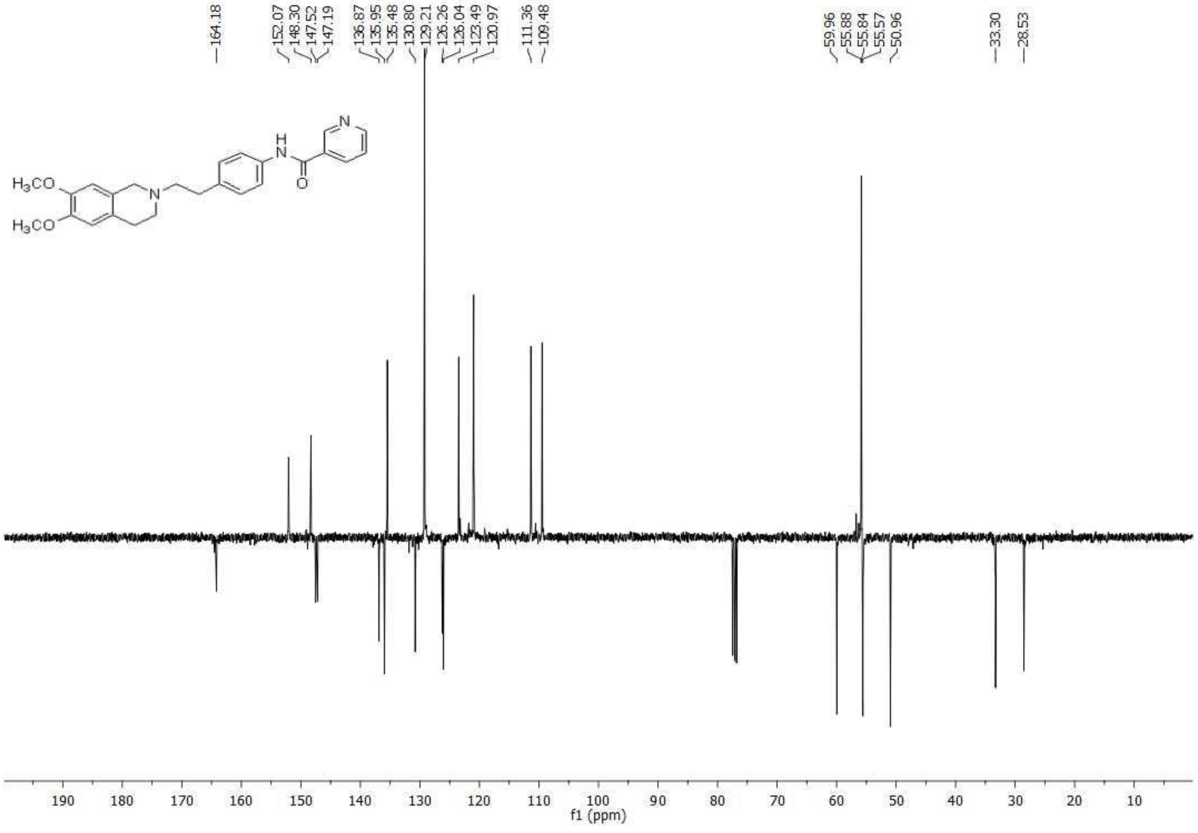

^{1}H-NMR and ^{13}C-APT-NMR spectra of compound **10**

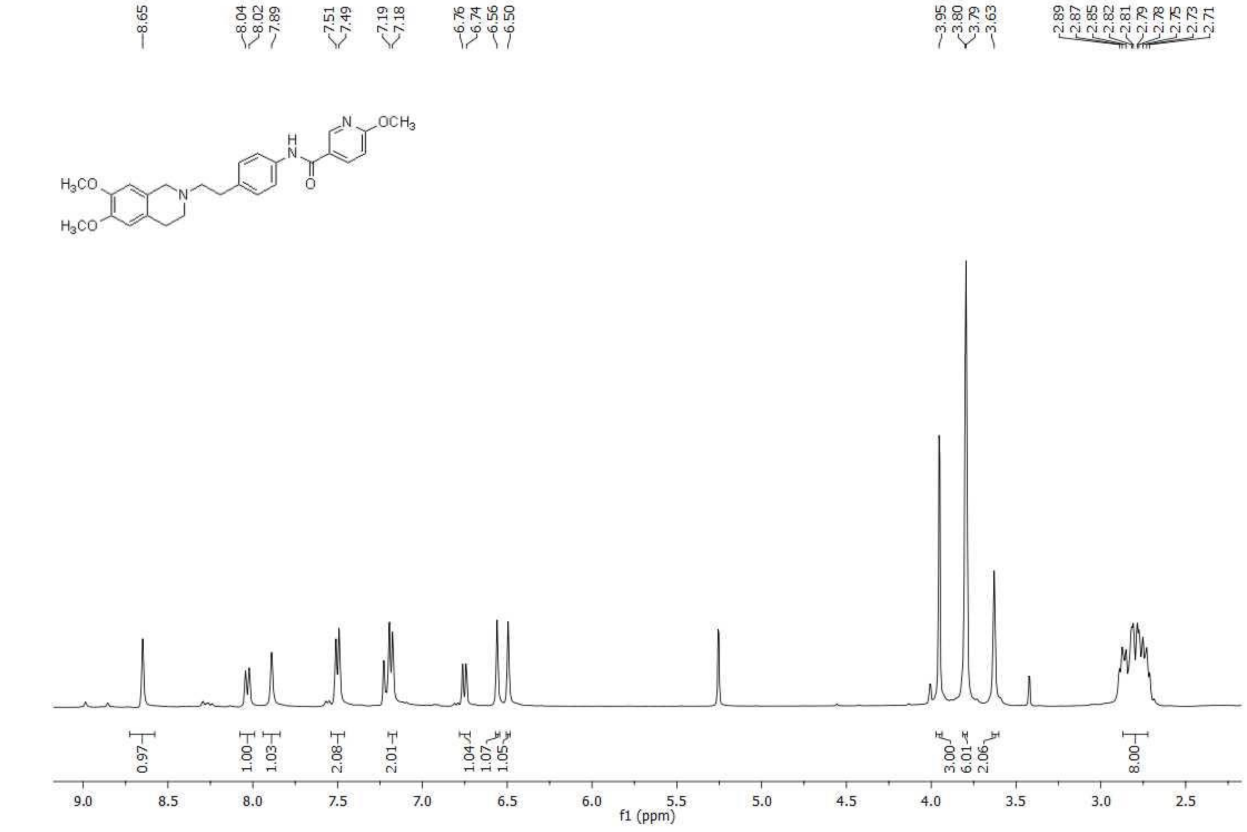

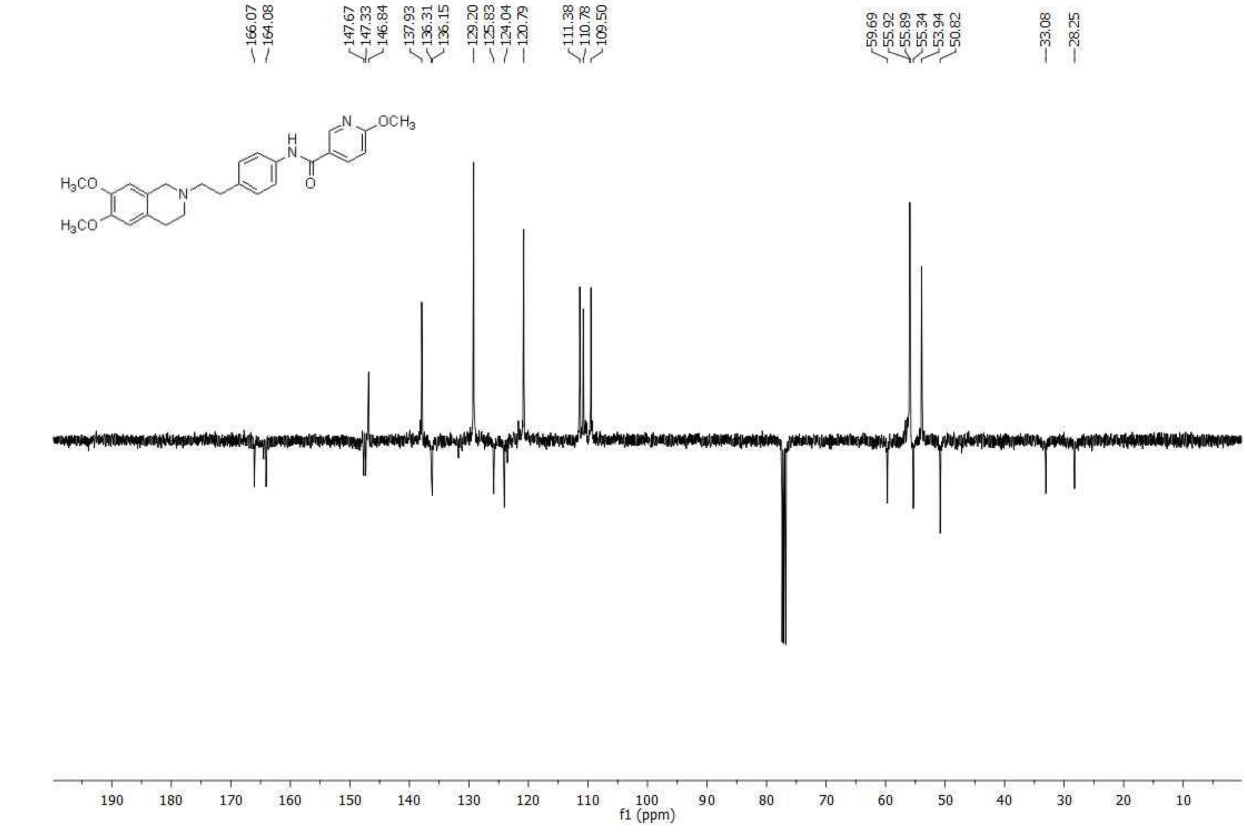

^{1}H-NMR and ^{13}C-APT-NMR spectra of compound **11**

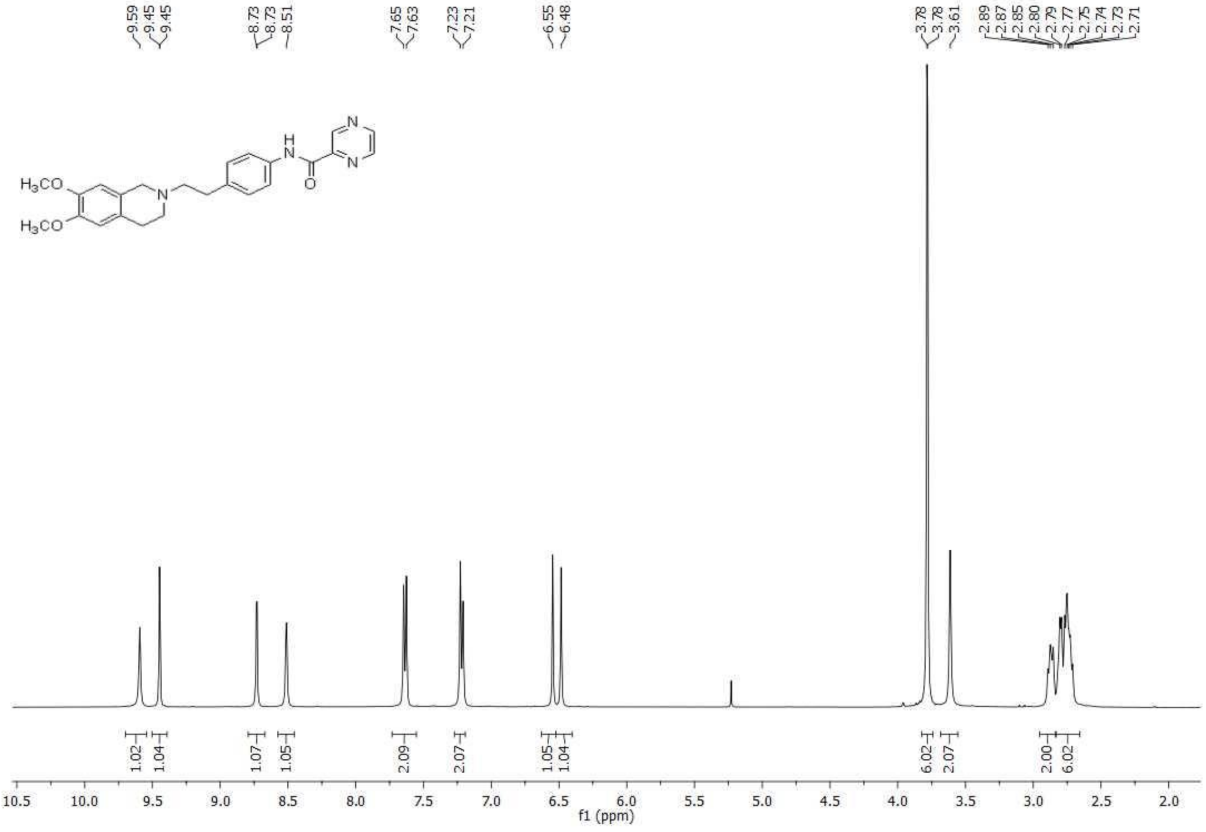

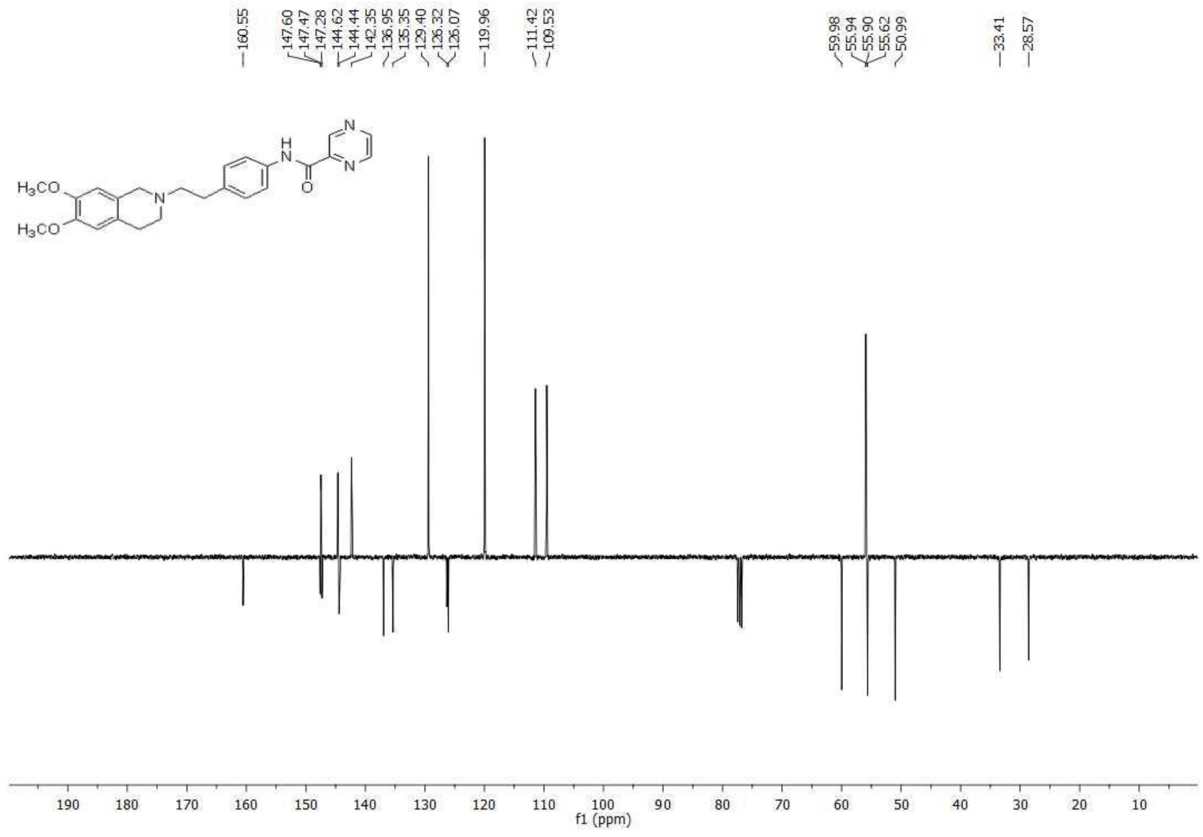

^{1}H-NMR and ^{13}C-APT-NMR spectra of compound **12**

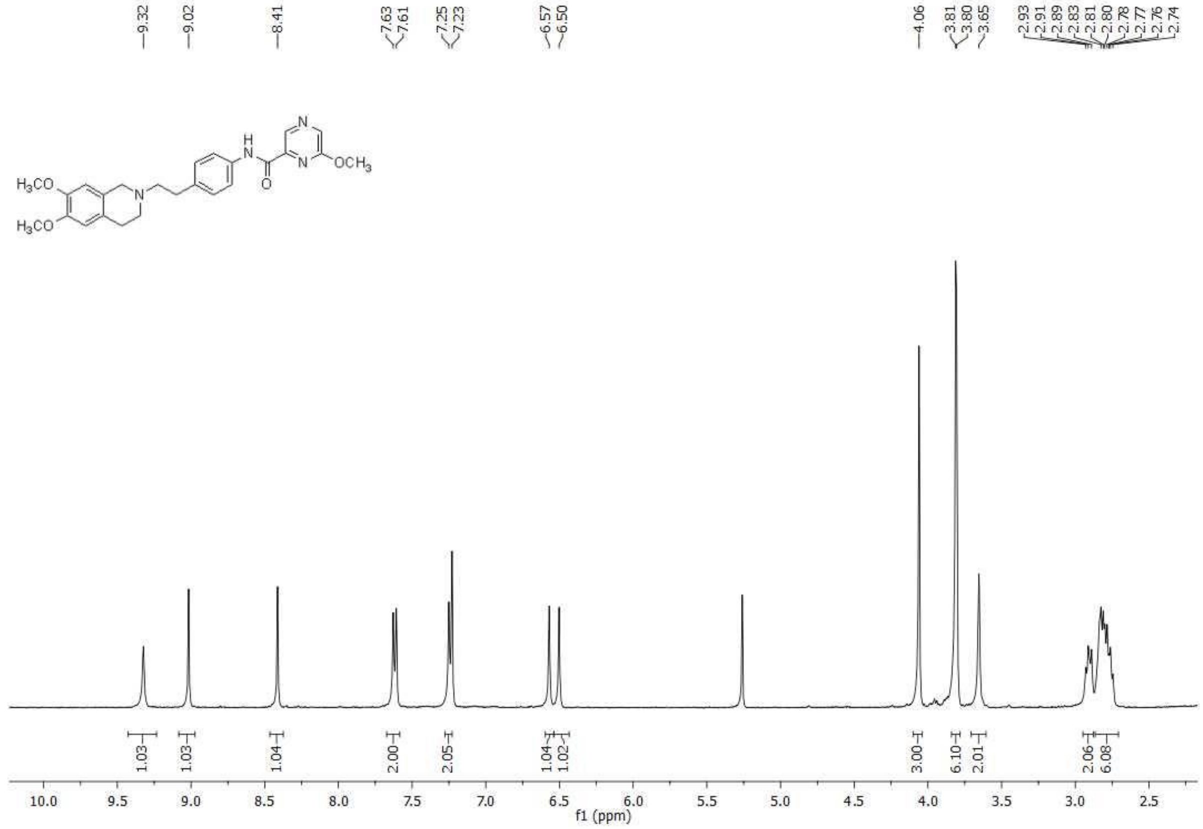

^{1}H-NMR and ^{13}C-APT-NMR spectra of compound **13**

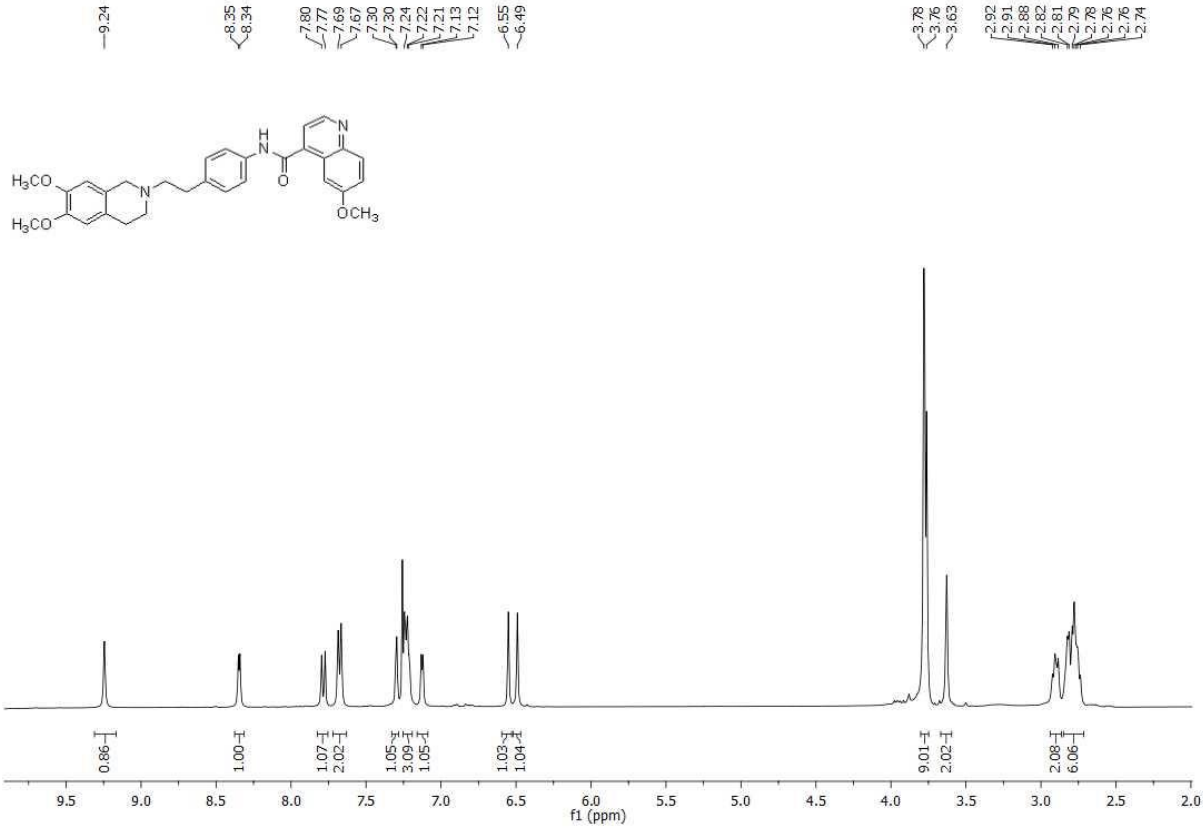

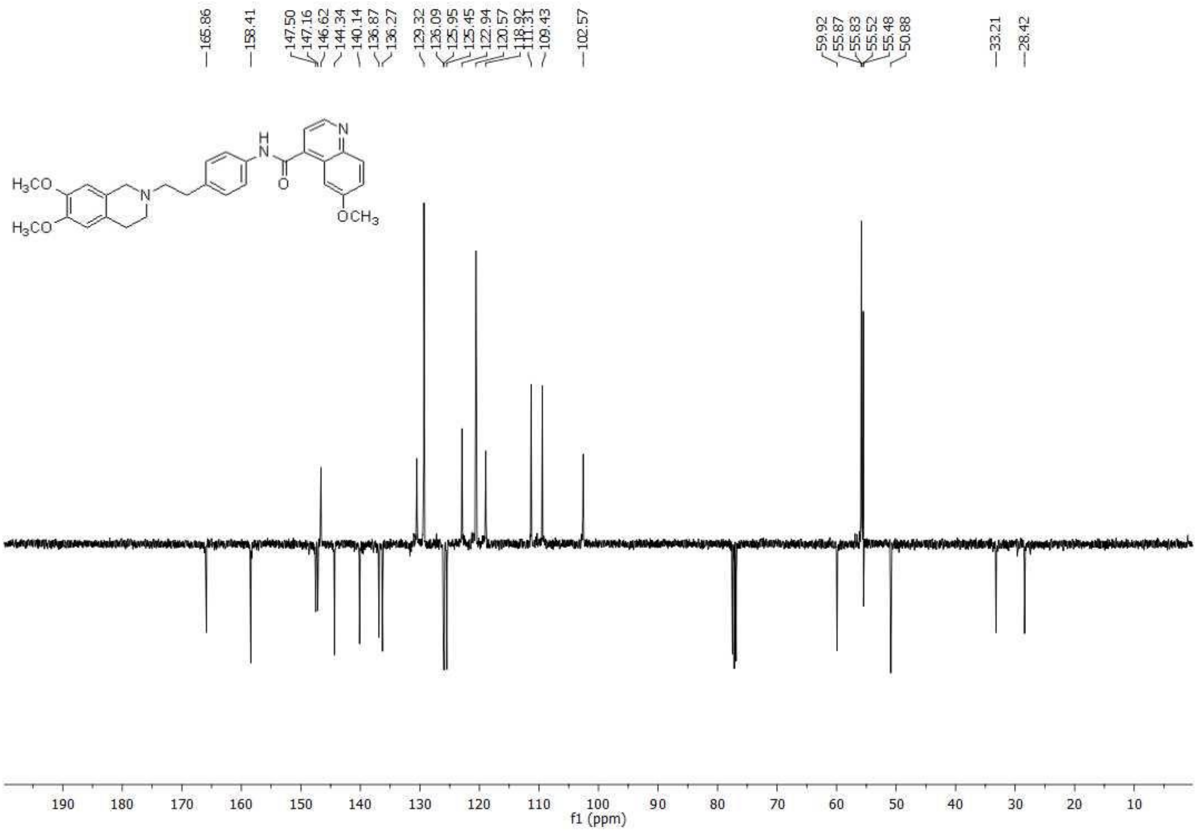

^{1}H-NMR and ^{13}C-APT-NMR spectra of compound **14**

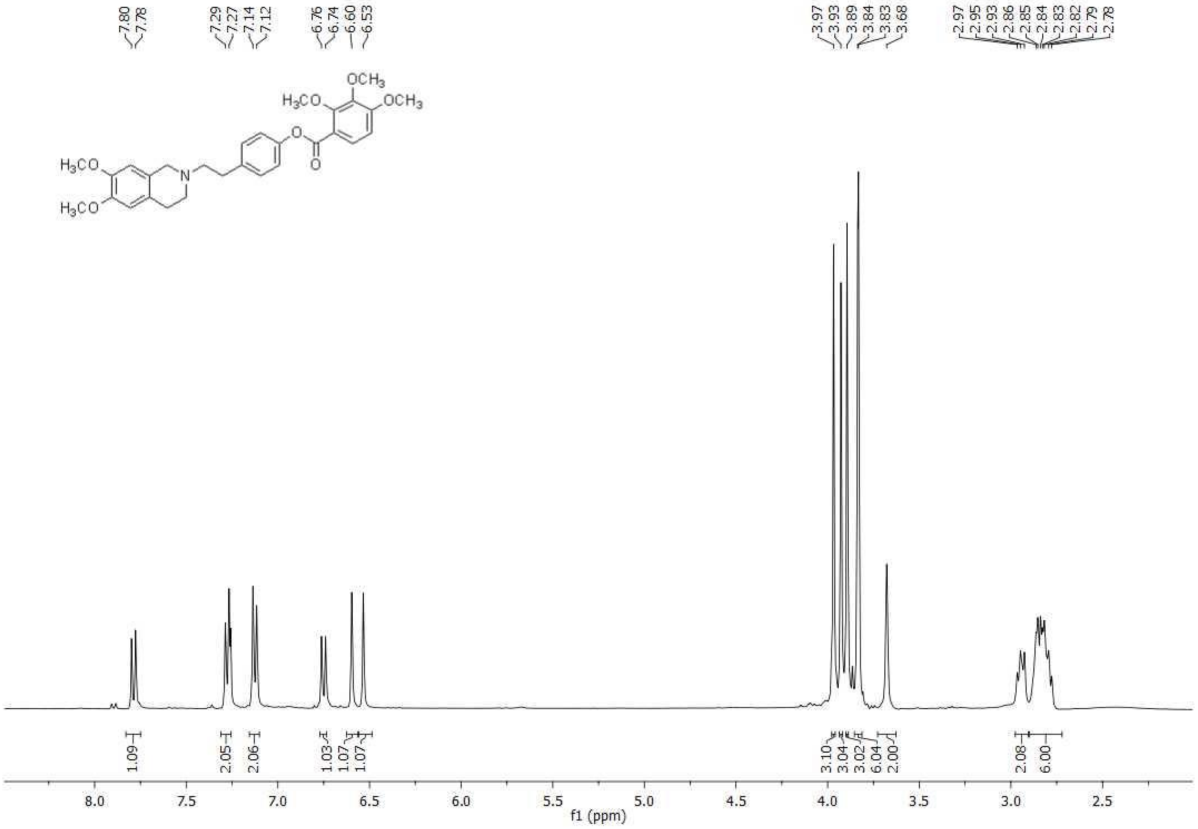

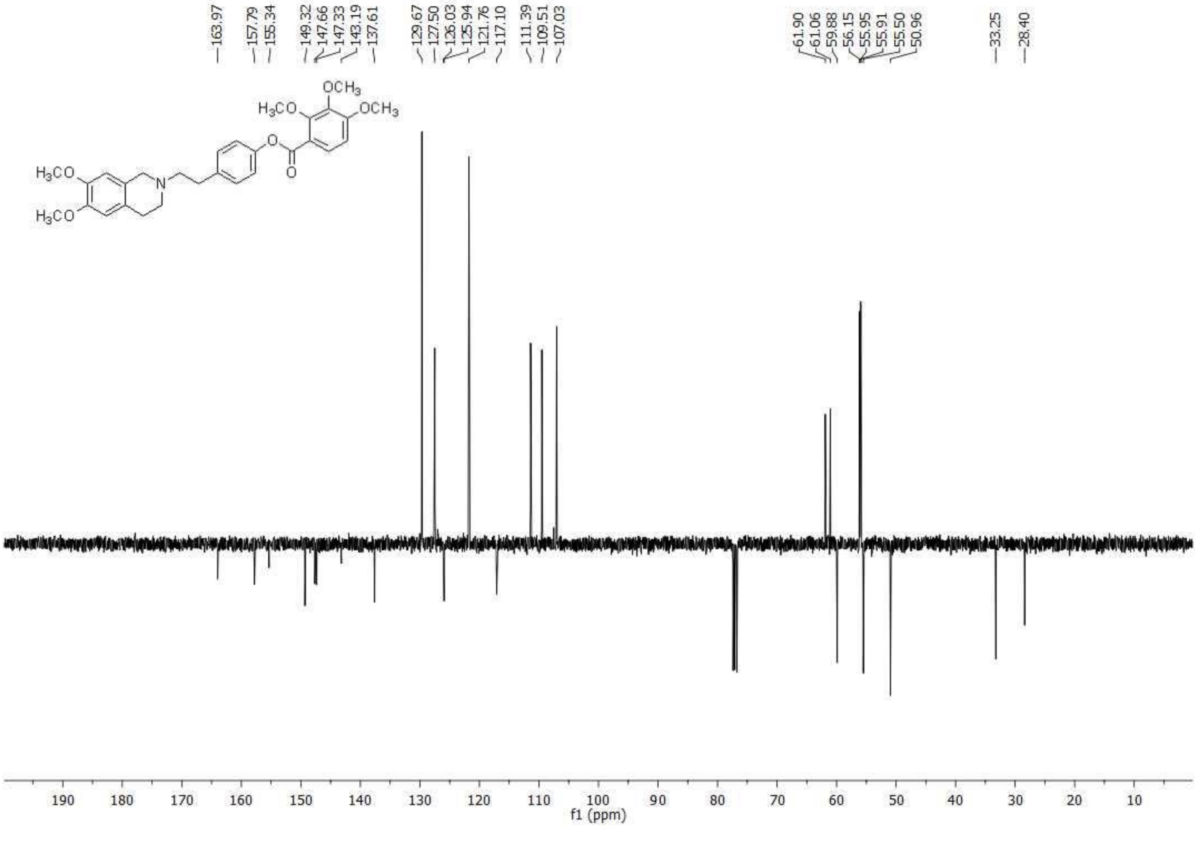

^{1}H-NMR and ^{13}C-APT-NMR spectra of compound **15**

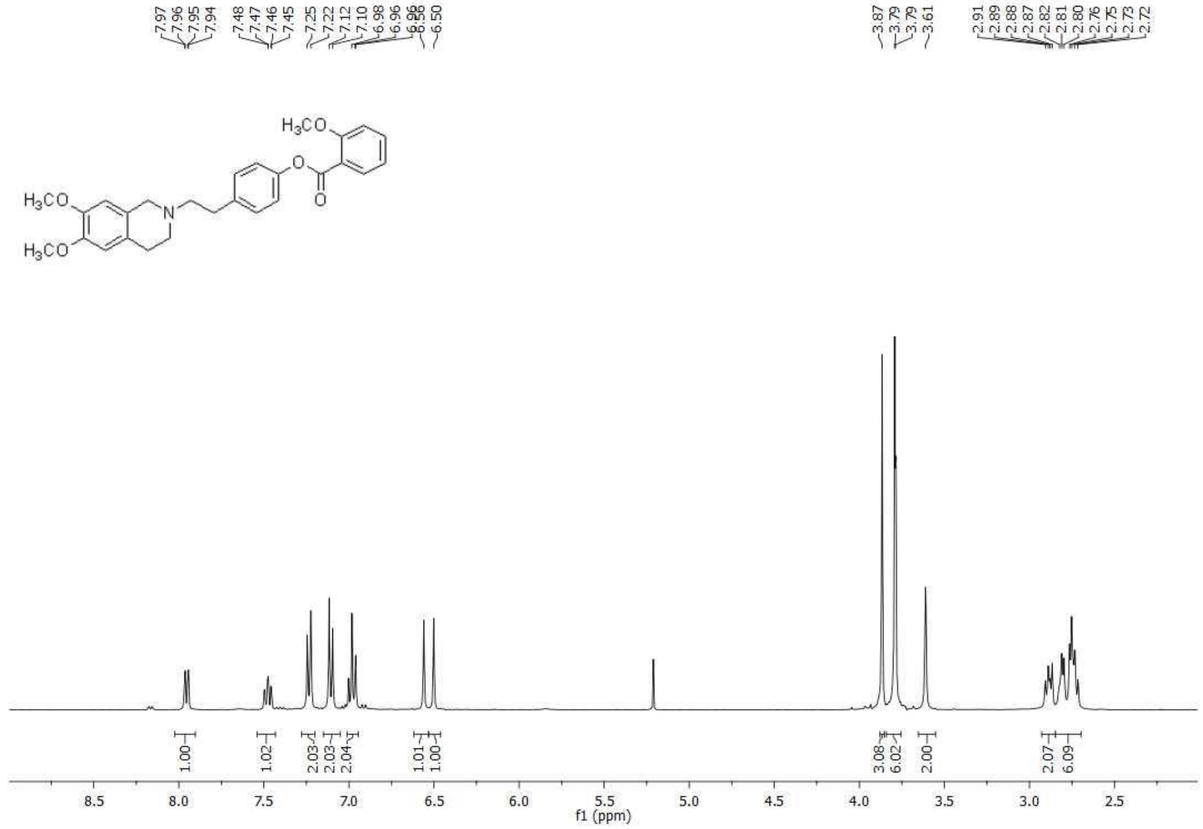

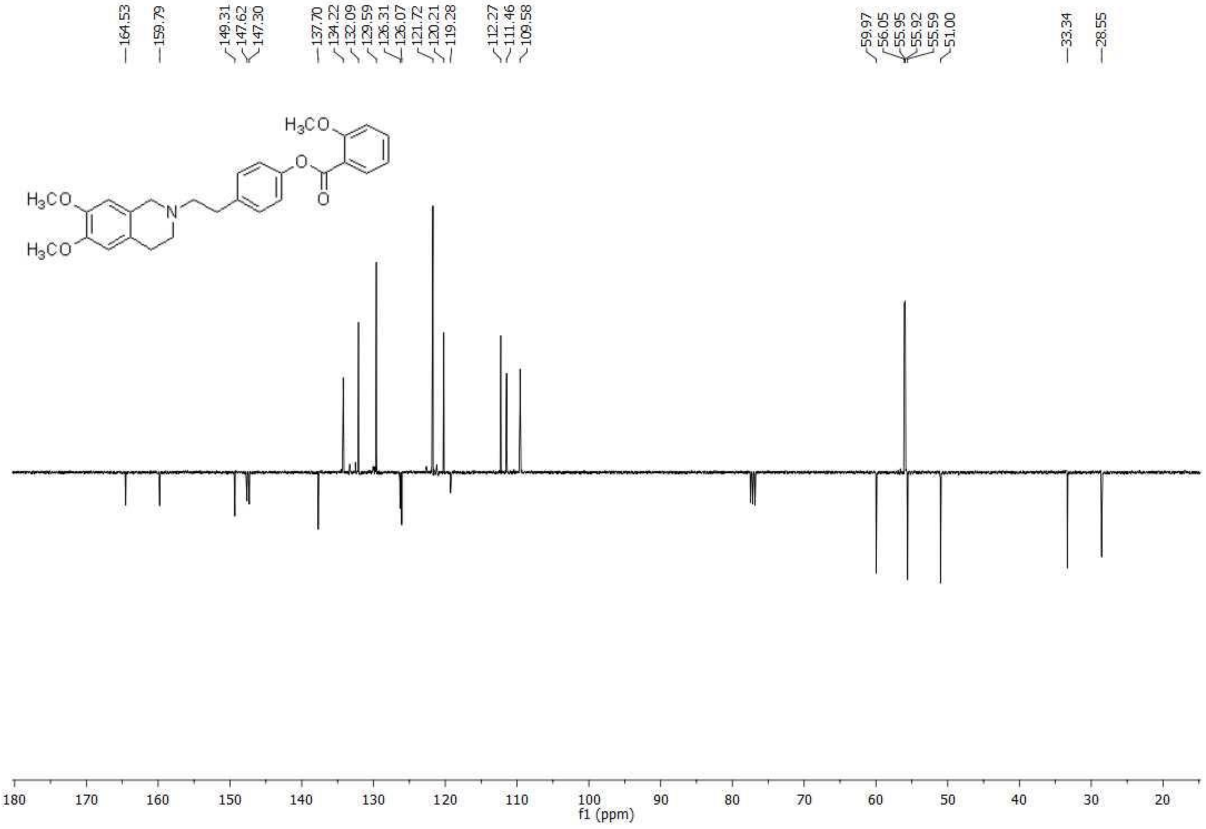

^{1}H-NMR and ^{13}C-APT-NMR spectra of compound **16**

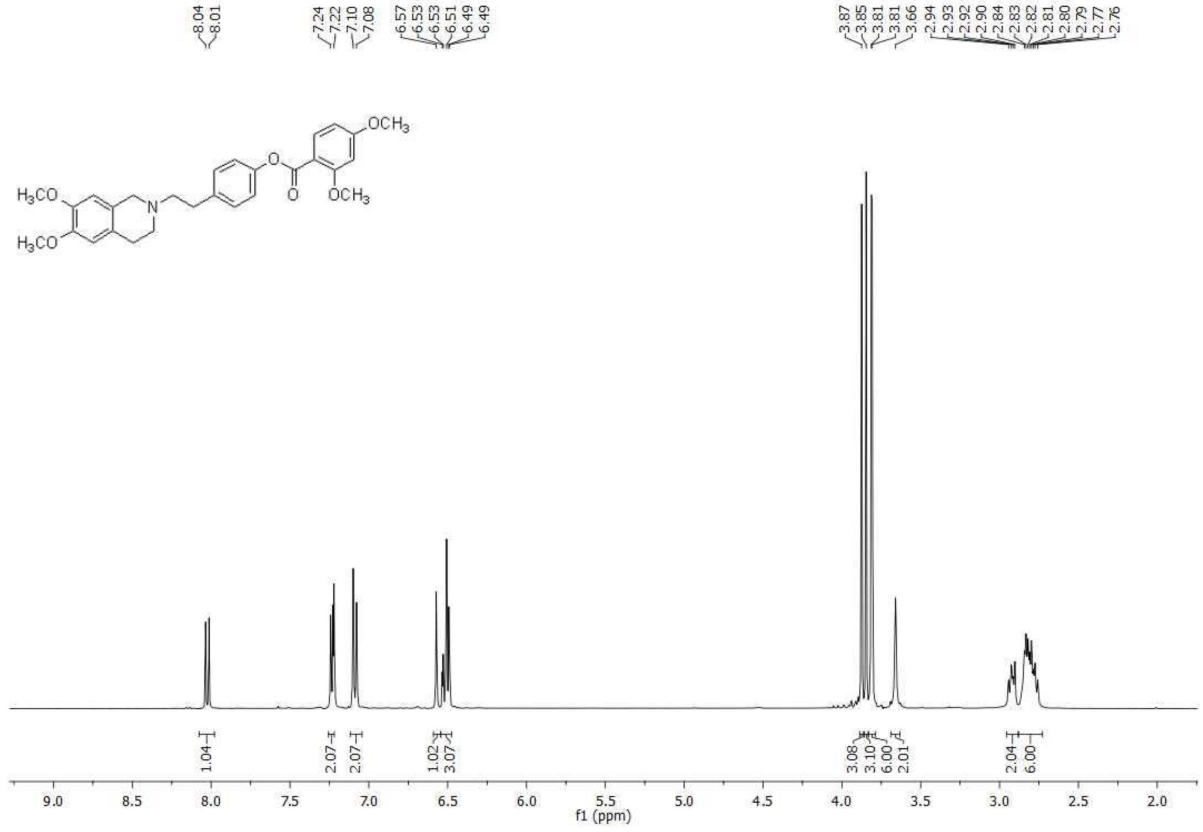

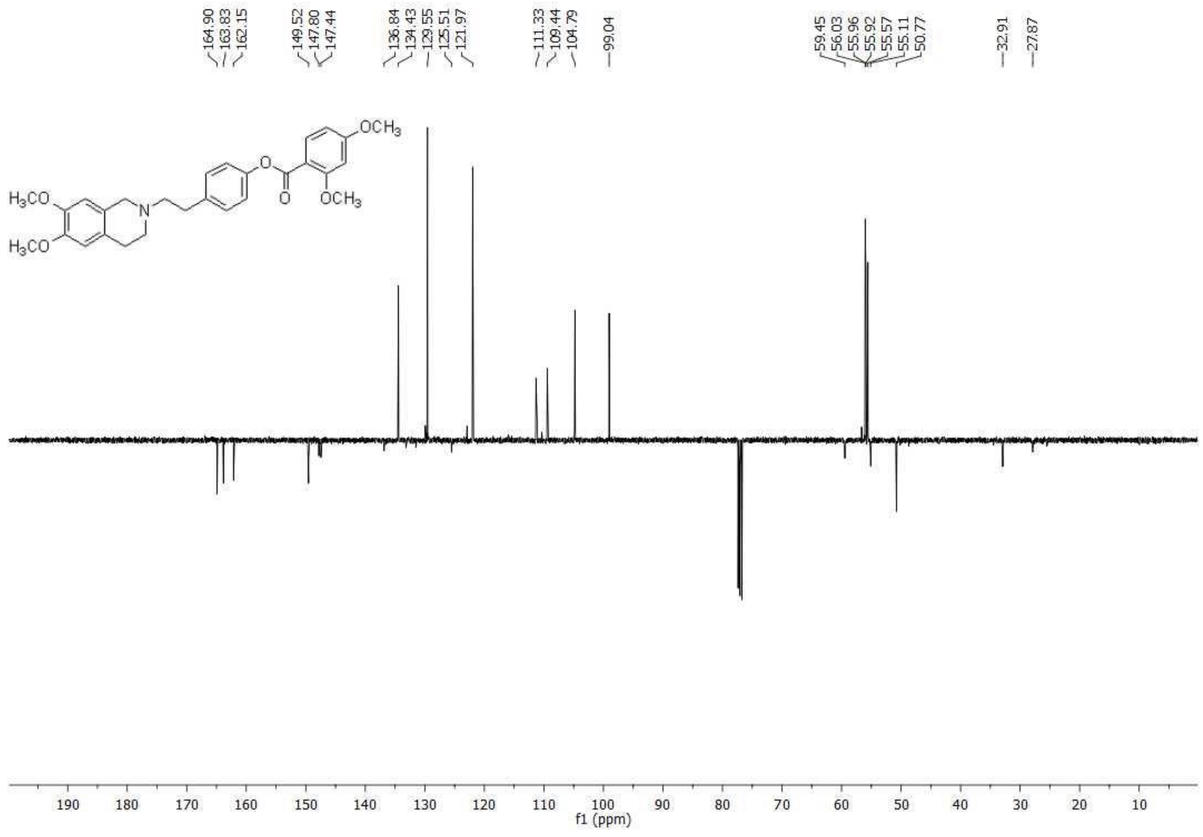

^{1}H-NMR and ^{13}C-APT-NMR spectra of compound **17**

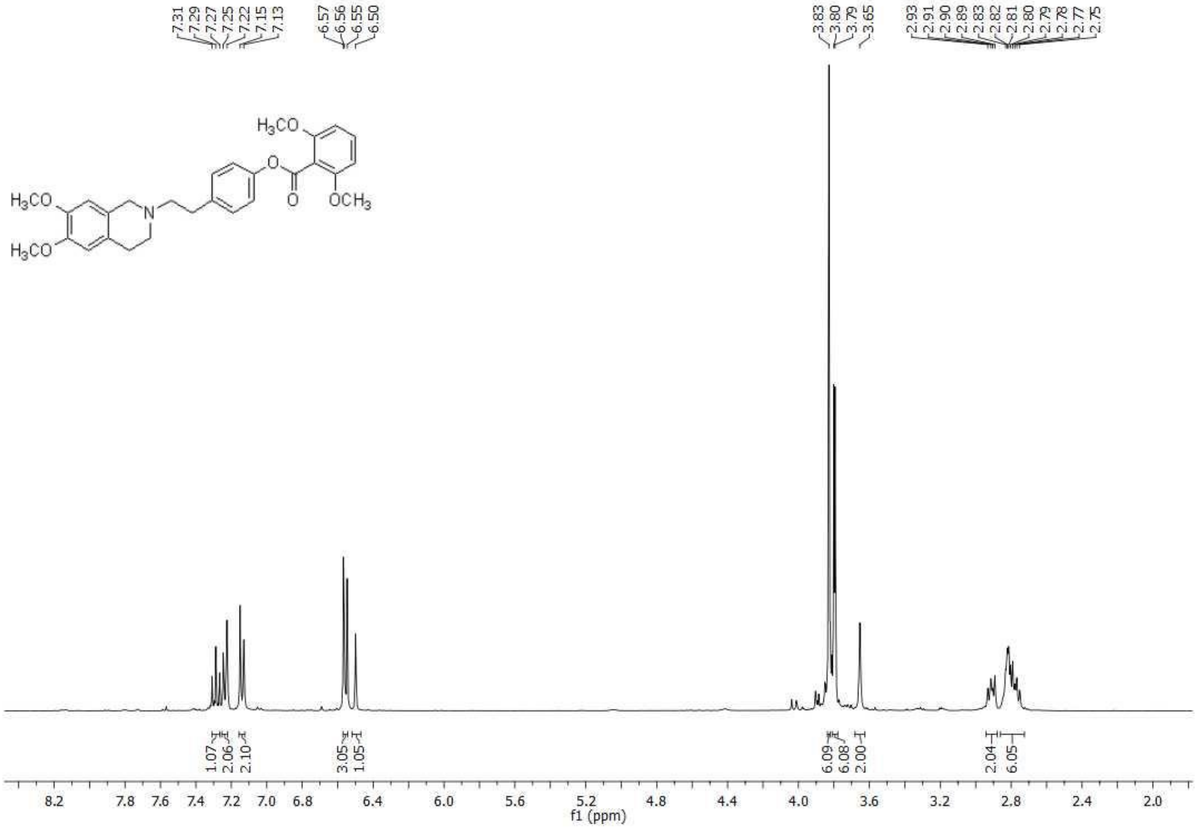

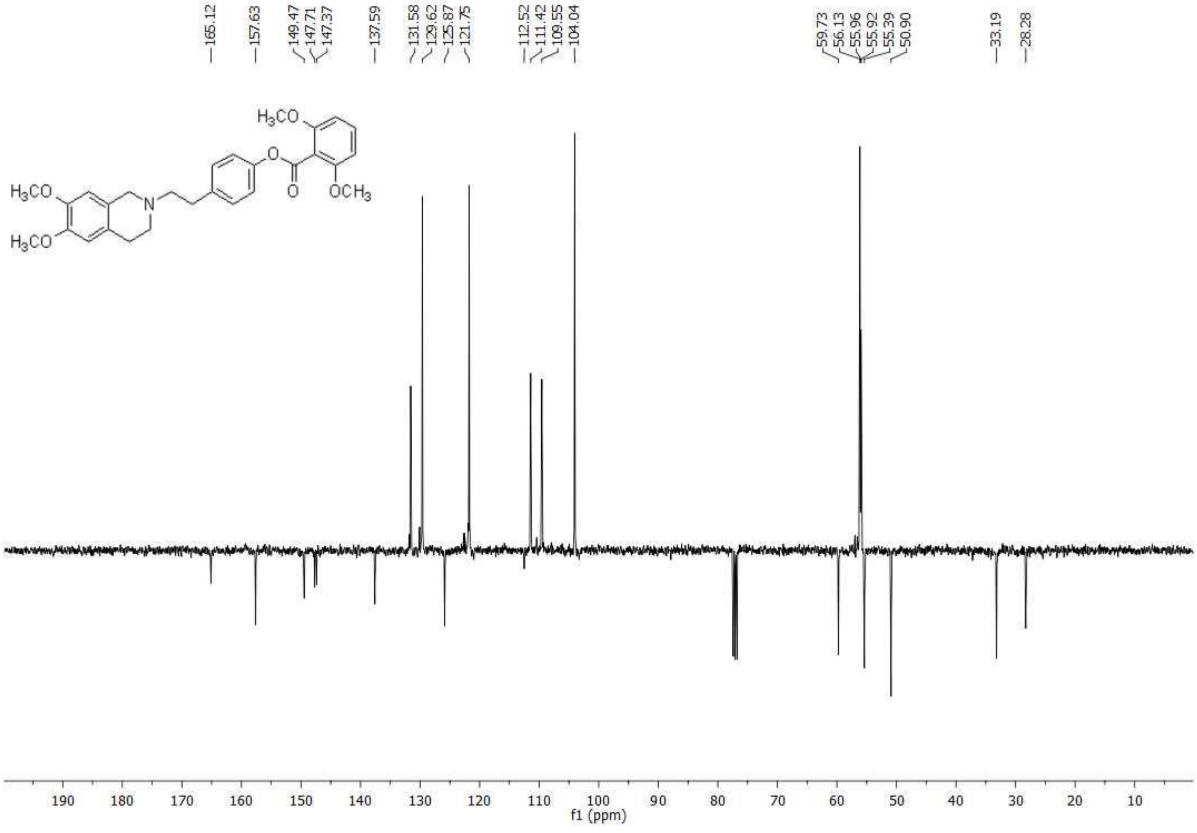

^{1}H-NMR and ^{13}C-APT-NMR spectra of compound **18**

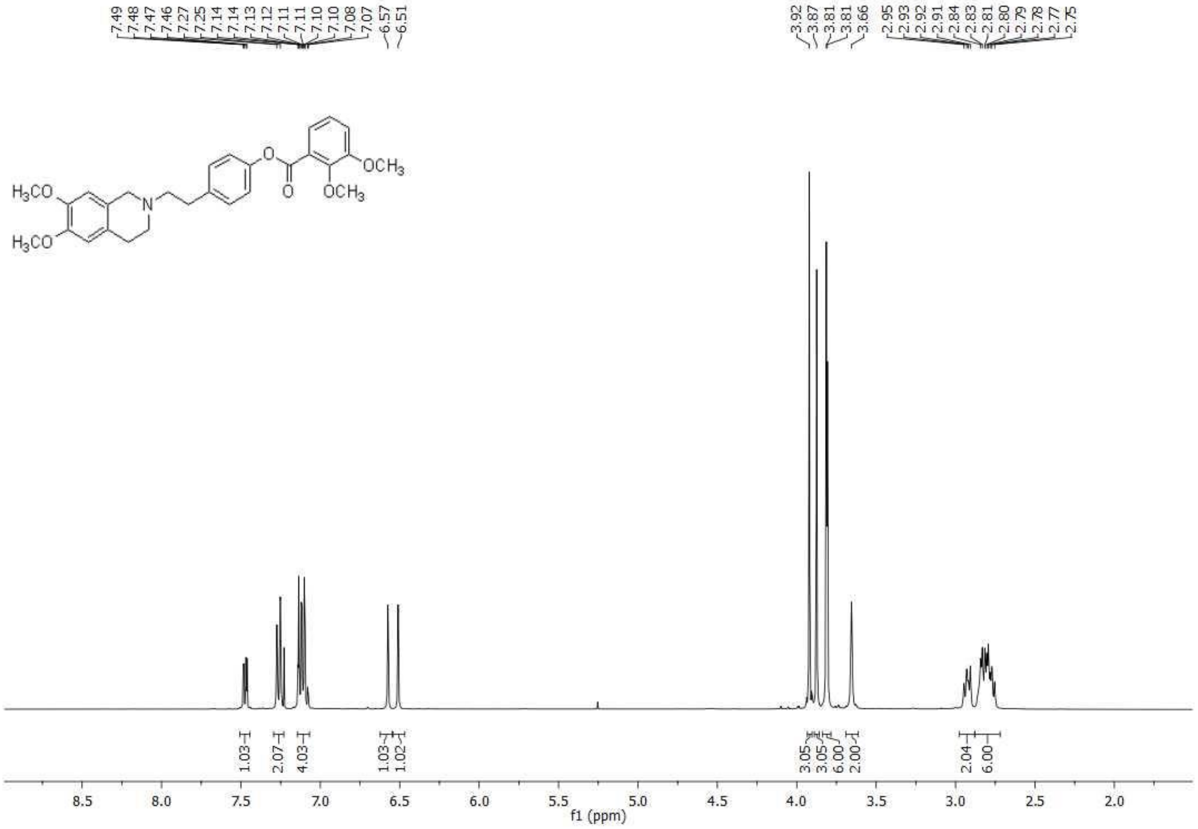

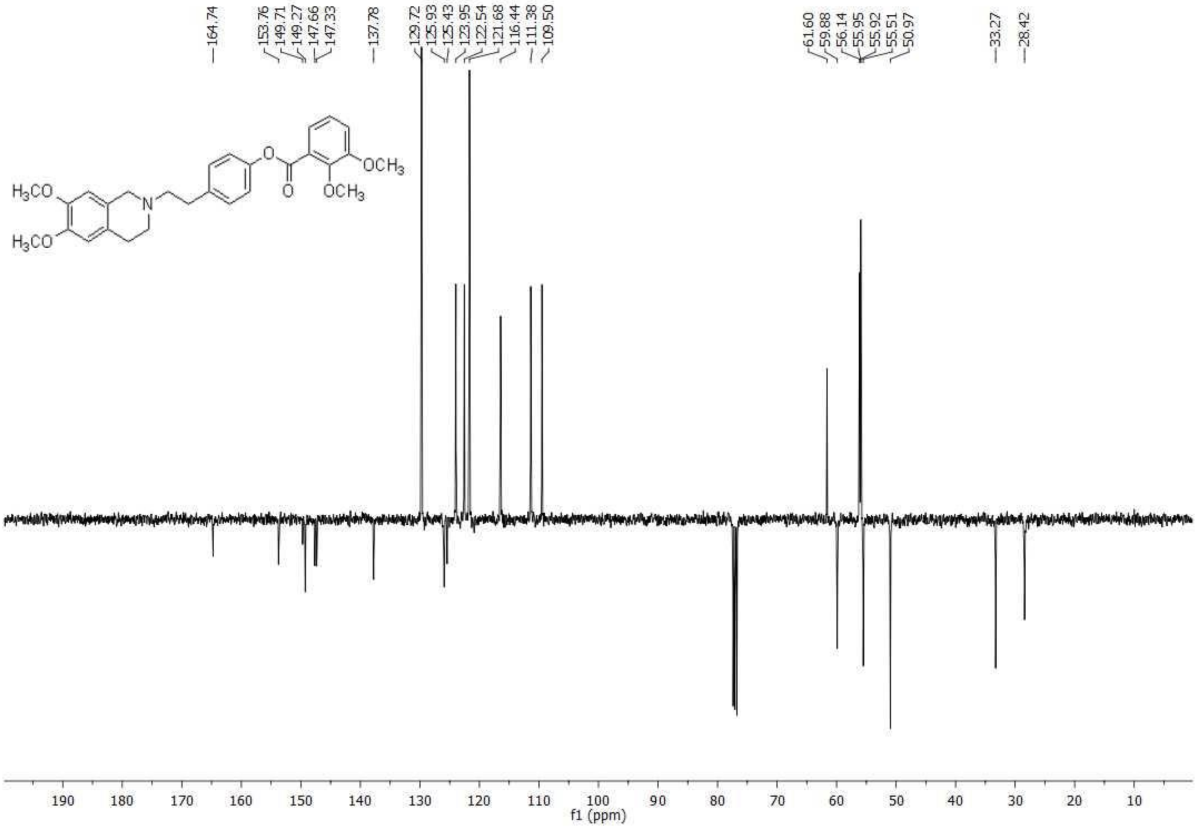

^{1}H-NMR and ^{13}C-APT-NMR spectra of compound **19**

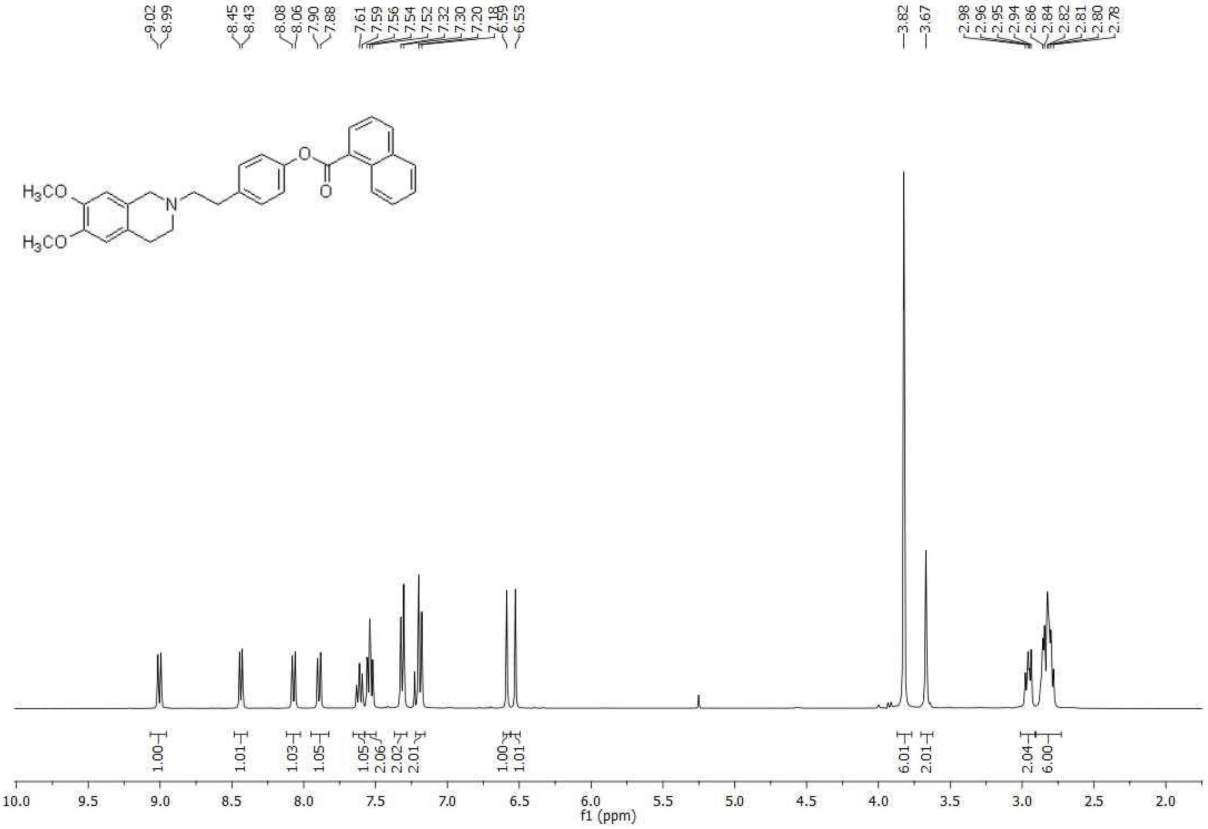

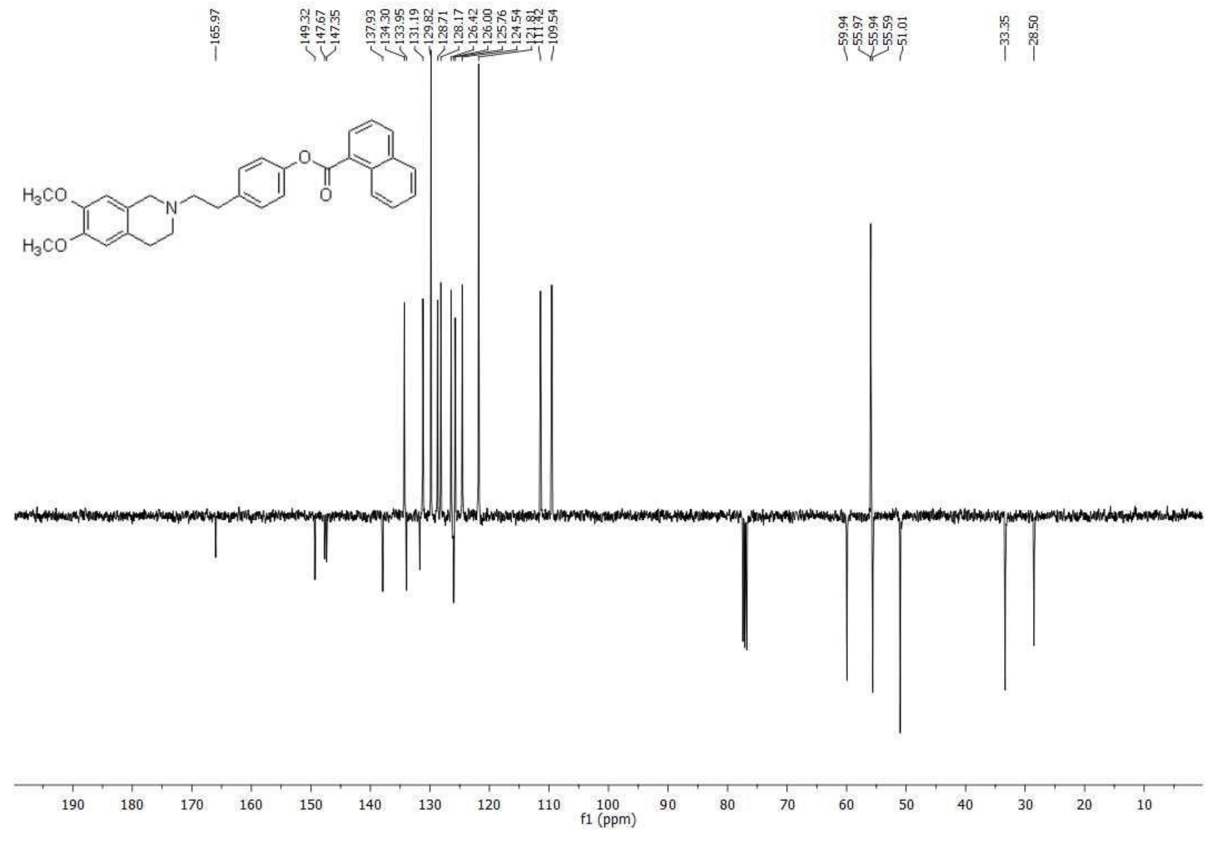

^{1}H-NMR and ^{13}C-APT-NMR spectra of compound **20**

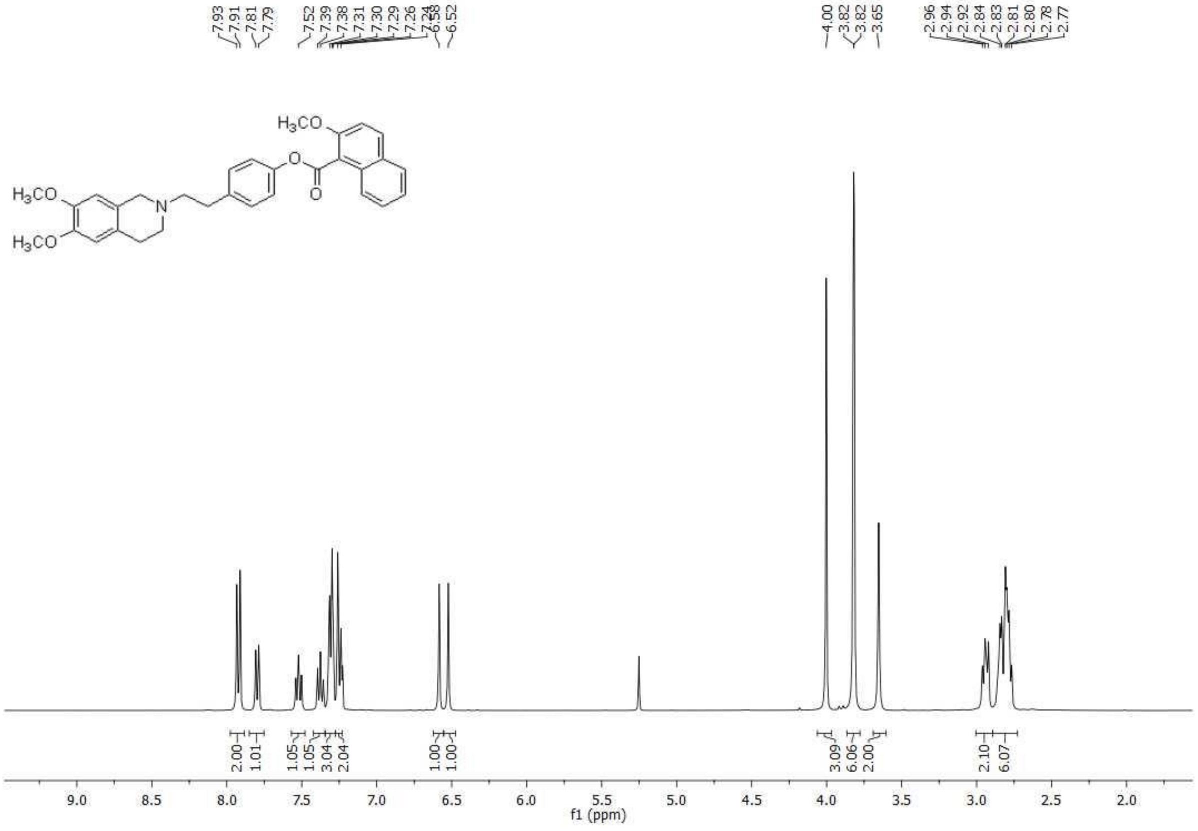

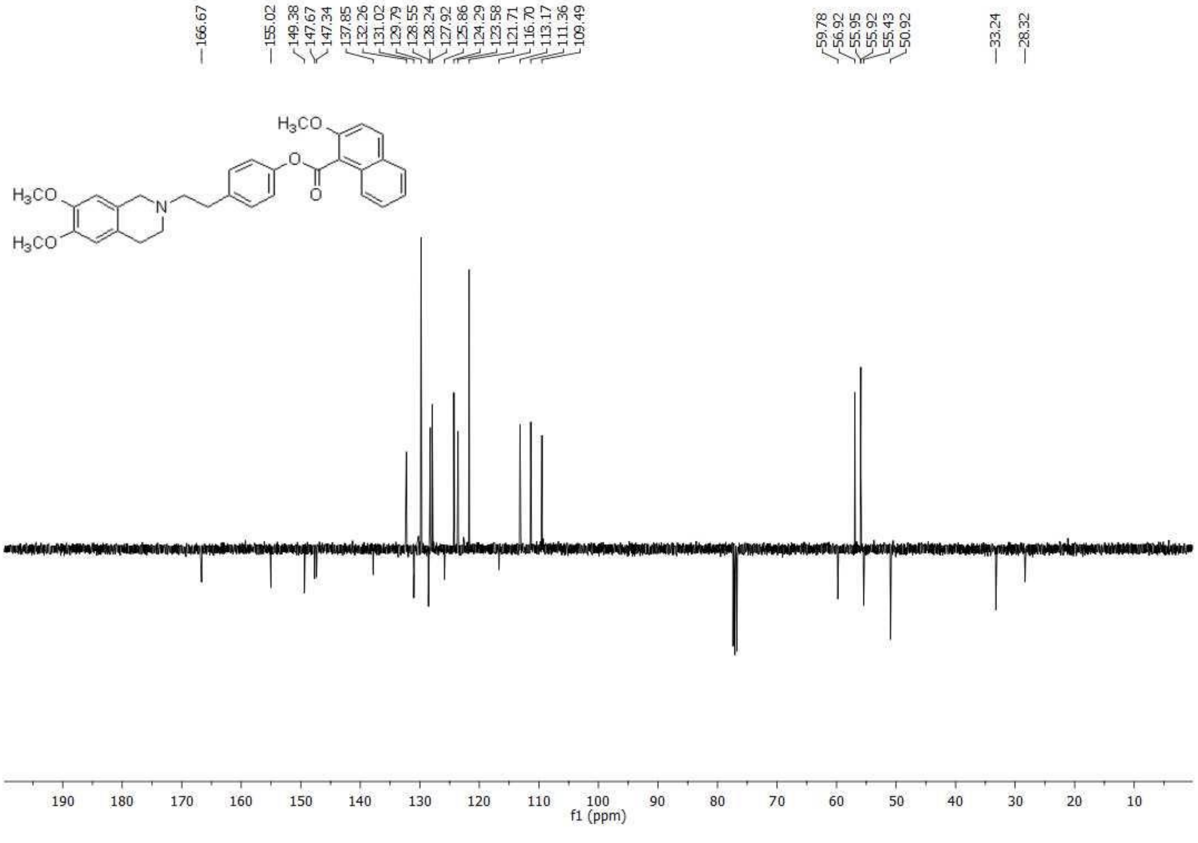

^{1}H-NMR and ^{13}C-APT-NMR spectra of compound **21**

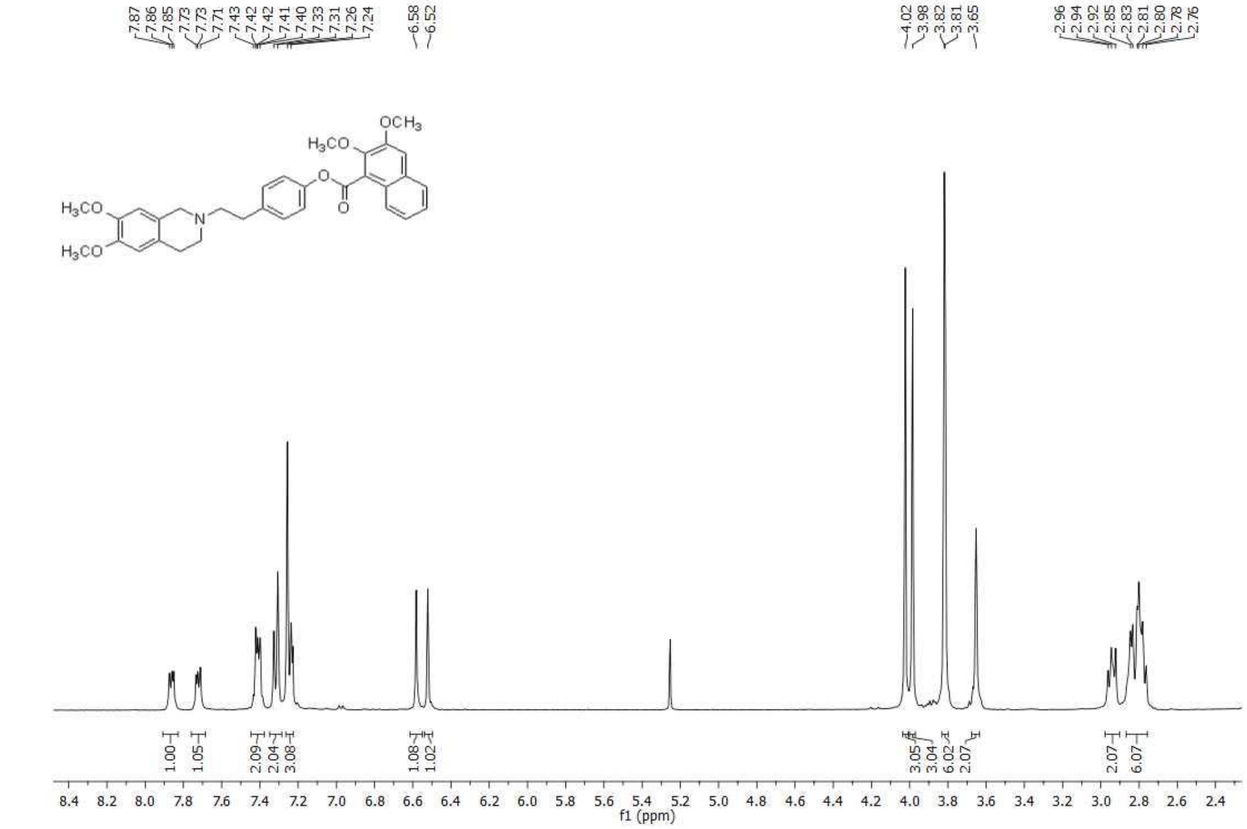

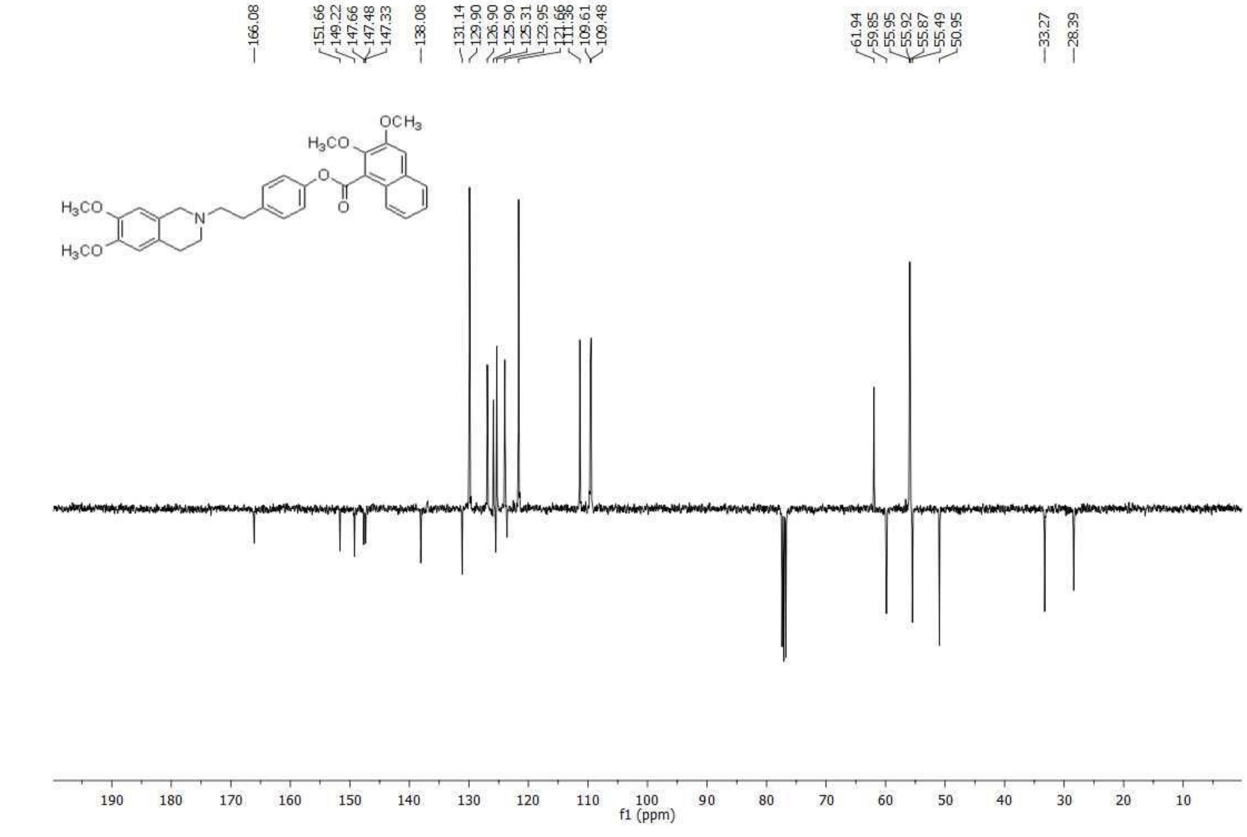

^{1}H-NMR and ^{13}C-APT-NMR spectra of compound **22**

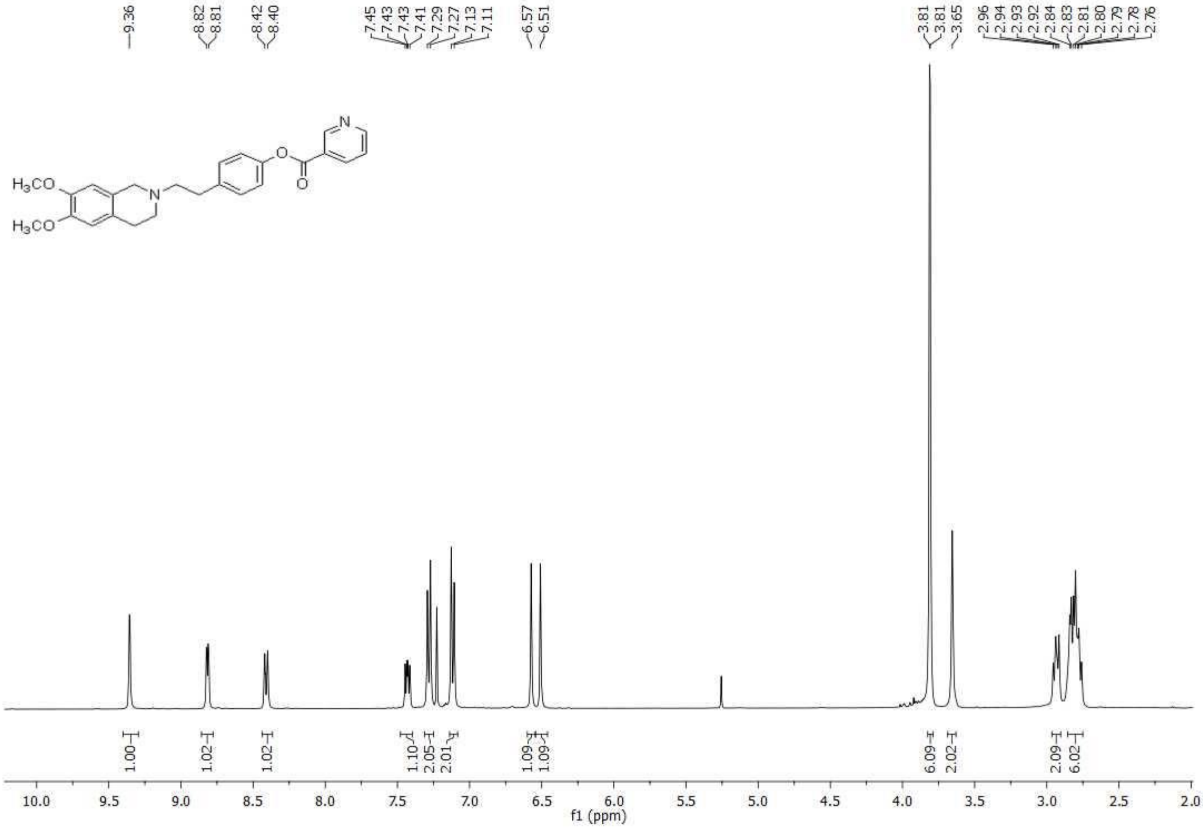

^{1}H-NMR and ^{13}C-APT-NMR spectra of compound **23**

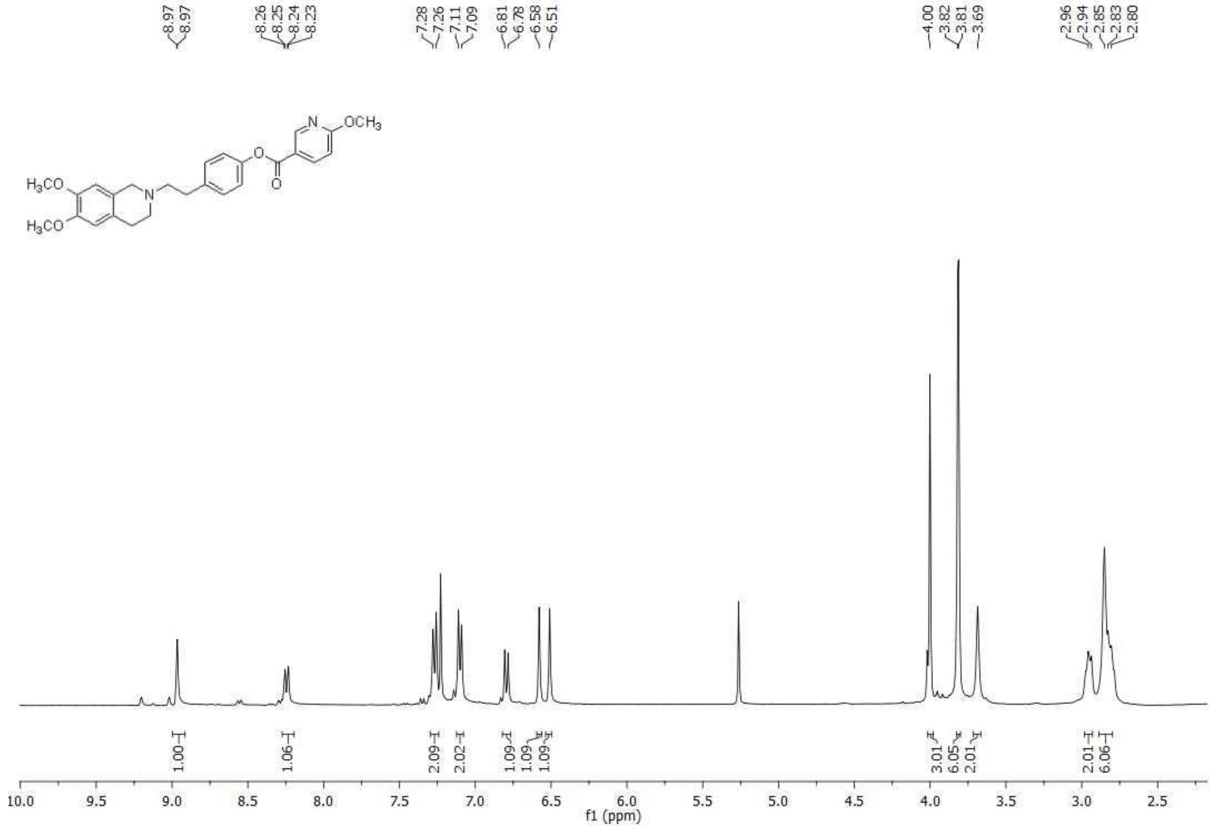

^{1}H-NMR and ^{13}C-APT-NMR spectra of compound **24**

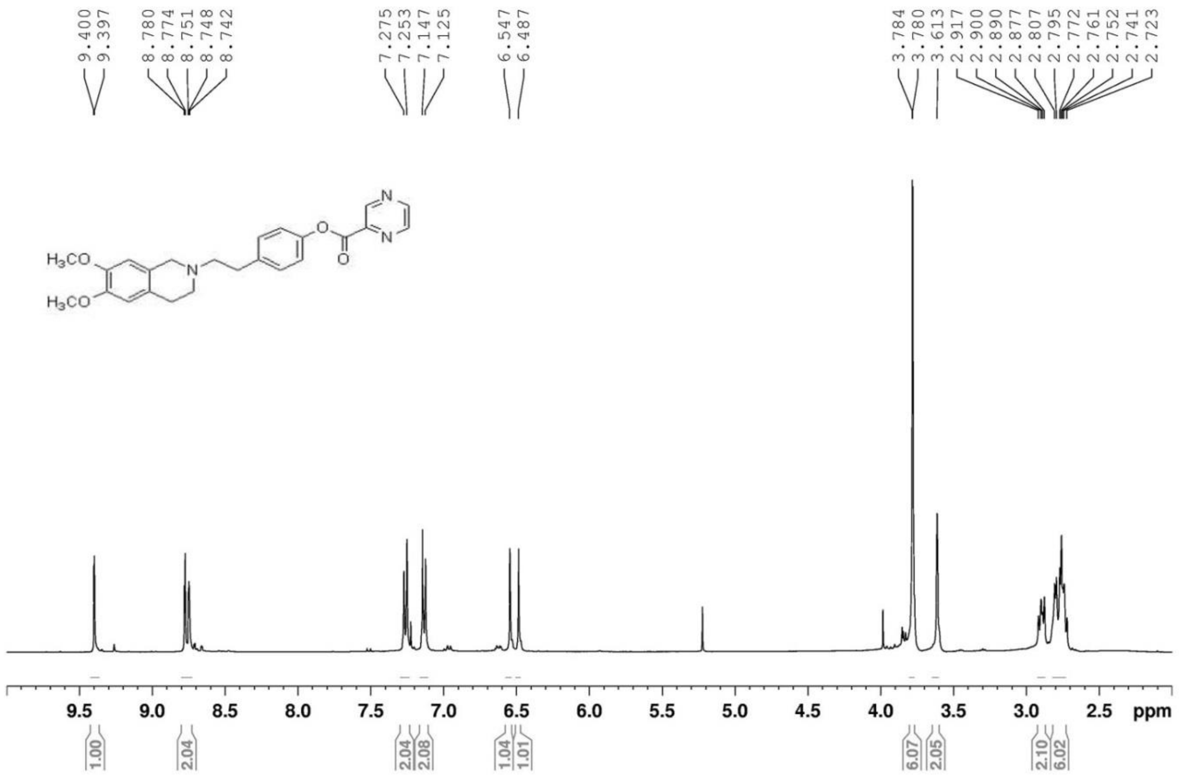

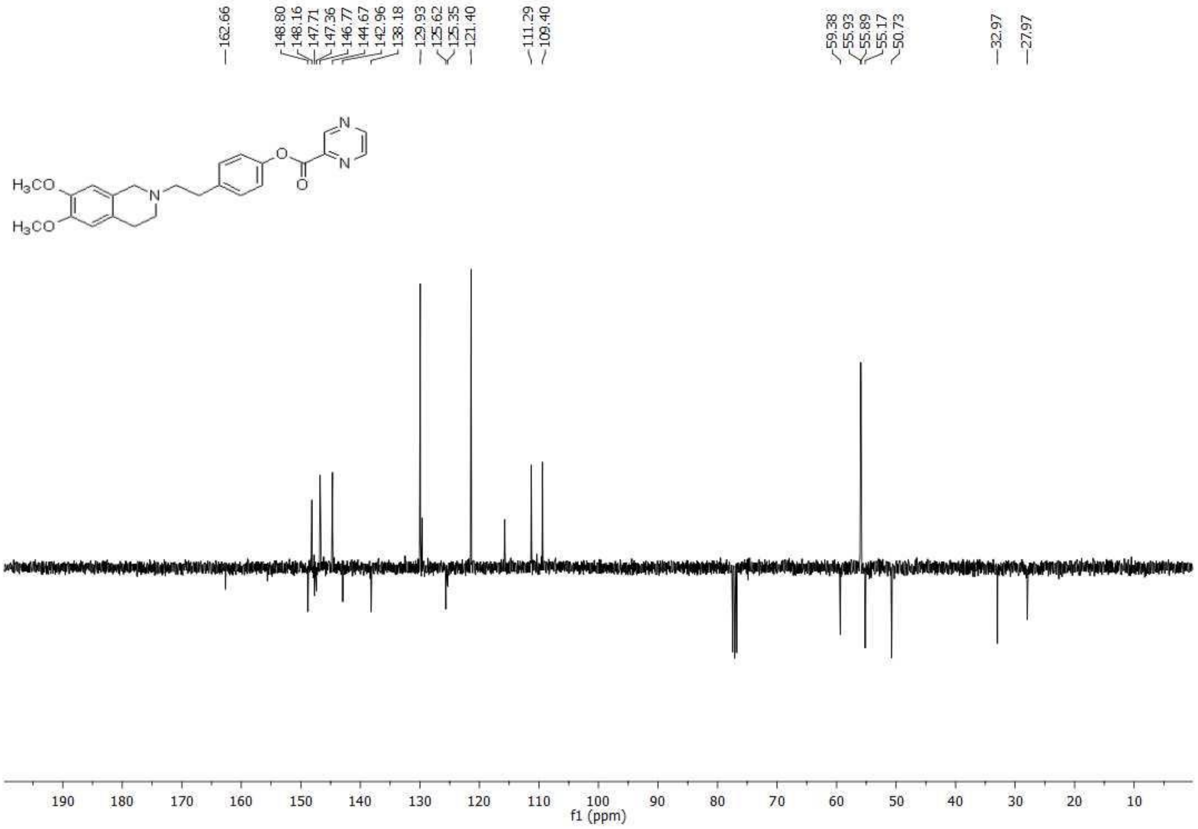

^{1}H-NMR and ^{13}C-APT-NMR spectra of compound **25**

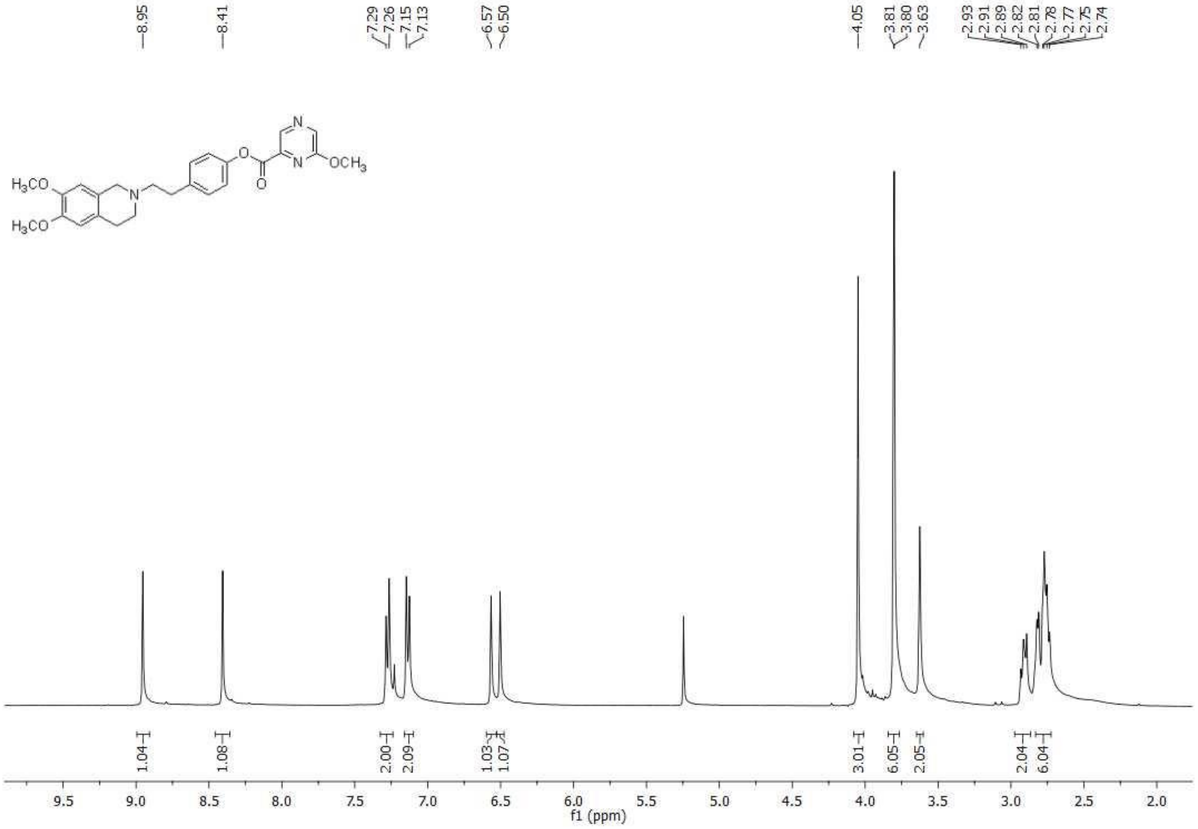

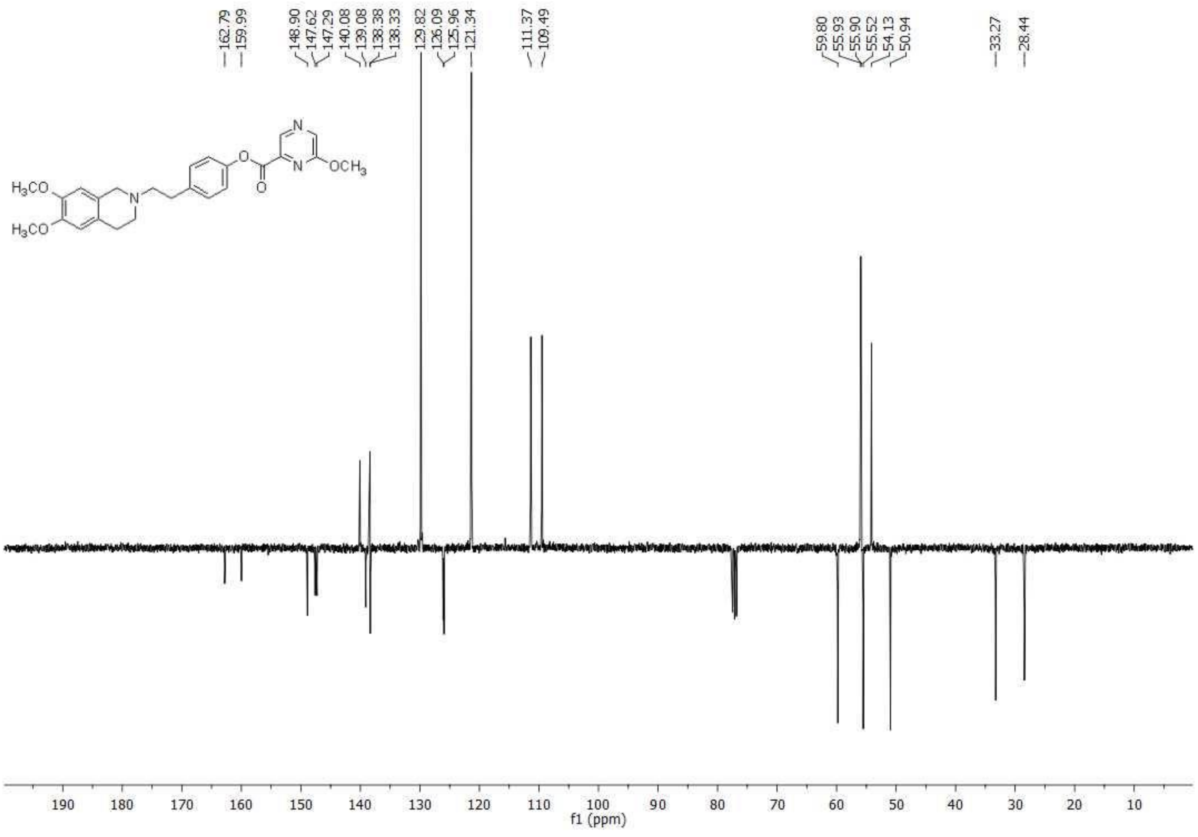

^{1}H-NMR and ^{13}C-APT-NMR spectra of compound **26**

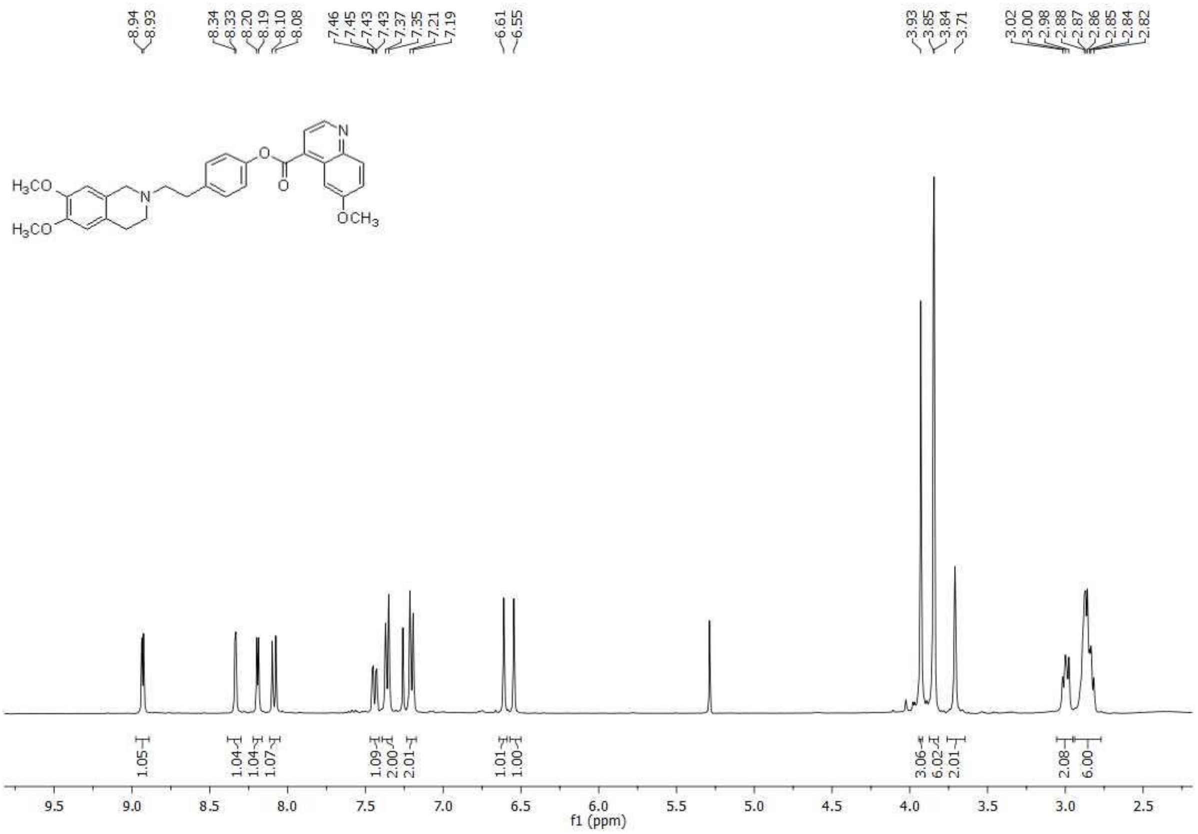

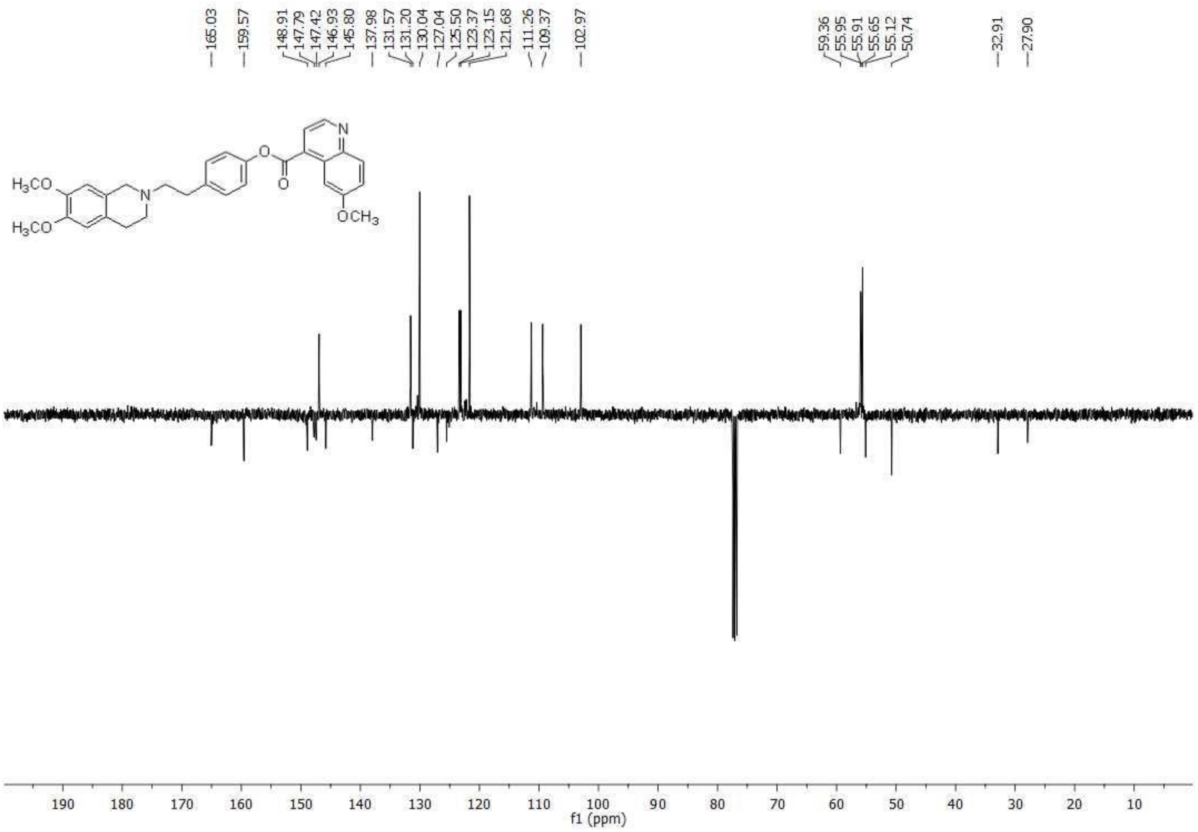

Chemical stability data

Instrumental

The LC-MS/MS analysis was carried out using a Varian 1200L triple quadrupole system (Palo Alto, CA, USA) equipped by two Prostar 210 pumps, a Prostar 410 autosampler and an Elettrospray Source (ESI) operating in positive ions mode. Raw-data were collected and processed by Varian Workstation Vers. 6.8 software. G-Therm 015 thermostatic oven was used to keep the samples at 37 °C during the degradation tests. Eppendorf microcentrifuge 5415D was employed to centrifuge plasma samples.

Standard solutions and calibration curves

Stock solutions of analytes and verapamil hydrochloride (ISTD) were prepared in acetonitrile at 1.0 mg mL^{-1} and stored at 4 °C. Working solutions of each analyte were freshly prepared by diluting stock solutions up to a concentration of 10 µM and 1 µM (working solution 1 and 2 respectively) in mQ water: acetonitrile 80:20 (v/v) solution. The ISTD working solution was prepared in acetonitrile at 60 ng mL^{-1} (ISTD solution).

A six levels calibration curve was prepared by adding proper volumes of working solution of each analyte to 300 µL of ISTD solution. The obtained solutions were dried under a gentle nitrogen stream and dissolved in 1.0 mL of 10 mM of formic acid in mQ water: acetonitrile 70:30 (v/v) solution. Final concentrations of calibration levels were: 0, 0.05, 0.10, 0.20, 0.50, 0.75 and 1.00 µM of analyte in the sample.

All calibration levels were analysed six times by the appropriate LC-MS/MS method.

LC-MS/MS method

The chromatographic parameters employed to analyse the samples were tuned to minimize the run time and were reported as follows:

- column, Pursuit C18 length = 30 mm; internal diameter = 2mm; particle size = 3 µm purchased from Agilent Technologies (Palo Alto, CA, USA)
- acidic mobile phase, composed by 5 mM of ammonium formate and 10mM of formic acid in mQ water: acetonitrile 90:10 (v/v) solution (solvent A), 5 mM of ammonium formate and 10mM of formic acid in mQ water: acetonitrile 10:90 (v/v) solution (solvent B).
- flow rate and the injection volume were 0.25 mL min^{-1} and 5 µL respectively.

The elution gradient is shown in Table S1.

The analyses were acquired in product ion scan, resonant excitation mode, parameters are reported in Table S2, using Nitrogen as collision gas.

Table S1: Elution gradient of mobile phase used for LC-MS/MS analyses

Time (min)	A (%)
0.00	90
4.00	10
7.00	10
7.01	90
10.00	90

Table S2: MRM parameters

Compounds	Precursor ion (m/z)	Quantitation ion (m/z) [CE (V)]	Qualification ion (m/z) [CE (V)]
**KEE**	283	209 [15]	209 [30]
**1**	507	195 [35]	314 [30]
**2**	447	135 [45]	254 [30]
**3**	477	165 [45]	284 [30]
**4**	477	165 [40]	284 [30]
**5**	477	165 [45]	284 [30]
**6**	467	155 [50]	274 [30]
**7**	497	185 [40]	304 [30]
**8**	527	215 [45]	334 [30]
**9**	418	225 [30]	254 [25]
**10**	448	136 [50]	255 [30]
**11**	419	226 [40]	255 [30]
**12**	449	256 [35]	285 [35]
**13**	498	186 [50]	334 [35]
**14**	508	195 [40]	179 [35]
**15**	448	135 [35]	179 [30]
**16**	478	165 [40]	179 [35]
**17**	478	165 [40]	179 [30]
**18**	478	165 [40]	179 [35]
**19**	468	155 [40]	179 [30]
**20**	498	185 [40]	179 [35]
**21**	528	215 [40]	179 [40]
**22**	419	255 [30]	179 [30]
**23**	449	136 [45]	285 [35]
**24**	420	256 [30]	227 [40]
**25**	450	179 [30]	286 [35]
**26**	499	186 [50]	335 [35]

Linearity and LOD

Calibration curves of analytes were obtained by plotting the peak area ratios (PAR), between quantitation ions of analyte and ISTD, versus the nominal concentration of the calibration solution. A linear regression analysis was applied to obtain the best fitting function between the calibration points.

The precision was evaluated through the relative standard deviation (RSD%) of the quantitative data of the replicate analysis of highest level of calibration curves.

In order to obtain reliable LOD values, the standard deviation of response and slope approach was employed. The estimated standard deviations of responses were obtained by the standard deviation

of y-intercepts (SDY-I) of regression lines. The obtained linear regressions, the linearity coefficients, precision and the estimated LOD values for each analyte are reported in Table S3.

Table S3: Linear regressions data, linearity coefficients, precision and LOD values for each analyte

Compounds	Slope (PAR/μM)	Intercept (PAR)	R^2	Precision (RSD)	LOD (μM)
**1**	1.88	0.008	0.998	1.4%	0.05
**2**	2.15	0.002	0.999	5.0%	0.07
**3**	2.02	-0.010	0.997	0.8%	0.08
**4**	2.09	0.005	0.999	5.1%	0.09
**5**	4.11	-0.003	0.996	1.2%	0.05
**6**	1.83	-0.005	0.997	2.6%	0.05
**7**	3.92	0.007	0.999	1.1%	0.06
**8**	2.52	-0.011	0.999	1.4%	0.05
**9**	0.26	0.012	0.999	2.8%	0.09
**10**	0.46	0.005	0.999	6.5%	0.05
**11**	1.56	0.009	0.999	1.6%	0.04
**12**	0.75	0.015	0.998	2.0%	0.06
**13**	0.65	0.013	0.999	3.0%	0.05
**14**	4.77	0.007	0.999	0.5%	0.08
**15**	2.73	-0.004	0.999	4.5%	0.05
**16**	2.06	-0.008	0.999	1.9%	0.05
**17**	3.67	-0.011	0.999	0.3%	0.07
**18**	4.85	0.007	0.999	1.9%	0.08
**19**	3.15	0.001	0.998	0.8%	0.06
**20**	3.93	-0.005	0.996	2.0%	0.05
**21**	2.53	0.003	0.999	2.8%	0.07
**22**	0.33	0.007	0.999	4.7%	0.08
**23**	0.91	-0.012	0.997	1.5%	0.09
**24**	0.13	-0.009	0.999	7.0%	0.07
**25**	0.55	0.009	0.998	1.0%	0.05
**26**	0.65	0.002	0.999	2.9%	0.04

Solution stability profiles

The solution stability profiles in PBS and human plasma were obtained by monitoring the variation of analyte concentration at different incubation times. They are reported in Figures S1-S26.

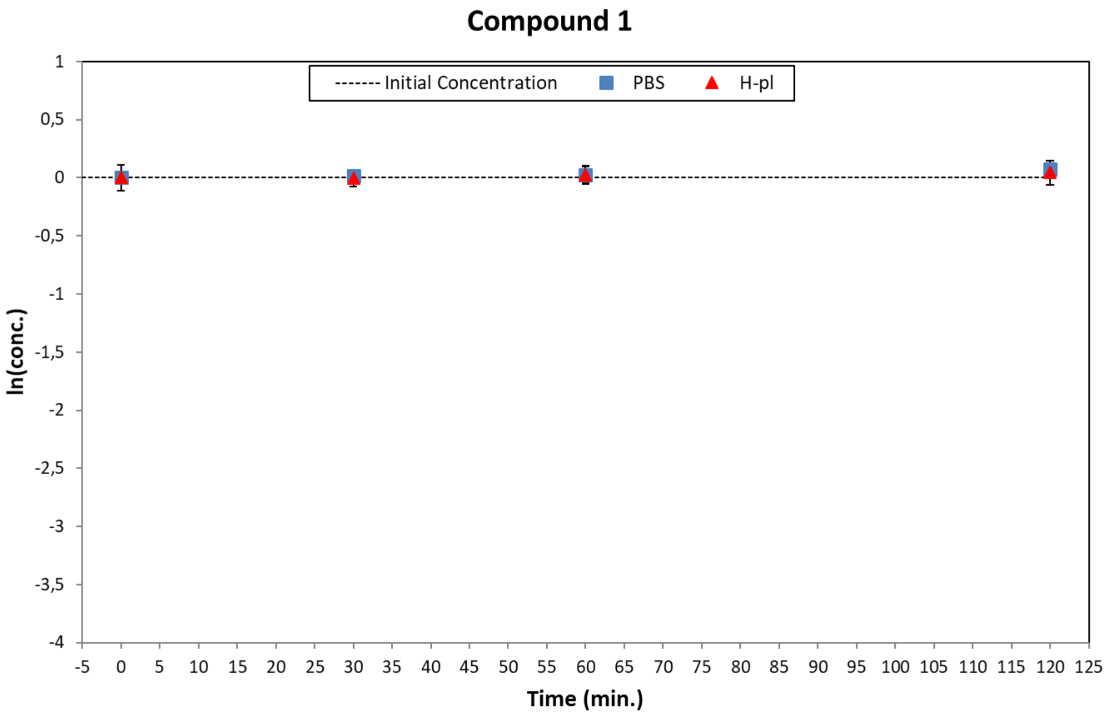

Figure S1: Degradation plots of **1** in PBS (blue square) and human plasma (red triangle).

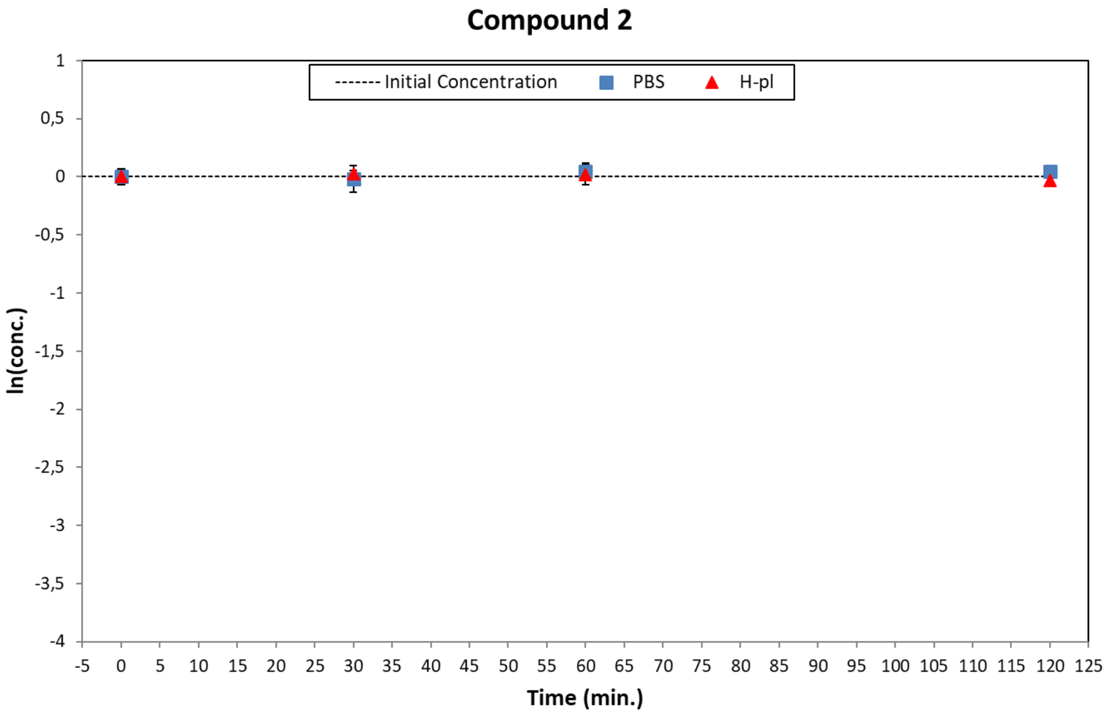

Figure S2: Degradation plots of **2** in PBS (blue square) and human plasma (red triangle).

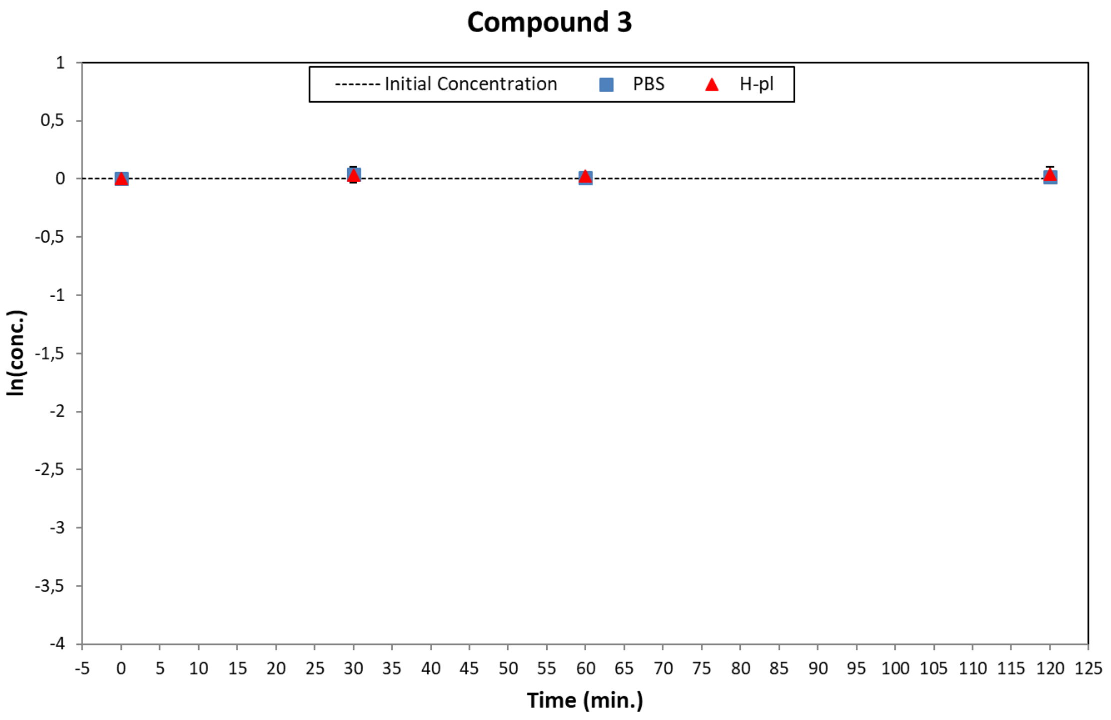

Figure S3: Degradation plots of **3** in PBS (blue square) and human plasma (red triangle).

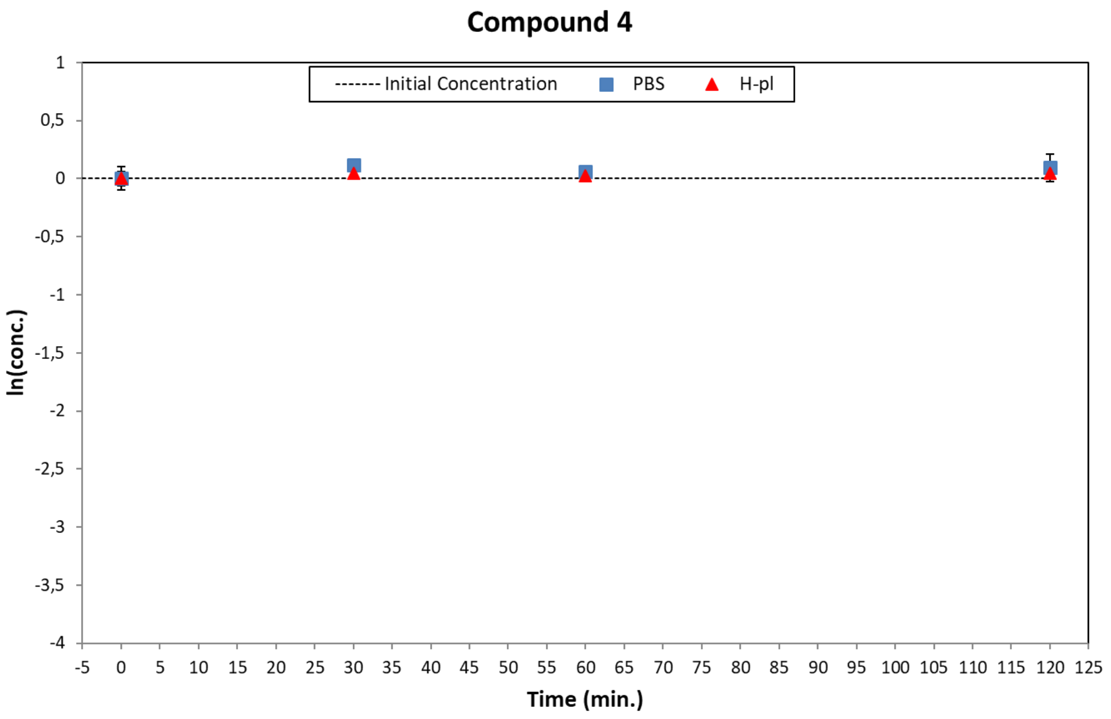

Figure S4: Degradation plots of **4** in PBS (blue square) and human plasma (red triangle).

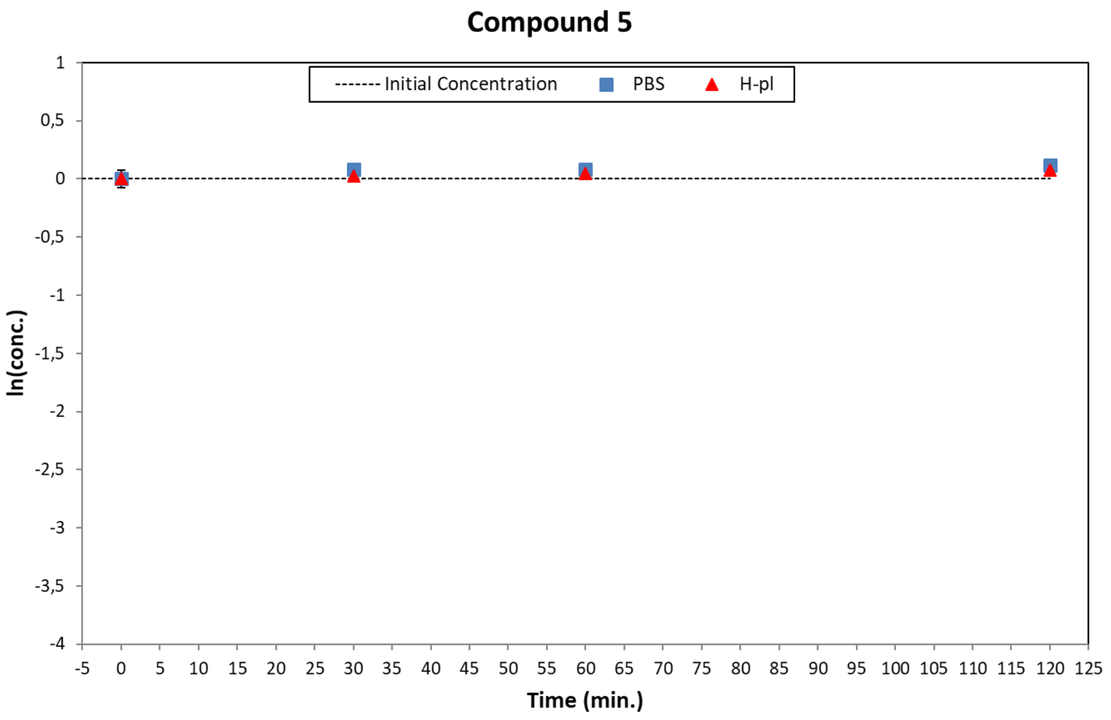

Figure S5: Degradation plots of **5** in PBS (blue square) and human plasma (red triangle).

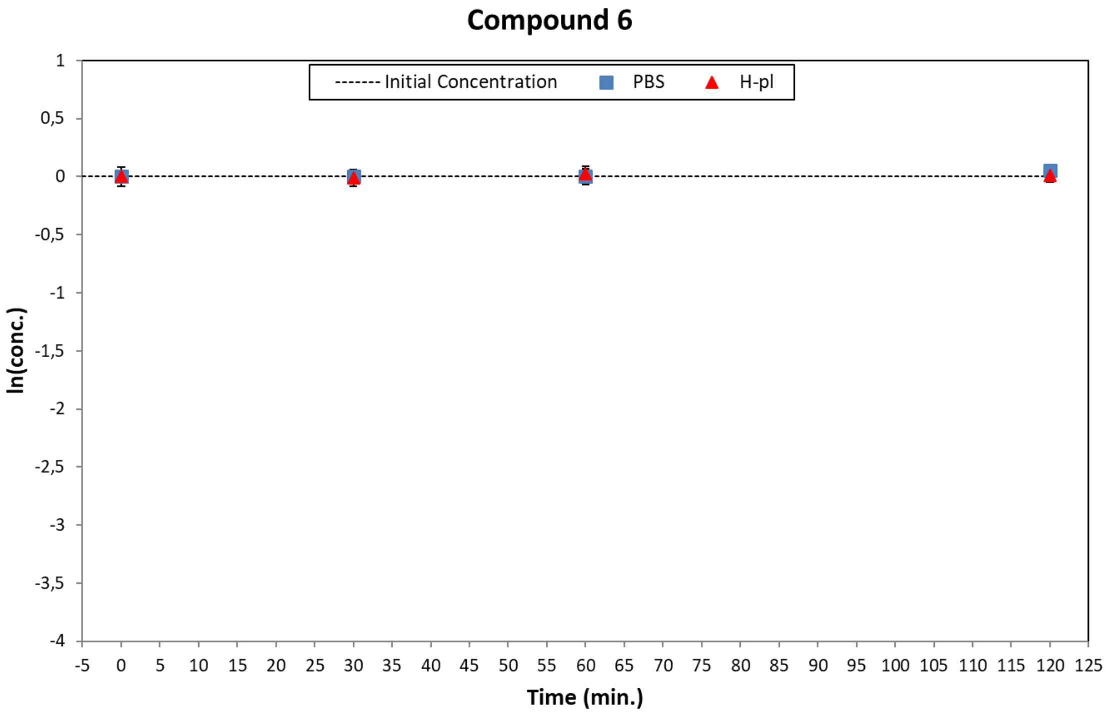

Figure S6: Degradation plots of **6** in PBS (blue square) and human plasma (red triangle).

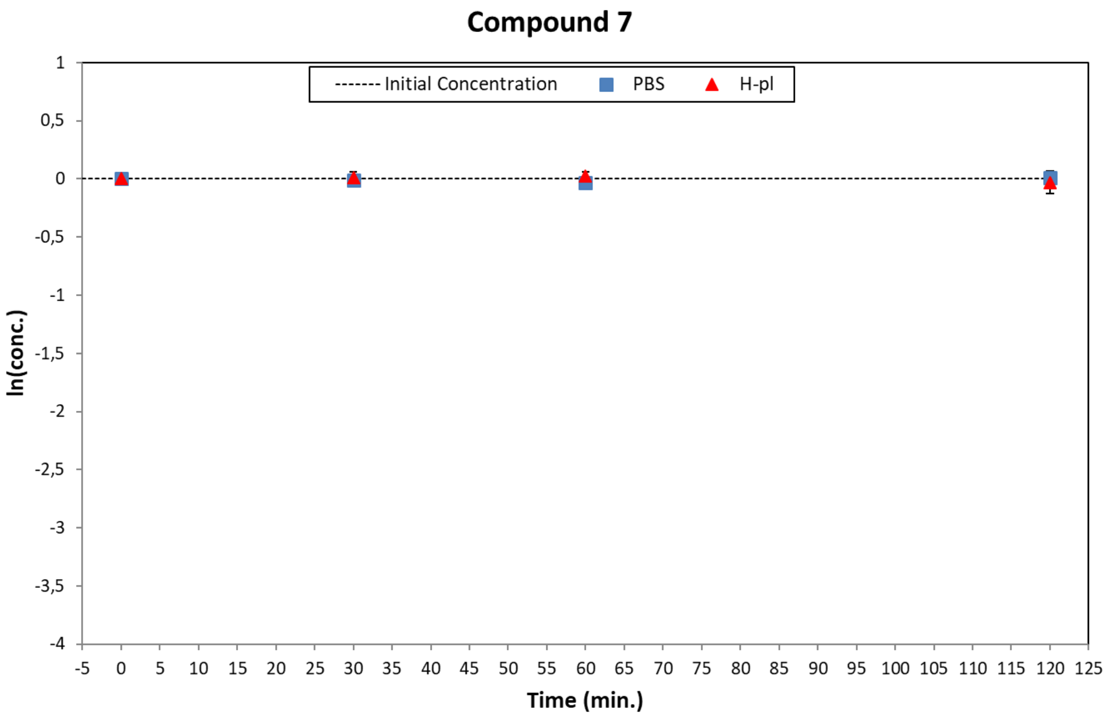

Figure S7: Degradation plots of **7** in PBS (blue square) and human plasma (red triangle).

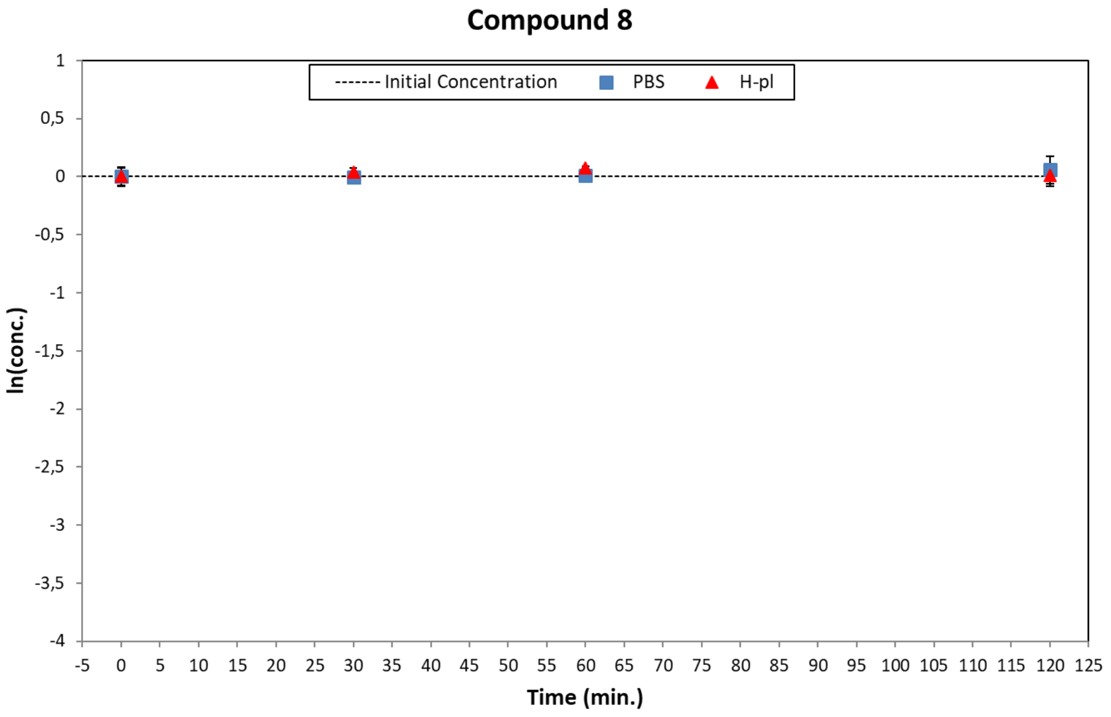

Figure S8: Degradation plots of **8** in PBS (blue square) and human plasma (red triangle).

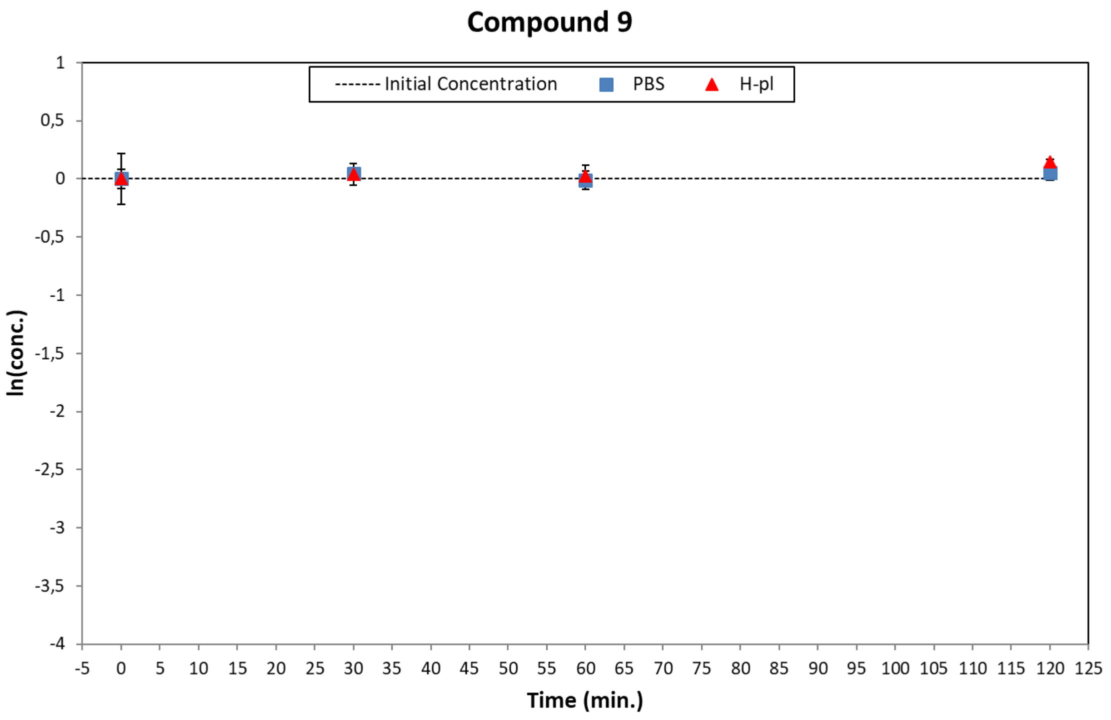

Figure S9: Degradation plots of **9** in PBS (blue square) and human plasma (red triangle).

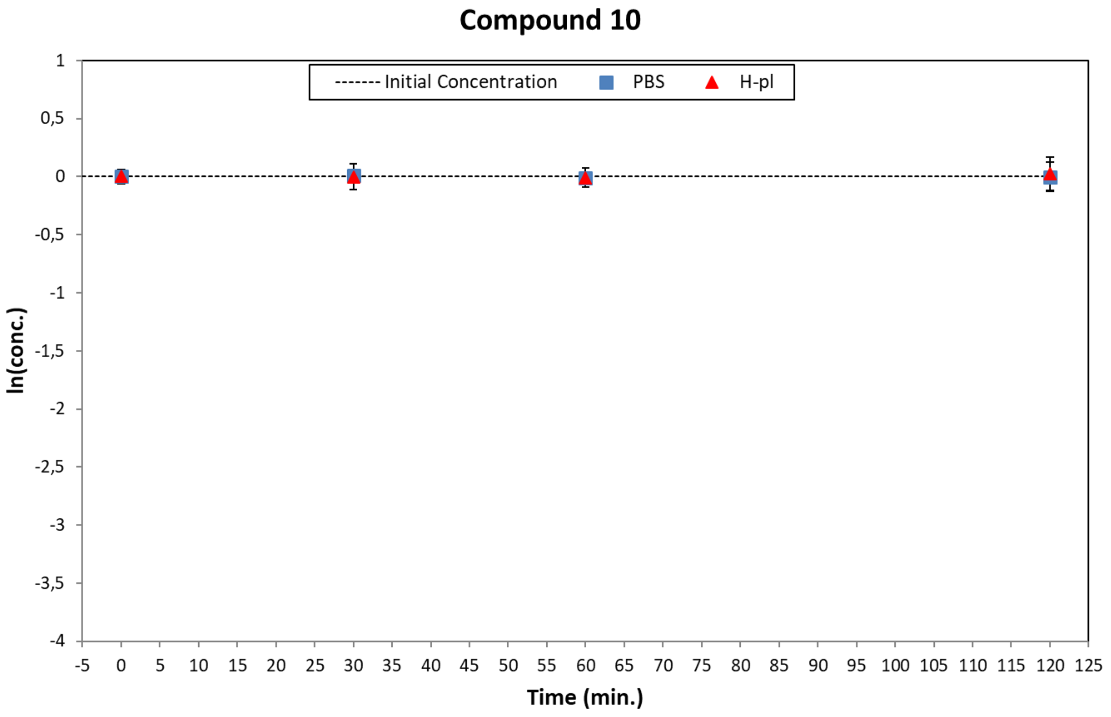

Figure S10: Degradation plots of **10** in PBS (blue square) and human plasma (red triangle).

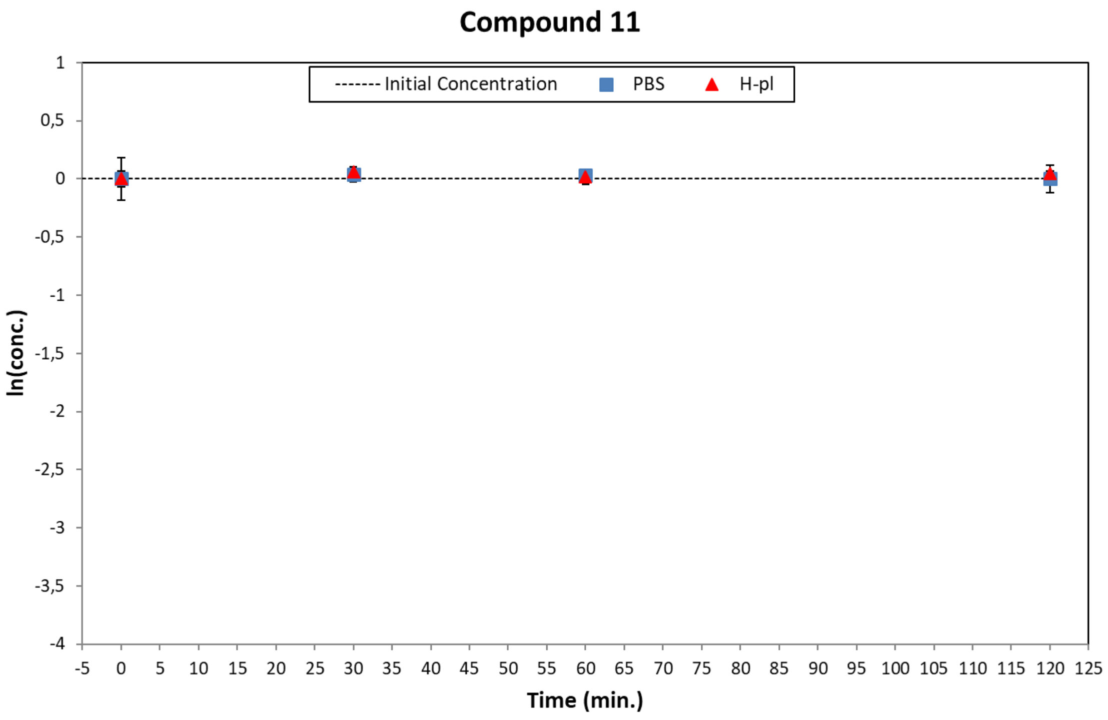

Figure S11: Degradation plots of **11** in PBS (blue square) and human plasma (red triangle).

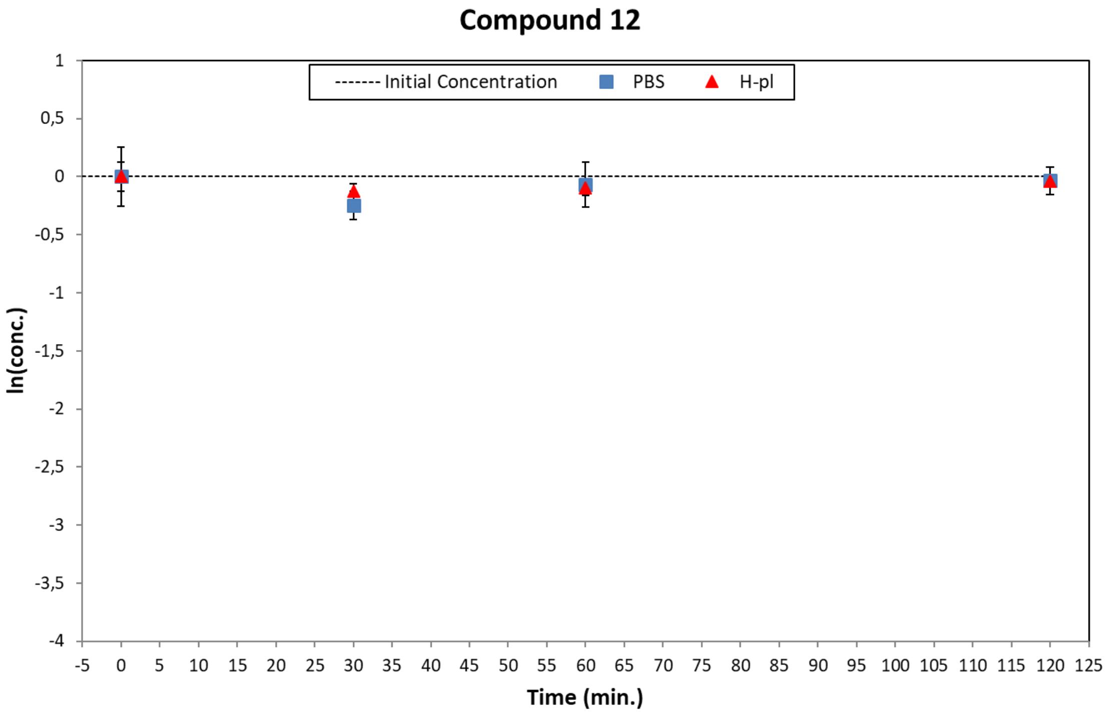

Figure S12: Degradation plots of **12** in PBS (blue square) and human plasma (red triangle).

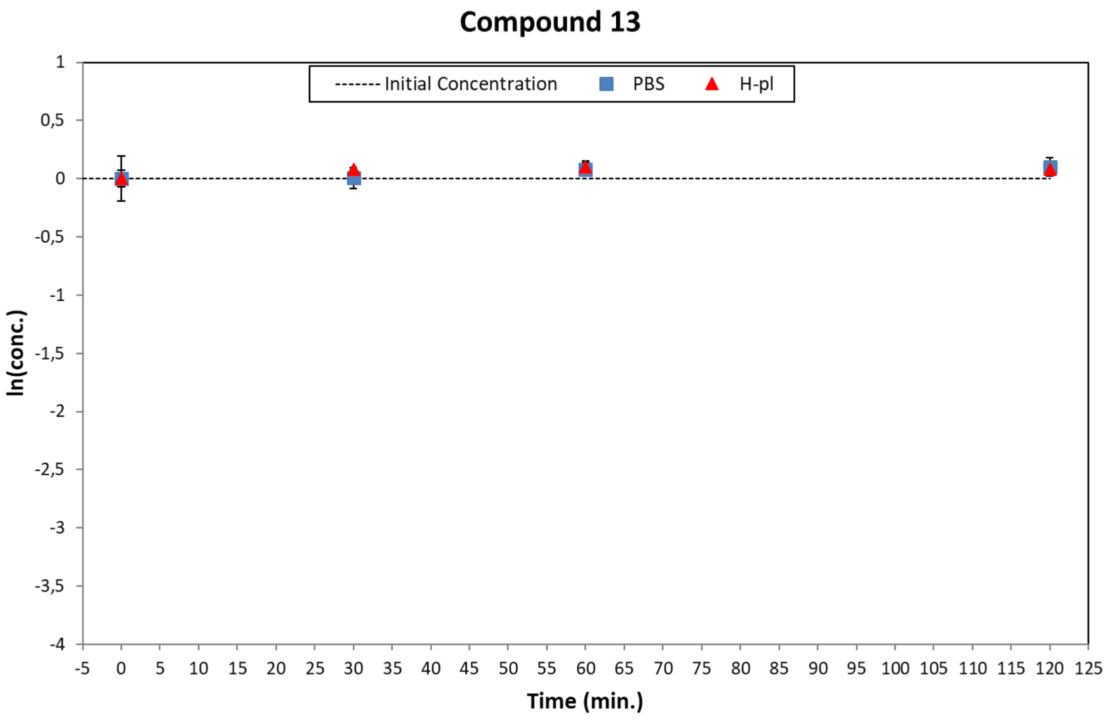

Figure S13: Degradation plots of **13** in PBS (blue square) and human plasma (red triangle).

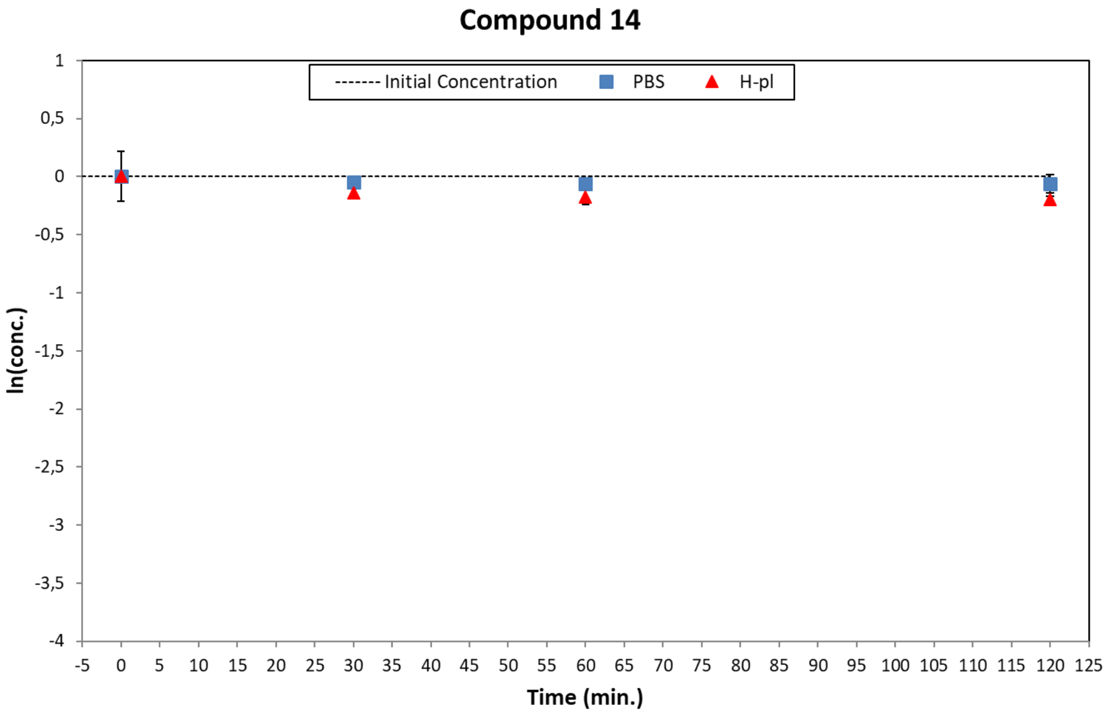

Figure S14: Degradation plots of **14** in PBS (blue square) and human plasma (red triangle).

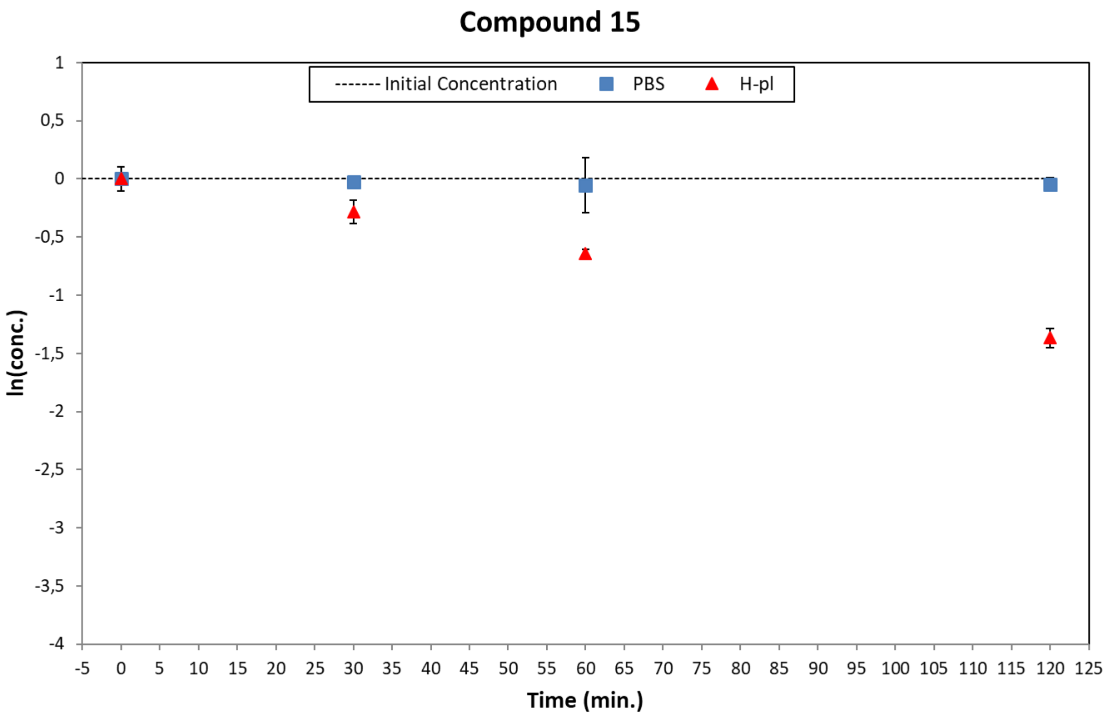

Figure S15: Degradation plots of **15** in PBS (blue square) and human plasma (red triangle).

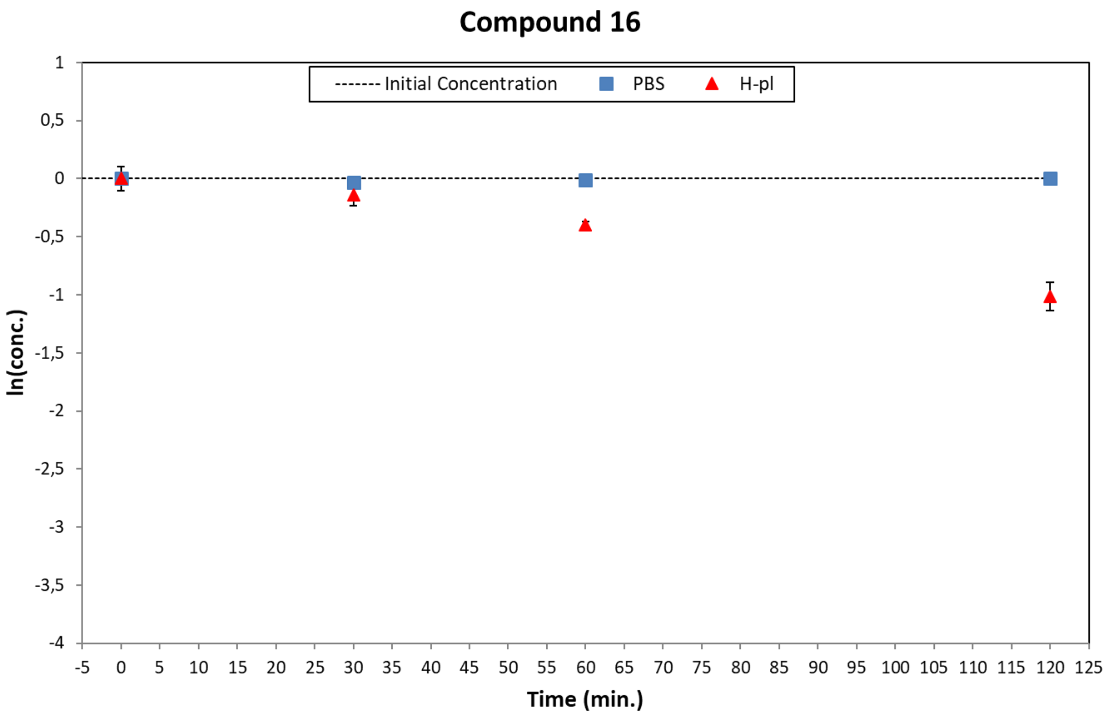

Figure S16: Degradation plots of **16** in PBS (blue square) and human plasma (red triangle).

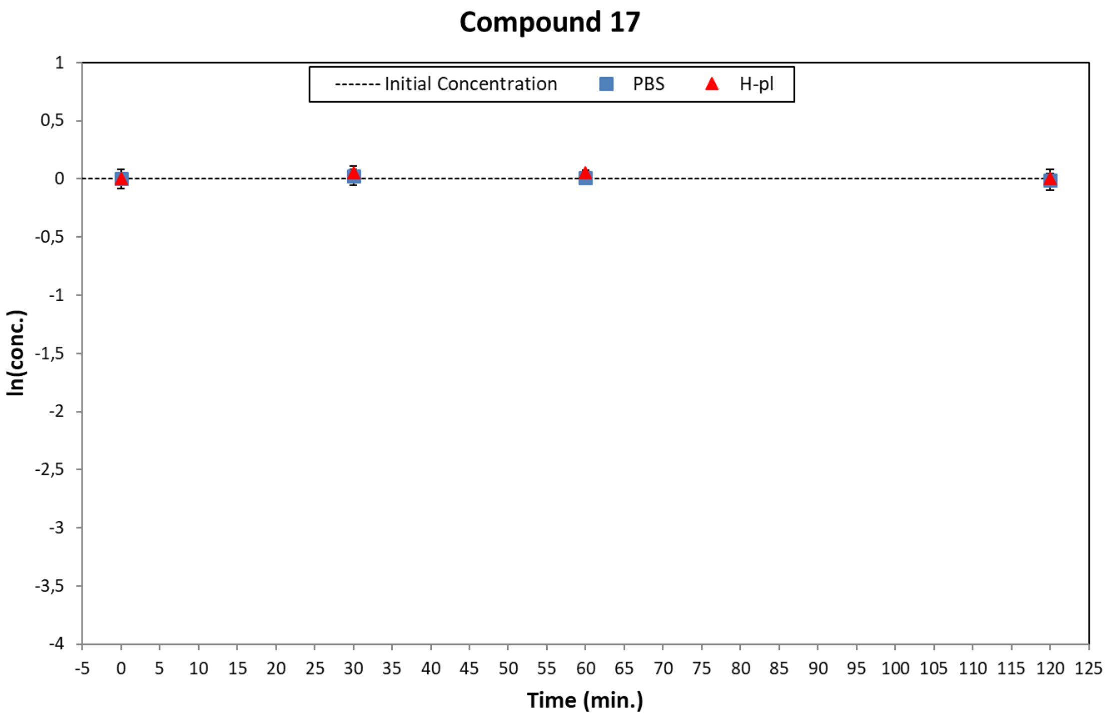

Figure S17: Degradation plots of **17** in PBS (blue square) and human plasma (red triangle).

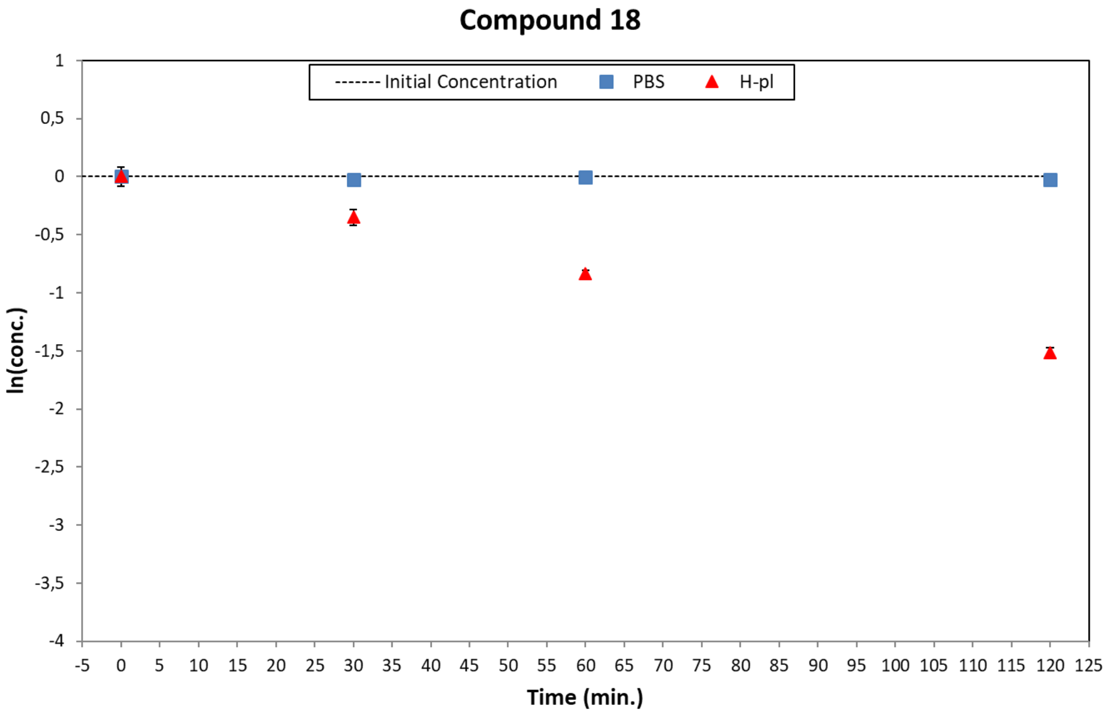

Figure S18: Degradation plots of **18** in PBS (blue square) and human plasma (red triangle).

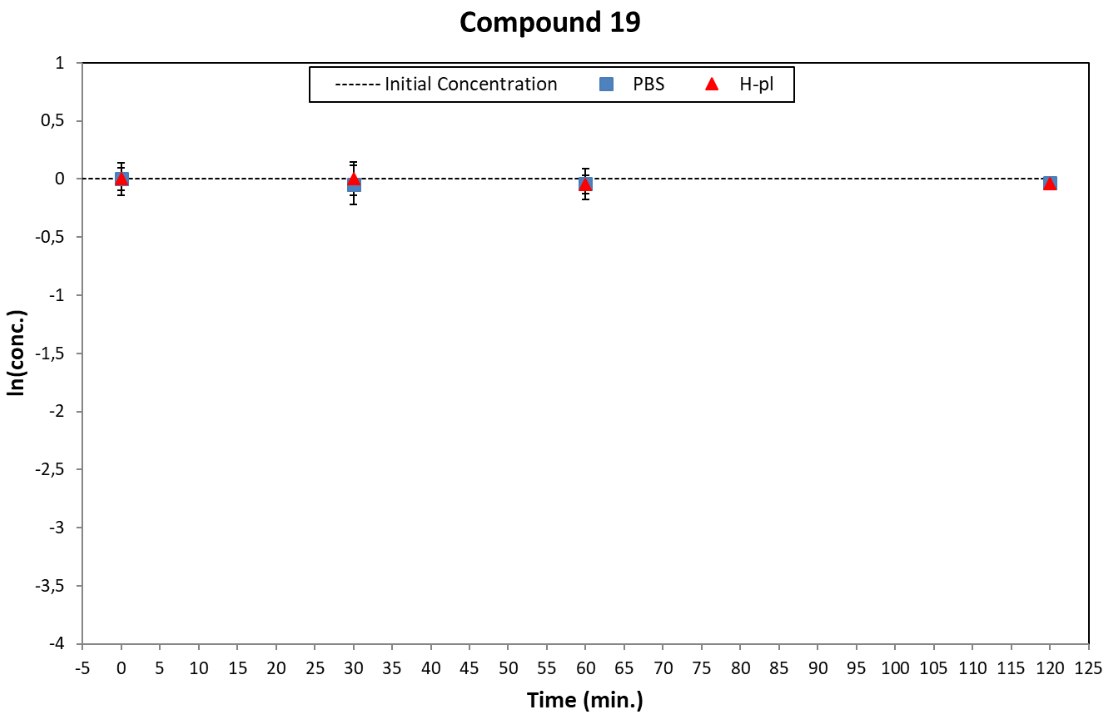

Figure S19: Degradation plots of **19** in PBS (blue square) and human plasma (red triangle).

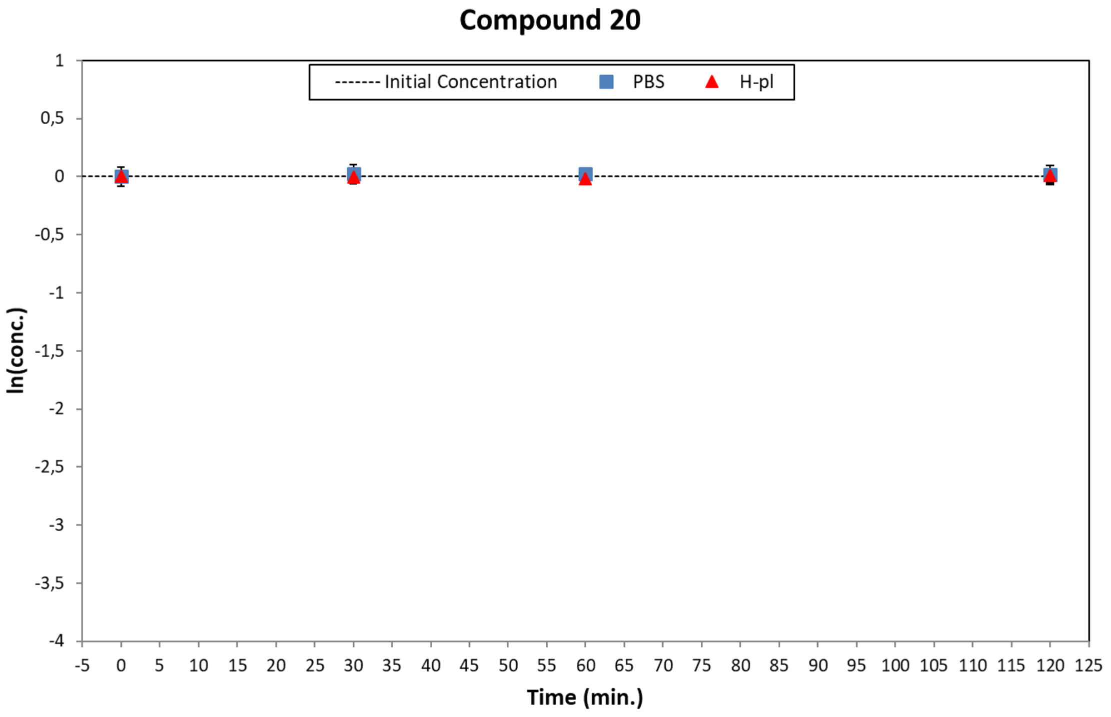

Figure S20: Degradation plots of **20** in PBS (blue square) and human plasma (red triangle).

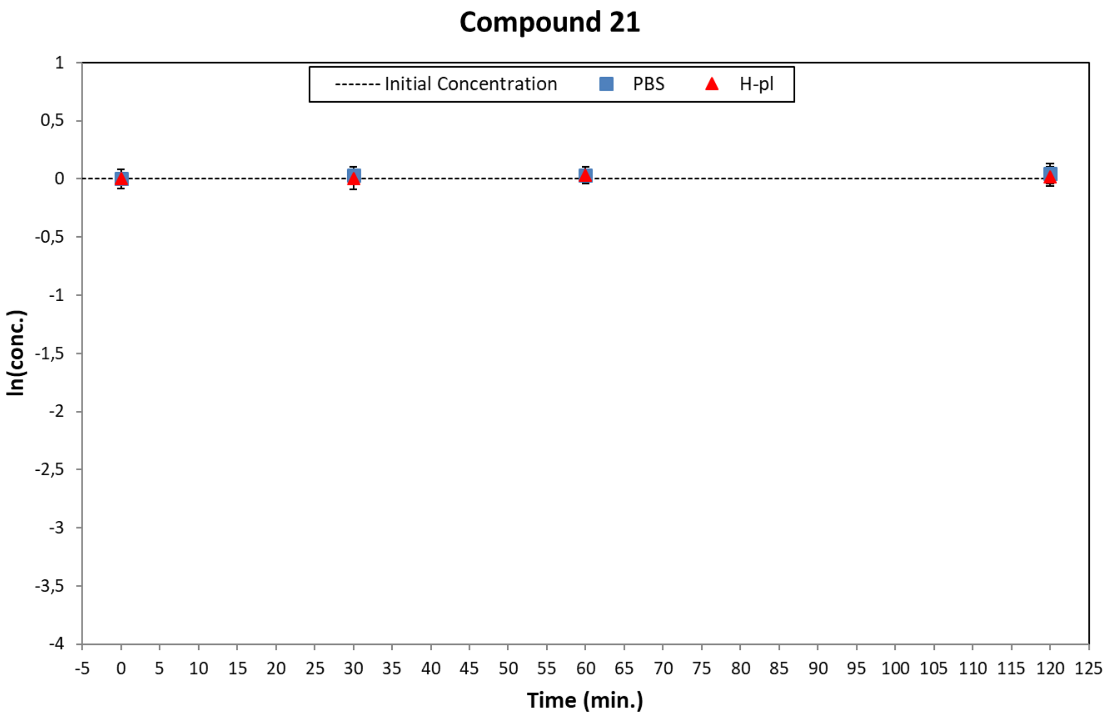

Figure S21: Degradation plots of **21** in PBS (blue square) and human plasma (red triangle).

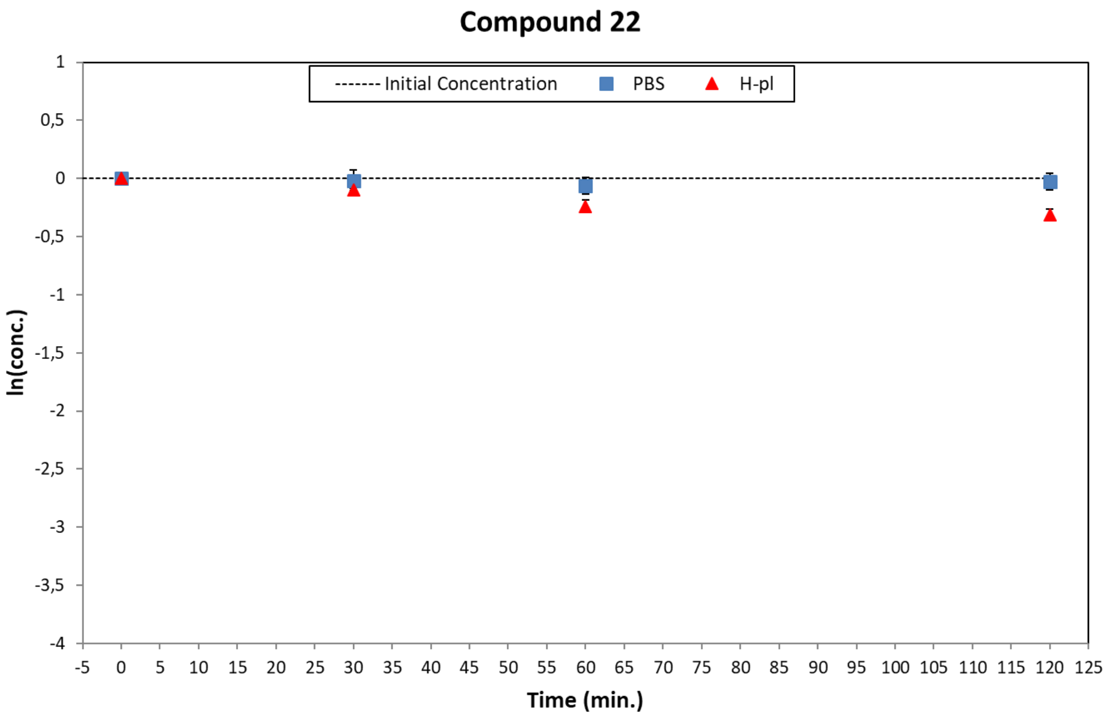

Figure S22: Degradation plots of **22** in PBS (blue square) and human plasma (red triangle).

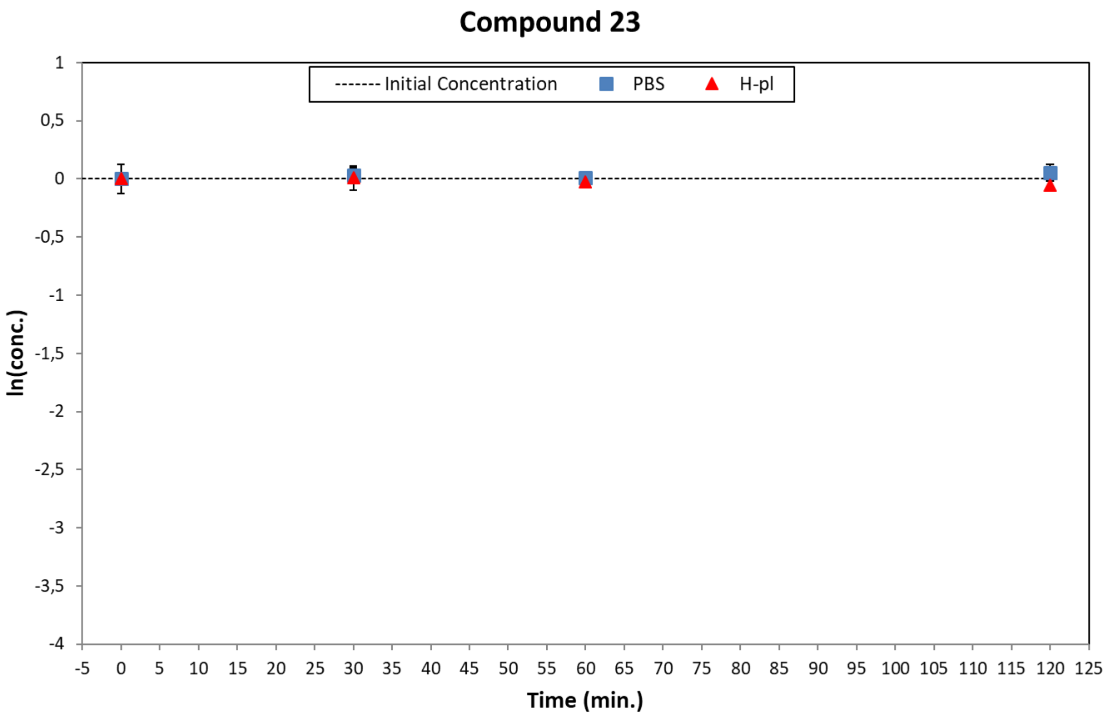

Figure S23: Degradation plots of **23** in PBS (blue square) and human plasma (red triangle).

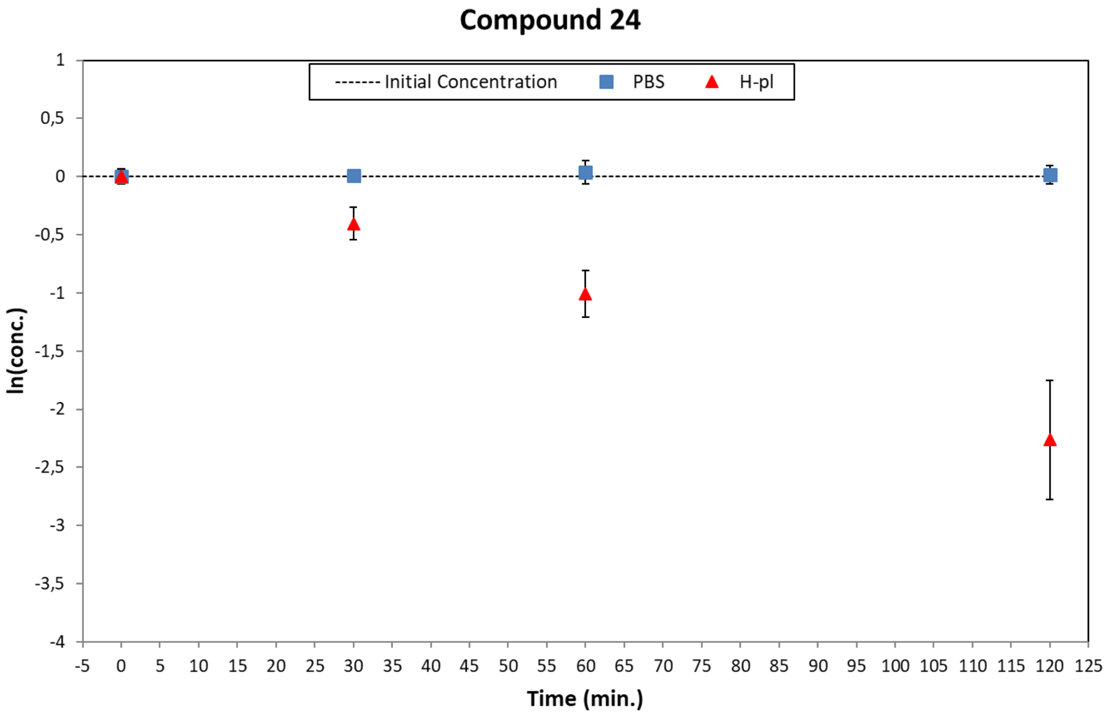

Figure S24: Degradation plots of **24** in PBS (blue square) and human plasma (red triangle).

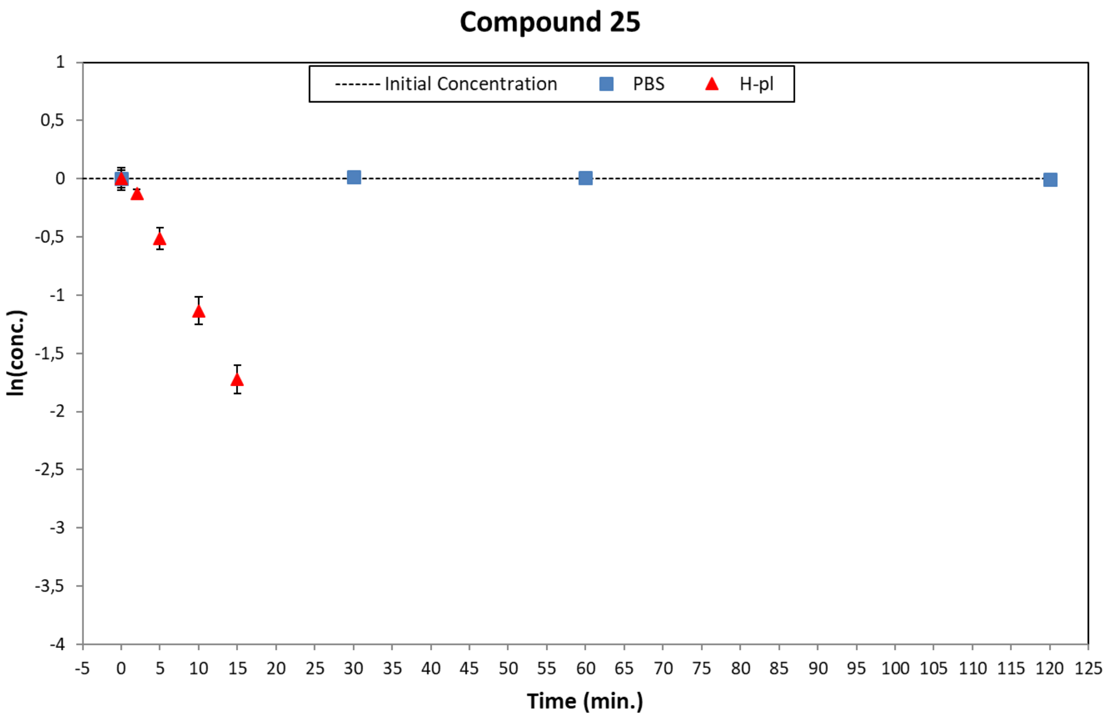

Figure S25: Degradation plots of **25** in PBS (blue square) and human plasma (red triangle).

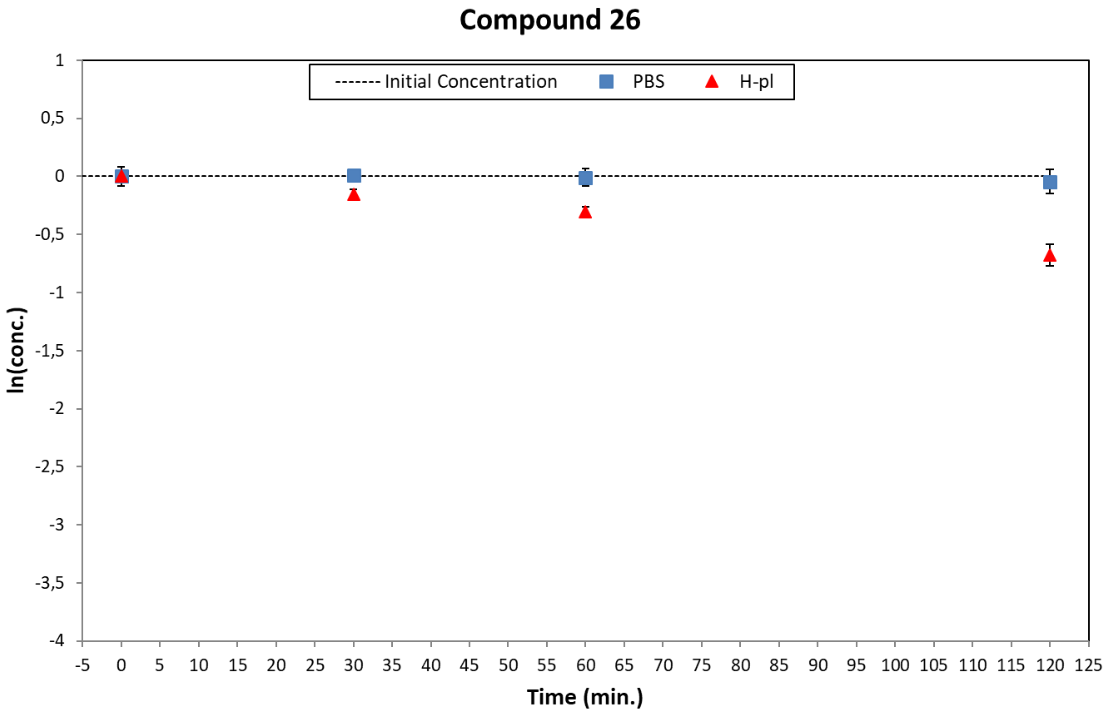

Figure S26: Degradation plots of **26** in PBS (blue square) and human plasma (red triangle).

P-gp, MRP1 and BCRP expression in MDCK, MDCK-MDR1, MDCK-MRP1 and MDCK-BCRP cells

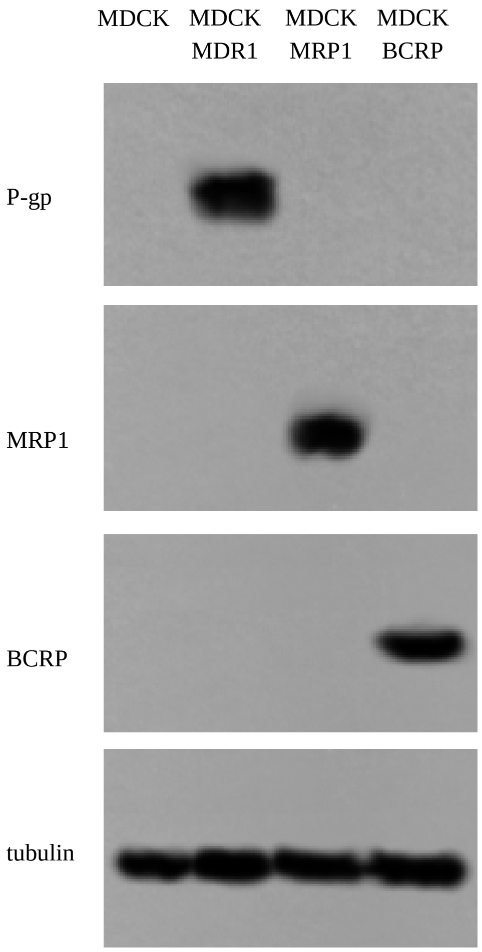

Figure S27. Immunoblotting analysis of P-gp, MRP1 and BCRP expression in MDCK, MDCK-MDR1, MDCK-MRP1 and MDCK-BCRP cells. Tubulin was used as control of equal protein loading.
